# Machine Learning-Based Prediction of Phase Equilibria and Interfacial Properties of Multicomponent CO_2_ Mixtures with Impurities: Application to CO_2_ Transportation

**DOI:** 10.1021/acs.iecr.6c03388

**Published:** 2026-08-28

**Authors:** Darshan Raju, Roar Skartlien, Mahinder Ramdin, Thijs J. H. Vlugt

**Affiliations:** † Engineering Thermodynamics, Process & Energy Department, Faculty of Mechanical Engineering, 2860 Delft University of Technology, Leeghwaterstraat 39, Delft 2628 CB, The Netherlands; ‡ Institute for Energy Technology, P.O. Box 40, Kjeller N-2027, Norway

## Abstract

Impurities in captured CO_2_ broaden the two-phase envelope, decrease the interfacial tension (IFT), and affect safe dense-phase transport. These properties are difficult to measure and expensive to compute using molecular simulations. We present the first systematic machine learning (ML) surrogate framework for phase equilibria and interfacial properties of impure CO_2_, trained on Perturbed-Chain Polar Statistical Associating Fluid Theory (PCP-SAFT) equation of state (EoS) + cDFT data. Within this framework, the hyperparameter-optimized TabPFN most accurately predicts *P*_bubble_, *P*_dew_, IFT, and interfacial thickness. For IFT, we combined the Winterfeld–Scriven–Davis correlation with an ML-based residual correction. The hybrid model with stochastic variational Gaussian process regression reduces the root mean squared error (RMSE) of IFT to 0.02 mN m^–1^. Symbolic regression additionally provides an interpretable expression for this correction. For sampling CO_2_-rich compositions, active learning is the most data-efficient strategy. Trained surrogates predict each state point in ca. 1–5 ms, compared to ca. 0.18–1.8 s for cDFT, enabling rapid property estimation for computational fluid dynamics (CFD), inverse design, and the planning of simulations and experiments.

## Graphical abstract

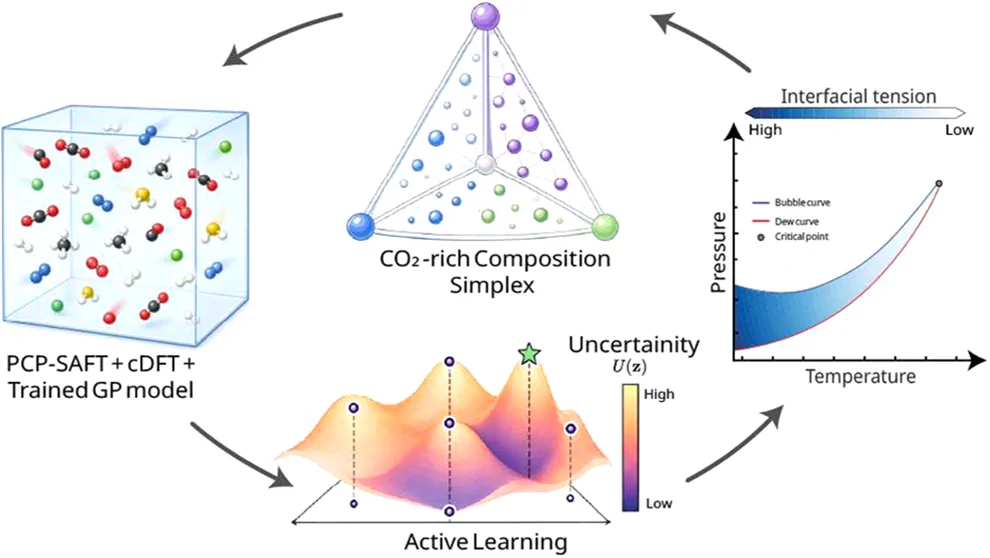

## 1 Introduction

Carbon dioxide (CO_2_) is one of the major greenhouse gases that drive global warming.^1,2^ Carbon capture has become an essential process to reduce CO_2_ emissions and to prevent global warming.^3,4^ Techniques related to the CO_2_ capture, transport, storage, and utilization of CO_2_ are collectively referred to as CCUS. The transportation of CO_2_ is essential for multiple CCUS applications, including direct air capture,^5^ enhanced oil recovery,^6^ methanol production,^6^ and dedicated reservoir storage (e.g., Northern Lights, Porthos).^7,8^ CO_2_ is transported efficiently from capture facilities to storage reservoirs by pipelines or ships.^9^ CO_2_ obtained from capture processes is typically not pure. The composition of the captured CO_2_ depends on the capture technology and the source stream, such as flue gas in postcombustion capture, syngas in precombustion capture, and oxy-fuel flue gas in oxy-combustion capture.^10^ The impurities found primarily in postcombustion are typically N_2_, O_2_, and Ar, whereas in oxy-fuel combustion, high concentrations of Ar, N_2_, and O_2_ are found.^6,7,11–13^ In precombustion, the impurities are mostly H_2_, CH_4_, and CO.^6,7,11–13^ Along with the noncondensable impurities such as Ar, N_2_, H_2_, CH_4_, O_2_, and CO,^6,7,11–13^ minor traces of corrosive impurities such as H_2_O, H_2_S, SO_*x*_, NO_*x*_, O_2_, and hydrocarbons (C_2_H_6_ and C_3_H_8_) are also found in the captured source streams. These impurities affect the thermophysical behavior and phase stability of the CO_2_ stream by broadening the two-phase envelope. For efficient transportation, CO_2_ streams must be maintained in a dense single phase.^14–16^ This reduces the volumetric flow rate and helps prevent phase separation, which can otherwise cause flow instability and pressure losses.^14,15^ This requirement is particularly challenging during transient operations, where pressure and temperature fluctuate.^10,17^ While steady-state operation targets a dense phase, systems often undergo startup, shutdown, depressurization, and planned maintenance, which can lead to two-phase flow in CO_2_ transportation systems.^18–20^ At such conditions, accurate knowledge of the thermodynamic and transport properties of impure CO_2_ mixtures becomes indispensable for modeling their flow and phase behavior.^10,21^ Compared to other thermodynamic and transport properties such as density, heat capacity, speed of sound, and viscosity, interfacial tension (IFT) is one of the important properties of the fluid mixture that governs the bubble and droplet formation and stability, and interphase heat and mass transfer.^22–24^ During depressurization of the CO_2_ stream through a valve or nozzle, the CO_2_ flow may experience a delayed phase change during that fast transient event.^20^ The delayed phase change needs to be accounted for in computational fluid dynamics (CFD) simulations to correctly predict mass flow rate and pressure, thereby enabling the accurate evaluation of pipeline forces and the associated risk of running ductile fracture.^23,25^

Experimental measurements and molecular dynamics (MD) simulations are widely used to determine phase equilibria and interfacial properties.^24–28^ Experimental determination of IFTs for a wide range of mixtures and compositions encountered in CO_2_ transportation is costly and time-consuming, resulting in limited and scarce experimental data for impure CO_2_ mixtures.^10,29–32^ MD simulations using classical force fields have proven to be a powerful tool to predict interfacial properties.^24–26,32^ Performing MD simulation for a wide range of compositions encompassing a broad range of two-phase conditions to compute interfacial properties can be extremely computationally intensive.^25^ In addition to experimental measurements and MD-based computations, equation of state (EoS)-based interfacial modeling approaches, including classical Density Functional Theory (cDFT), density gradient theory (DGT), square gradient theory (SGT), linear gradient theory (LGT), and semiempirical correlations, have been used to predict interfacial properties.^25,27,33,34^ The EoS-based cDFT, DGT, SGT, and LGT models closely reproduce experimental measurements^27,35^ and MD simulation results.^25,36–38^ To illustrate a few, Chow et al.^27^ used the Statistical Associating Fluid Theory (SAFT)-variable range (VR) Mie EoS^39^ coupled with SGT to predict the IFT of binary and ternary mixtures containing H_2_O, CO_2_, Ar, and N_2_. The predictions closely reproduced the experimental measurements, and the SAFT-VR Mie EoS + SGT framework was recommended for modeling the IFTs of H_2_O–CO_2_ systems containing diluent gases. In our previous study,^25^ we computed the interfacial properties of binary CO_2_ mixtures containing Ar, N_2_, CH_4_, and H_2_. These properties included the IFT, interfacial thickness, and impurity enrichment at the interface. The computations were performed using MD simulations and the Perturbed-Chain Statistical Associating Fluid Theory (PC-SAFT) EoS + cDFT framework, and the results were compared with predictions from the semiempirical Parachor^40^ and Winterfeld–Scriven–Davis (WSD)^41^ models. The PC-SAFT EoS + cDFT with temperature-dependent binary interaction parameters (BIPs) showed a good agreement with MD simulations, and both the Parachor and the WSD semiempirical models were found to have significant deviations. A wide range of empirical and semiempirical models has been proposed in the literature to model the IFTs of mixtures, differing in their physical basis, flexibility, and number of adjustable or fitted coefficients. In a recent comparative study, Mulero et al.^42^ assessed 10 empirical and semiempirical models for binary *n*-alkane mixtures and reported that the WSD model^41^ yields the best overall performance for estimating IFT, provided that all components in the mixture are subcritical. When this condition is not satisfied, the Redlich–Kister model^43^ was found to perform best. However, the WSD model requires only one fit coefficient, whereas the Redlich–Kister model requires three. Similar limitations in terms of requiring fitted coefficients and condition-dependent performance are also observed in other semiempirical models reported by Mulero et al.,^42^ particularly when one or a few components in the mixture are in a supercritical state.^44^ Complementing these traditional modeling approaches, machine learning (ML) has emerged as a promising alternative by leveraging available thermophysical data.^45^ Maginn and co-workers^45^ used the CRC Handbook of Chemistry and Physics^46^ as the primary experimental source to train the residuals obtained from the Joback and Reid (JR) Group Contribution (GC) method. The computationally efficient JR GC method often shows systematic biases (residuals). The systematic biases of normal boiling temperature (*T*_b_), enthalpy of vaporization at *T*_b_ (Δ*H*_vap,*T*_b__), normal melting temperature (*T*_m_), critical pressure (*P*_c_), critical molar volume (*V*_c_), and critical temperature (*T*_c_) are compared to the experimental source^46^ of 485–5640 molecules. The computed residuals were then trained on the Gaussian Process Regression (GPR) model to improve the accuracy of thermodynamic property predictions using the JR GC method and to quantify the associated uncertainties.^45^ The hybrid model achieved predictive performance of a coefficient of determination (*R* ^2^) > 0.85 for *T*_b_, Δ*H*_vap,*T*_b__, *P*_c_, *V*_c_, and *T*_c_.^45^ Focused on IFT, Mouallem et al.^47^ developed ML models to estimate CO_2_-brine IFT for CO_2_ geo-storage using a large literature data set of 2896 experimental data points covering wide ranges of pressure, temperature, salinity, salt type, and CH_4_ and N_2_ impurities. These authors compared Gradient Boosting (GB), eXtreme Gradient Boosting (XGB), Least Squares Boosting (LSB), GPR, Artificial Neural Networks (ANN), and Genetic Programming, and found that GB performed the best with *R* ^2^ = 0.964. When a suitable database is unavailable or nonexistent, numerous studies have used physics-based models, such as molecular simulations and EoSs, to generate representative data sets for application-specific compositions and thermodynamic conditions.^47–50^ Albá et al.^48^ used the polar soft-SAFT EoS to generate saturation properties data for CO_2_-based refrigerant mixtures and trained an ANN to predict bubble and dew-point temperatures. The ANN model achieved *R* ^2^ ≈ 1, demonstrating its suitability for screening sustainable refrigerant mixtures.^48^ It is important to note that purely data-driven ML models do not always follow the expected physical behavior. This is especially important for tree-based models, which typically produce piecewise-constant predictions and have limited ability to extrapolate beyond the training range. As a result, these models may not fully capture the smooth variation of thermophysical properties, such as IFT, with temperature and pressure. This motivates the use of physics-informed hybrid models, where a physically based model first describes the main trend, and the ML model is then used to learn only the remaining residual error. Although several ML models have been developed to predict the IFT of CO_2_–brine systems^47,51–53^ and other thermophysical properties of CO_2_-based mixtures,^54–56^ including saturation properties,^48^ a comparative assessment of ML models for CO_2_-rich mixtures containing transportation-relevant impurities is still lacking. Such an assessment first requires a training data set, and building that data set dominates the cost because every point has to be generated by a physics-based computation, and the composition space of CO_2_ transport is high-dimensional and thinly populated. The number of feed compositions required to reach a target accuracy, therefore, determines the computational cost of training ML models, and the most efficient sampling strategy for these compositions has not been established for CO_2_-rich mixtures.

In this study, we use the Perturbed-Chain Polar Statistical Associating Fluid Theory (PCP-SAFT) EoS^57–59^ combined with cDFT^60^ to compute the IFT, interfacial thickness, bubble-point pressure, and dew-point pressure of multicomponent CO_2_-rich mixtures containing transportation-relevant impurities. The CO_2_ mole fraction ranges from 95 to 99.99%, while the total impurity concentration varies from 5 to 0.01%. This concentration range reflects practical CO_2_ transportation conditions,^7^ where high CO_2_ purity is desired to maintain dense-phase operation and reduce the risk of phase splitting and flow-assurance problems.^6,7,10^ The impurities considered are Ar, N_2_, CH_4_, H_2_, O_2_, CO, and H_2_S. Other impurities, such as H_2_O, NO_*x*_, and SO_*x*_, are typically present at parts-per-million levels and are therefore not included.^6,10^ The inclusion of these impurities enables the model to cover the main classes of transportation-relevant contaminants, including light gases, noncondensable components, and acid–gas impurities.^6,10^ The systems considered range from binary to 8-component mixtures. The PCP-SAFT EoS predicts the phase equilibria and interfacial properties of CO_2_-rich mixtures in close agreement with experimental data, yielding bubble- and dew-point pressures comparable to those obtained from the GERG-2008 EoS.^61^ This agreement supports its use for generating the phase-equilibrium and interfacial-property training data. Using the generated PCP-SAFT EoS + cDFT^57,62^ data set, we develop data-driven ML models for four target properties: bubble-point pressure, dew-point pressure, IFT, and interfacial thickness. We compare several ML models, including Support Vector Machine (SVM), Random Forest (RF), XGB, and Tabular Prior-data Fitted Network (TabPFN). For all target properties, we use the same input features: temperature, pressure, and mixture composition. These features correspond to the input variables required when planning experiments or molecular simulations. We found that TabPFN with HyperParameter Optimization (HPO), followed by XGB, achieved the lowest Cross Validation (CV) root-mean-square error (RMSE) and mean absolute error (MAE) for all target properties. The results also showed that dew-point pressure and interfacial thickness are less sensitive to impurity concentration. We further assessed the effect of mixture size and compared random, stratified, and GPR-based active learning (AL) sampling strategies. AL was the most data-efficient sampling strategy, requiring fewer feed compositions to achieve a given accuracy. We further develop a hybrid ML model for IFT using the computationally efficient WSD model as a baseline. The ML model is trained on the deviation of the WSD model from the more rigorous PCP-SAFT EoS+cDFT predictions. The ML models used to develop the IFT hybrid models are Symbolic Regression (SR), GPR, and Stochastic Variational Gaussian Process Regression (SVGP). This hybrid strategy enables rapid estimation of IFTs for multicomponent CO_2_-rich mixtures relevant to CO_2_ transportation. After training, the surrogate models can predict interfacial and saturation properties much more rapidly than direct PCP-SAFT EoS + cDFT calculations. This reduction in computational cost is the central motivation of the present study. Multiphase CFD simulations of CO_2_ transport require repeated evaluations of interfacial and saturation properties over a wide range of compositions and thermodynamic states, which become prohibitively expensive when using direct PCP-SAFT EoS + cDFT or MD computations.^20,23^ In sharp contrast, once trained, the ML surrogates can provide these properties at negligible computational cost, making them suitable for repeated property evaluations in large-scale transport process simulations. To the best of our knowledge, this is the first study to combine the PCP-SAFT EoS + cDFT physics engine with two complementary ML surrogate strategies for the IFT of multicomponent CO_2_-rich mixtures, while also developing fully data-driven ML models for phase equilibria. The two strategies are a fully data-driven model and a physics-informed WSD-residual model. Earlier ML studies are typically limited to a single property or to simple binary or CO_2_–brine systems. In contrast, our framework spans binary to eight-component mixtures and includes seven CO_2_ transportation-relevant impurities. It also provides uncertainty-aware predictions and active-learning-based composition sampling.

The paper is organized as follows: we describe the methodology used to compute the saturation properties (bubble-point and dew-point pressures) and interfacial properties using the PCP-SAFT EoS + cDFT in Section 2. The WSD semiempirical model and ML models used in the study are also explained briefly in Section 2. In Section 3, we present the predictive performance of the ML models for each target property. We also analyze the effects of composition space and mixture size and evaluate the performance of the hybrid residual ML model relative to the semiempirical WSD model. Finally, Section 4 summarizes the main findings of this work. Further methodological details and results are provided in the Supporting Information. The experimental phase equilibrium data, the training data sets, and the trained ML models used in this work are provided in the accompanying Supporting Information.

## 2 Methodology

### 2.1 Classical Density Functional Theory + PCP-SAFT

The phase equilibria (bubble and dew-point pressures) represented in the *PT* phase diagram, and the interfacial properties of multicomponent CO_2_ mixtures with impurities are computed using the Framework for Equations of State and Classical Density Functional Theory (FeOs) v0.9.5 framework.^60^ In FeOs, SAFT-based EoSs, such as PCP-SAFT and PC-SAFT,^57–59,63^ are coupled with cDFT^62,64–66^ to model inhomogeneous systems, including vapor–liquid interfaces.^60,62^ In cDFT, the vapor–liquid interface is treated as a mesoscopic region in which the density varies smoothly from the bulk vapor phase to the bulk liquid phase. The inhomogeneous system is described using the grand potential functional,^62^

1 $$\Omega \left(T,\mu ,\left[\rho \left(r\right)\right]\right)=F\left(T,\left[\rho \left(r\right)\right]\right)-\sum_{i}\int \rho_{i}\left(r\right)\left(\mu_{i}-V_{i}^{\mathrm{ext}}\left(r\right)\right)dr$$

where **r** is the position vector in space, ρ_*i*_(**r**) is the number-density profile of component *i*, [ρ­(**r**)] denotes the set of density profiles of all components in the mixture, *F*(*T*, [ρ­(**r**)]) is the intrinsic Helmholtz free-energy functional, μ_*i*_ is the chemical potential of component *i*, and *V*_*i*_^ext^(**r**) is an external potential acting on all or some molecules.^62^ The equilibrium density profiles are obtained by minimizing the grand potential with respect to the density profile at a constant temperature and chemical potential of each component,

2 $${\frac{\delta \Omega }{\delta \rho_{i}\left(r\right)}|}_{T,\mu_{i}}=0$$

Applying this equilibrium condition to the grand potential functional in eq 1 yields the Euler–Lagrange equation.^60^

3 $${\frac{\delta F\left[\rho \left(r\right)\right]}{\delta \rho_{i}\left(r\right)}|}_{T}-\mu_{i}+V_{i}^{\mathrm{ext}}\left(r\right)=0$$

For a homogeneous bulk system, *V*_*i*_^ext^(**r**) = 0, and the corresponding bulk relation is *F*_ρ__*i*_^b^ – μ_*i*_ = 0, where *F*_ρ__*i*_^b^ denotes the functional derivative of the Helmholtz free-energy functional evaluated for the bulk system. Using this bulk relation, the Euler–Lagrange equation^60^ in the absence of an external potential can be written relative to the bulk state as

4 $${\frac{\delta F\left[\rho \left(r\right)\right]}{\delta \rho_{i}\left(r\right)}|}_{T}-F_{\rho_{i}}^{b}=0$$

The intrinsic Helmholtz free-energy functional in the Euler–Lagrange equation is commonly decomposed into ideal and residual contributions, *F* [ρ­(**r**)] = *F* ^ideal^[ρ­(**r**)] + *F* ^res^[ρ­(**r**)], where the ideal contribution is known exactly from statistical mechanics, while the residual contribution accounts for intermolecular interactions.^62,67,68^ In PCP-SAFT-based cDFT, the residual Helmholtz free-energy functional can be decomposed into the same physical contributions as in the bulk PCP-SAFT EoS, namely, hard-sphere, hard-chain, and dispersion terms, and, when relevant, association and polar terms,^62,68^

5 $$F^{\mathrm{res}}\left[\rho \left(r\right)\right]=F^{\mathrm{hs}}\left[\rho \left(r\right)\right]+F^{\mathrm{chain}}\left[\rho \left(r\right)\right]+F^{\mathrm{disp}}\left[\rho \left(r\right)\right]+F^{\mathrm{assoc}}\left[\rho \left(r\right)\right]+F^{\mathrm{polar}}\left[\rho \left(r\right)\right]$$

For further details on the free-energy functional forms and cDFT, the reader is referred to refs 62,64,66,69 . In this study, the equilibrium density profiles are obtained by solving the Euler–Lagrange equation^60,64^ from eq 4, which combines the different contributions to the free-energy functional. Once the equilibrium density profiles are obtained, the IFTs are computed from the excess grand potential per unit interfacial area *A* as

6 $$\gamma =\frac{\Omega^{\mathrm{Eq}.}-\Omega^{\mathrm{Bulk}}}{A}$$

where Ω^EQ.^ and Ω^Bulk^ are the equilibrium and bulk grand potentials, respectively. The interfacial thickness (*L*_10_^90^), adsorption, and enrichment of components at the interface are computed from the density profiles.^25,70^ The Euler–Lagrange equations are solved numerically in one-dimensional Cartesian coordinates, and the required functional derivatives are evaluated by automatic differentiation.^33^ The interfacial domain is discretized with adaptive grid width and number of grid points, with interface widths ranging from 100 to 1000 Å and grid sizes from 1024 to 2048 points, where finer discretization is applied near the critical region. The density profiles are computed using the default cDFT solver settings in FeOs,^60^ which uses two sequential Anderson mixing steps^68,71^ with a relative density residual tolerance of 10^–11^. Although the implementation is computationally efficient in FeOs,^60^ the computation of interfacial properties for a full *PT* phase diagram can still be computationally demanding for multicomponent mixtures with temperature-dependent *k*_*ij*_. This is because the equilibrium density profiles are obtained from coupled nonlinear Euler–Lagrange equations, which must be solved iteratively over the spatial domain for all components in the mixture at each state point.^68,72–74^ This cost becomes particularly important when repeated IFT computations are required, as in CFD and other process simulations. The pure-component parameters of the PCP-SAFT EoS^57–59,63^ used to describe the bulk phase behavior and interfacial properties of mixtures comprising CO_2_, H_2_, N_2_, Ar, CH_4_, O_2_, CO, and H_2_S are *m*_*i*_, σ_*ii*_, ε_*ii*_/*k*_B_, *p*_*i*_, *Q*_*i*_, *n*_A_, *n*_B_, κ^AB^, and ε^AB^/*k*_B_. These parameters denote the number of spherical segments per molecule, segment size parameter, dispersion energy parameter, dipole moment, quadrupole moment, number of association sites of type A, number of association sites of type B, association volume, and association energy parameter, respectively. For the studied components, only H_2_S was modeled with an explicit association contribution using a 2A – 2B association scheme, with *n*_A_ = 2 and *n*_B_ = 2. Hence, cross-association interactions between different molecular species were not considered. The indices *ii* and *jj* denote interactions between segments of the same component, whereas interactions between dissimilar atom pairs were modeled using the Lorentz–Berthelot (LB) mixing rules.^75,76^

7 $$\sigma_{\mathrm{ij}}=\left(1-l_{\mathrm{ij}}\right)\frac{1}{2}\left(\sigma_{\mathrm{ii}}+\sigma_{\mathrm{jj}}\right)$$

8 $$\varepsilon_{\mathrm{ij}}=\left(1-k_{\mathrm{ij}}\right){\left(\varepsilon_{\mathrm{ii}}\varepsilon_{\mathrm{jj}}\right)}^{1/2}$$

where the indices *ij* refer to cross interactions between atoms of different types. The parameters *k*_*ij*_ and *l*_*ij*_ are the binary interaction parameters used in the mixing rules for ε_*ij*_ and σ_*ij*_, respectively, for components *i* and *j*. Here, *l*_*ij*_ is not considered (*l*_*ij*_ = 0), and only *k*_*ij*_ is considered. To accurately model the multicomponent CO_2_-rich mixtures, temperature-dependent BIPs (*k*_*ij*_) were determined for each dissimilar CO_2_-impurity pair by fitting to the available experimental *P*_*xy*_ data at a temperature where experimental data were available. Here, *P*_*xy*_ refers to isothermal vapor–liquid equilibrium data in which the equilibrium pressure, *P*, is reported together with the corresponding liquid-phase composition, *x*, and vapor-phase composition, *y*. The fitted BIPs, together with the pure-component PCP-SAFT EoS^57–59,63^ parameters used in this study, are provided in of the Supporting Information. These parameters were used to compute the phase equilibria and interfacial properties of CO_2_ mixtures with impurities. In addition, describes the method used to determine the temperature-dependent BIPs and lists the experimental *P*_*xy*_ data used for fitting. For an *N*-component mixture, *N*(*N* – 1)/2 BIPs are required, so the 8-component mixture considered in this study requires 28 binary pairs. Experimental *P*_*xy*_ data were available for 23 pairs. For the remaining five pairs, H_2_–O_2_, CH_4_–O_2_, O_2_–CO, Ar-H_2_S, and O_2_–H_2_S, no experimental *P*_*xy*_ data were available in the literature. Hence, *k*_*ij*_ was set to zero for these pairs.

### 2.2 Winterfeld–Scriven–Davis (WSD) Model

A theory for estimating the IFT of multicomponent fluid mixtures was formulated by Winterfeld et al.^41^ This theory extends the Fowler–Kirkwood–Buff framework developed^77,78^ by simplifying the statistical mechanics expression for the calculation of IFT with the assumption of pairwise additive intermolecular potentials.^41,77,78^ The resulting WSD model provides a practical method for estimating IFTs of nonaqueous mixtures using the IFTs of the pure components present in the mixture and can be generalized to an *N*-component mixture as,

9 $$\gamma_{\mathrm{mix}}\left(T,P,z\right)=\sum_{\alpha =1}^{N}\sum_{\beta =1}^{N}\left[{\left(\frac{\rho_{\alpha }^{L}-\rho_{\alpha }^{V}}{\rho_{\alpha }^{0,L}-\rho_{\alpha }^{0,V}}\right)}^{2}\gamma_{\alpha }^{0}\delta_{\alpha \beta }+\Phi_{\alpha \beta }\left(\frac{\rho_{\alpha }^{L}-\rho_{\alpha }^{V}}{\rho_{\alpha }^{0,L}-\rho_{\alpha }^{0,V}}\right)\left(\frac{\rho_{\beta }^{L}-\rho_{\beta }^{V}}{\rho_{\beta }^{0,L}-\rho_{\beta }^{0,V}}\right)\sqrt{\gamma_{\alpha }^{0}\gamma_{\beta }^{0}}\right]$$

where γ_mix_ denotes the mixture interfacial tension, which depends on temperature *T*, pressure *P*, and mole fraction vector **z** = (*z*_1_, *z*_2_, ···, *z*_*N*_). Here, *N* is the number of components in the mixture, and the indices α and β denote the components in the system. The double summation in eq 9 runs over all component pairs, with α, β = 1, 2, ···, *N*. The quantities γ_α_^0^ and γ_β_^0^ represent the pure-component IFTs of components α and β, respectively. Similarly, ρ_α_^0,L^, ρ_α_^0,V^, ρ_β_^0,L^, and ρ_β_^0,V^ correspond to the equilibrium liquid and vapor molar densities, in mol cm^–3^, of the pure components α and β, respectively. The parameters ρ_α_^L^, ρ_α_^V^, ρ_β_^L^, and ρ_β_^V^ denote the liquid and vapor-phase molar densities of components α and β in the mixture.^41^ The term Φ_αβ_ is the cross-interaction, or mixing, parameter that accounts for nonideal interactions between dissimilar molecules. For nonaqueous and weakly polar fluids, Φ_αβ_ is commonly approximated as 1 (Φ_αβ_ ≃ 1).^41^ The Kronecker delta δ_αβ_ equals one when α = β, corresponding to the pure-component contribution, and zero otherwise.^41^ For an *N*-component mixture, eq 9 can also be written compactly in matrix form as γ_mix_(*T*, *P*, **z**) = **a** ^T^Φ**a**, where the vector **a** contains the density-scaled pure component IFT terms, $a_{\alpha }=\left(\frac{\rho_{\alpha }^{L}-\rho_{\alpha }^{V}}{\rho_{\alpha }^{0,L}-\rho_{\alpha }^{0,V}}\right)\sqrt{\gamma_{\alpha }^{0}}$ , and Φ is the symmetric matrix of cross-interaction parameters with diagonal elements Φ_αα_ = 1. The WSD model has been shown to provide reliable IFT predictions for nonaqueous mixtures and therefore serves as a useful semiempirical framework when molecular-correlation data are unavailable.^25,41,42^ The formulation becomes less reliable when one or more components in the mixture are supercritical at *T*. For a three-component mixture, assuming Φ_αβ_ = 1 for the cross-interaction term and treating component 3 as supercritical, eq 9 simplifies to

10 $$\gamma_{\mathrm{mix}}\left(T,P,z_{1},z_{2},z_{3}\right)={\left(\frac{\rho_{1}^{L}-\rho_{1}^{V}}{\rho_{1}^{0,L}-\rho_{1}^{0,V}}\right)}^{2}\gamma_{1}^{0}+{\left(\frac{\rho_{2}^{L}-\rho_{2}^{V}}{\rho_{2}^{0,L}-\rho_{2}^{0,V}}\right)}^{2}\gamma_{2}^{0}+\left(\frac{\rho_{1}^{L}-\rho_{1}^{V}}{\rho_{1}^{0,L}-\rho_{1}^{0,V}}\right)\left(\frac{\rho_{2}^{L}-\rho_{2}^{V}}{\rho_{2}^{0,L}-\rho_{2}^{0,V}}\right)\sqrt{\gamma_{1}^{0}\gamma_{2}^{0}}$$

In this form, component 3 does not contribute directly to the IFT because its pure-component interfacial tension is not defined at supercritical conditions. As a consequence, the WSD model neglects the direct contribution of the supercritical component, which can be physically limiting because component 3 can still influence the mixture phase behavior, density difference, and interfacial properties. In practice, component 3 is expected to lower the IFT of the subcritical mixture by weakening the cohesive forces between unlike molecules, similar to the action of a surfactant. For the CO_2_-rich mixtures with impurities considered in this study, all impurity components are supercritical at the investigated conditions, except H_2_S. In future work, the Widom line^79,80^ could be explored as a thermodynamic criterion for distinguishing liquid-like and gas-like supercritical states and for estimating the effective contribution of supercritical components to the mixture IFT. To represent this behavior, in our previous study, we introduced a correction term^25^ for binary mixtures based on the total liquid-phase mole fraction of the supercritical components. Here, we will use the baseline WSD model eq 9 to compute IFT and then to train the residual-based hybrid ML model with respect to the PCP-SAFT EoS + cDFT.^57,60,63^

### 2.3 Machine Learning Models

The capabilities of ML models in predicting thermophysical properties of fluid mixtures considered in this study have been widely reported in the literature.^45,81–87^ Hence, for brevity, we do not provide a detailed description of each model examined. Instead, we focus on the rationale for selecting the ML models, kernels, and hyperparameters used in this work. For the target properties, namely, dew-point pressure, bubble-point pressure, IFT, and interfacial thickness, we use the same set of input features to avoid overfitting and enable a consistent comparison. The selected features are physically well motivated because the feature set contains only the thermodynamic state variables *T*, *P*, and **z**, which are the same variables used to define phase equilibrium and IFT measurements. The supervised ML models considered in this study include Support Vector Machine (SVM),^88,89^ Random Forest (RF),^90^ Gradient Boosting (XGB),^91,92^ and Tabular Prior-data Fitted Network (TabPFN).^85,86,93^ SVM uses kernel functions to construct linear or nonlinear models in the feature space. Although it is commonly used for classification, it can also be applied to regression through Support Vector Regression (SVR), which is suitable for continuous thermophysical property prediction.^89^ In SVR, the model fits a regression function like linear regression while allowing errors within a tolerance region called the ϵ-insensitive tube. Data points on or outside this tube are the support vectors and mainly determine the fitted model. In this work, SVR with a Radial Basis Function (RBF) kernel was used for all target properties. The main hyperparameters are the regularization parameter *C*, the tube width ϵ, and the kernel coefficient γ_RBF_.^89^ *C* controls the penalty for errors outside the tube, ϵ defines the error tolerance, and γ_RBF_ controls the length scale of the RBF kernel.^89^ RF is an ensemble ML method composed of multiple decision trees.^90^ Each tree is trained on a bootstrapped sample of the training data set, where data points are selected randomly with replacement. At each node, the optimal split is selected from a random subset of input features, reducing correlation over individual trees and improving model robustness. For regression, the final prediction is obtained by averaging the outputs of all trees. The key hyperparameters of the RF model include the total number of trees, *n*_estimators_, the maximum allowable depth of each tree, *d*_max_, the minimum sample count required to divide an internal node, *n*_split_, the minimum number of samples retained in a terminal leaf, *n*_leaf_, and the number of input features randomly evaluated when determining each split, *n*_features_.^90^ XGB is a gradient-boosted decision-tree algorithm in which trees are added sequentially to correct the errors of the previous model.^91,92^ At each iteration, a new tree is fitted to the negative gradient of the loss function, and its contribution is scaled by a learning rate. The final prediction is obtained by summing the contributions from all trees. Compared with Adaptive Boosting (AdaBoost),^94^ XGB uses a more general gradient-based optimization framework and includes regularization terms that help reduce overfitting. Compared with Categorical Boosting (CatBoost),^95^ XGB is commonly used for numerical tabular data, whereas CatBoost is particularly designed for data sets with categorical features. The main hyperparameters of XGB besides *n*_estimators_ and *d*_max_ are sample fraction, feature fraction, and regularization parameters.^91,92^ In addition to SVR and decision-tree-based models, this work uses the state-of-the-art Tabular Prior-data Fitted Network (TabPFN) deep-learning model.^85^ TabPFN is a transformer-based tabular foundation model designed for heterogeneous tabular data with different feature scales, missing values, and mixed data types, where conventional neural networks (NNs) often struggle, and tree-based models usually perform strongly.^85^ This makes TabPFN suitable for thermodynamic property prediction, where the inputs are structured state variables such as *T*, *P*, and **z**, and the targets are continuous phase-equilibrium and interfacial properties. TabPFN is pretrained on millions of synthetic tabular data sets and uses In-Context Learning (ICL), the same mechanism used in large language models, to make predictions on new data sets with little or no data set-specific training.^85^ As such, extensive hyperparameter tuning is not strictly required, although HPO using Bayesian optimization can further adapt the model to a specific data set.^85,93^ In this work, the TabPFN HPO was performed using *N*_trials_ = 100, where each trial sampled a different hyperparameter configuration, fitted the TabPFN model, and evaluated its performance using CV. Additionally, an RF-TabPFN hybrid model is also considered to combine the robustness of tree-based learning with the strong small-data prediction ability of TabPFN.^93^ The hyperparameter tuning procedures for SVM, RF, XGB, and TabPFN, together with the corresponding optimal hyperparameter values, are provided in Section S4 of the Supporting Information. The SVM and RF models were implemented using the scikit-learn^96^ ML library in Python, XGB was implemented using the XGBoost Python package,^92^ and TabPFN was implemented using the TabPFN-v2.5 Python package.^85,86^ The HPO of TabPFN and RF-TabPFN models were implemented using the TabPFN-v0.4 extensions package.^93^ All of the ML models were trained on CPUs, except for the TabPFN-based models, which were run on an NVIDIA H100 GPU.

In addition to data-driven ML models, a residual-learning strategy was used for the IFT. The computationally inexpensive WSD model^41^ was taken as the baseline, and ML models were trained to predict the residual between the IFT obtained from the PCP-SAFT EoS + cDFT^60^ computation and the WSD prediction.^25,41^ This residual represents the missing correction to the semiempirical WSD model. Three ML models were considered for this task: Symbolic Regression (SR),^97^ Gaussian Process Regression (GPR),^98^ and Stochastic Variational Gaussian Process Regression (SVGP).^87,99,100^ SR^97^ was used to examine whether the residual can be represented by a compact and physically interpretable analytical expression, especially for mixtures containing supercritical components where the WSD model may lack *T*, *P*, and composition-dependent effects. Following the SR framework of Cranmer,^97^ SR searches over mathematical expressions generated from selected features, operators, and numerical constants, and identifies expressions that balance predictive accuracy and complexity. The exact GPR was chosen because it is widely used to learn smooth, unknown residual functions while also providing predictive uncertainty.^45^ In GPR, the residual function is described by a Gaussian process (GP) with a mean function *m*(**x**) and a covariance kernel *k*(**x**, **x**′).^45,98^ Here, **x** and **x**′ denote two input feature vectors, such as thermodynamic states defined by *T*, *P*, and composition **z**. In this work, the Matérn-5/2 kernel was used, with the main hyperparameters being the kernel variance σ_f_^2^, the length scale l , and the noise variance σ_n_^2^.^45,98^ These parameters control the magnitude of the residual variation, the smoothness of the residual function, and the unresolved numerical or modeling noise, respectively, and were optimized by maximizing the Log Marginal Likelihood (LML).^45,98^ Since the standard or exact GPR requires building and factorizing on the full *N* × *N* kernel matrix, its training time scales approximately as $O\left(N^{3}\right)$ and its memory requirement as $O\left(N^{2}\right)$ , where *N* is the number of training points.^98^ To improve scalability, Stochastic Variational Gaussian Process Regression (SVGP)^87,99,100^ was also used as an approximate GP model based on *M* inducing points, where *M* ≪ *N*. The SVGP model used a constant mean function and a Matérn-5/2 kernel with Automatic Relevance Determination (ARD), allowing each input feature to have an individual length scale and thereby providing a measure of feature relevance. A Cholesky variational distribution^99^ was used to approximate the posterior distribution over the inducing function values, and the inducing locations were optimized during training. The model was trained using the Evidence Lower Bound Objective (ELBO),^101,102^ which balances a data-fit term with a regularization term based on the Kullback–Leibler (KL) divergence^103^ between the variational posterior and the prior.^87,100^ To account for the highly skewed residual distribution, 2000 inducing points were initialized through stratified sampling. This sampling covered the *PT* region where most residuals are concentrated, as well as the midtail and extreme-tail residual regions. The target residuals were transformed using a quantile transformation followed by standardization, and the ELBO was maximized using mini-batch Adam optimization with a batch size of 1024, tail-weighted sampling, over 500 epochs, with the learning rate cosine-annealed from 10^–2^ to 10^–4^. The SVGP model was implemented using the GPyTorch package,^104^ while the exact GPR model was implemented using the scikit-learn library in Python.^96^ For SR, the symbolic-regression framework-v1.5 of Cranmer^97^ was used in Julia.

### 2.4 Composition Space Sampling

To compute the saturation pressures, namely, bubble and dew pressures, IFT, and interfacial thickness for compositions relevant to CO_2_ transportation, a pool of mixture compositions was generated. Since transported CO_2_ streams typically contain high CO_2_ mole fractions, the composition space was mainly focused on mixtures with *z*_CO_2__ in the range of 95–99.99%, while the remaining fraction was distributed between the impurity components. To distribute the impurity concentration, we used Dirichlet sampling,^105^ where the CO_2_ mole fraction was fixed initially, and the remaining fraction was treated as the impurity budget, *s* = 1 – *z*_CO_2__. The Dirichlet distribution is defined on a simplex, which is the geometric space of non-negative composition vectors whose components sum to unity. As a result, for *k* impurity components, the sampled normalized impurity vector lies on a (*k* – 1)-dimensional simplex.^105^ In this study, with seven impurity components considered, the Dirichlet distribution samples points on a six-dimensional impurity simplex. A normalized impurity composition vector was then sampled from a Dirichlet distribution,^105^

11 $$\left({\mathrm{z̃}}_{1},\cdot \cdot \cdot ,{\mathrm{z̃}}_{k}\right)∼\mathrm{Dir}\left(\alpha_{1},\cdot \cdot \cdot ,\alpha_{k}\right),,{\mathrm{z̃}}_{i}\geq 0,\sum_{i=1}^{k}{\mathrm{z̃}}_{i}=1$$

where *k* is the number of impurity components. The impurity mole fractions were obtained by scaling this normalized vector with the impurity budget,

12 $$z_{i}=s{\mathrm{z̃}}_{i}=\left(1-z_{{\mathrm{CO}}_{2}}\right){\mathrm{z̃}}_{i},,i=1,\cdot \cdot \cdot ,k$$

Thus, the final composition of a mixture satisfies

13 $$z_{{\mathrm{CO}}_{2}}+\sum_{i=1}^{k}z_{i}=z_{{\mathrm{CO}}_{2}}+\left(1-z_{{\mathrm{CO}}_{2}}\right)\sum_{i=1}^{k}{\mathrm{z̃}}_{i}=z_{{\mathrm{CO}}_{2}}+\left(1-z_{{\mathrm{CO}}_{2}}\right)=1$$

This procedure ensures that all impurity mole fractions are non-negative, the impurity fractions sum exactly to the prescribed remaining fraction, and the CO_2_ mole fraction remains fixed at the selected level. Apart from Dirichlet sampling,^105^ systematic grid scaling was used to ensure coverage of highly impure CO_2_ conditions, corresponding to *z*_CO_2__ = 95–96% with an impurity combination (binary mixtures). The final composition pool was generated using 70% Dirichlet sampling, 20% systematic grid scaling, and 10% compositions from industrial specifications and reported CO_2_ transport streams.^6^ The industrial compositions were taken from the review article of Simonsen et al.,^6^ including quality specifications from transportation projects such as Northern Lights^8^ and Porthos,^7^ capture technologies such as postcombustion, precombustion, oxy-fuel combustion, and operational CO_2_ pipelines in the USA and Canada.^6^ Trace impurities reported in parts per million, such as SO_*x*_, NO_*x*_, and NH_3_, were neglected, and their fractions were assigned to CO_2_. Since seven impurities were considered in this study, the possible combinations of impurity types and their concentrations are numerous. To address this, three sampling strategies, namely, random sampling, stratified sampling, and active learning (AL), were used to circumvent the computational cost of evaluating every mixture in the composition space and to identify the minimum number of mixtures required for reliable ML model performance. For random sampling, a target set of S mixture compositions was selected from the full composition pool of size P using a fixed random seed. The pool P contained compositions generated from Dirichlet sampling, systematic grid scaling, and industrially reported feed specifications. For stratified sampling, the CO_2_ mole fraction range was divided into five bins. Within each CO_2_ bin, the impurity compositions were further partitioned into five clusters using *k*-means clustering based on Euclidean distance.^106^ The number of impurity clusters was selected based on the elbow method and the silhouette score.^106–108^ This procedure resulted in 25 strata, combining 5 CO_2_ composition bins with 5 impurity composition clusters. The stratified sampling algorithm and its detailed implementation are provided in Section S3 of the Supporting Information. In addition to random and stratified sampling, a kernel-based AL scheme was adopted to reduce the computational cost associated with evaluating a large number of candidate mixtures in the composition pool P . The AL approach used in this work is shown in Algorithm 1. This approach was adapted from the work of Xiang et al.,^109^ where it was originally applied to chemical compound-space exploration of alkanes. In the present work, the algorithm was modified for composition-space sampling. A GP regressor with an ARD Matérn-5/2 kernel, signal variance σ_f_^2^, and additive noise variance σ_n_^2^ was first fitted to the pre-existing *P*_bubble_ labeled set $T_{\mathrm{lab}}$ using standardized composition vectors x̃ . The vector x̃ denotes the scaled form of the previously generated composition vector, obtained by mean-centering each composition variable and normalizing it by its standard deviation before fitting the GPR model.^98^ Xiang et al.^109^ similarly used the previously trained GPR model^110^ on MD simulations and the NIST Standard Reference Database^111^ with the marginalized graph kernel. However, in this work, we generated a composition pool of size 100 and computed the *P*_bubble_ curve using the PCP-SAFT EoS^57,63^ to train the GPR^98^ model and determine its hyperparameters. The kernel hyperparameters were obtained by maximizing the LML, giving $\theta^{*}=\left\{l^{*},\sigma_{f}^{2*},\sigma_{n}^{2*}\right\}.$ The optimized component-wise length scales provide a measure of the relevance of each species to *P*_bubble_. The fitted noise term was then discarded, retaining only the signal kernel $k_{s}\left({\mathrm{x̃}}_{i},{\mathrm{x̃}}_{j}\right)=\sigma_{f}^{2*}M_{5/2}\left({\mathrm{x̃}}_{i},{\mathrm{x̃}}_{j};l^{*}\right),$ which defines an ARD-weighted metric in the composition space. Starting from two randomly selected compositions, the selected set S was grown by adding the candidate with the largest posterior variance (uncertainty measure), defined as $U\left(x_{i}\right)=k_{s}\left({\mathrm{x̃}}_{i},{\mathrm{x̃}}_{i}\right)-k_{S}{\left({\mathrm{x̃}}_{i}\right)}^{T}K_{\mathrm{SS}}^{-1}k_{S}\left({\mathrm{x̃}}_{i}\right).$ This uncertainty depends on the position of each candidate mixture relative to the compositions already selected in S and does not require additional PCP-SAFT EoS^57,63^ evaluations during the selection step. For the Matérn-5/2 kernel, this procedure corresponds to a sequential maximum-variance design that selects new points from regions of composition space least covered by the current selected set. After each acquisition, candidates with *U* < *u*_t_ were removed from the remaining pool $P^{\prime }$ because they were considered informationally redundant. In contrast to Xiang et al.,^109^ who used a fixed uncertainty threshold *u*_t_ as the stopping criterion, *u*_t_ was adjusted in this work according to the target sample size *N*, so that the AL loop terminates when the selected set reaches |S|=N. The candidate pool P consisted of 1000 multicomponent CO_2_ mixtures. An initial subset, denoted as $P_{\mathrm{init}}$ , containing 100 multicomponent CO_2_ mixtures was generated from this pool and labeled by computing *P*_bubble_ using PCP-SAFT EoS.^57,63^ These labeled compositions were then used to pretrain the GPR model used in the acquisition step. The same candidate pool P was used for AL, random sampling, and stratified sampling. Each sampling strategy was repeated for 20 independent trials. The reported metrics correspond to the trial-averaged values, and the uncertainty is quantified using the 95% confidence interval for the independent trials. The temperature, pressure, and composition ranges spanned by the resulting training data set are summarized in Table 1. Predictions outside these ranges constitute extrapolation, for which the reported error metrics do not apply.
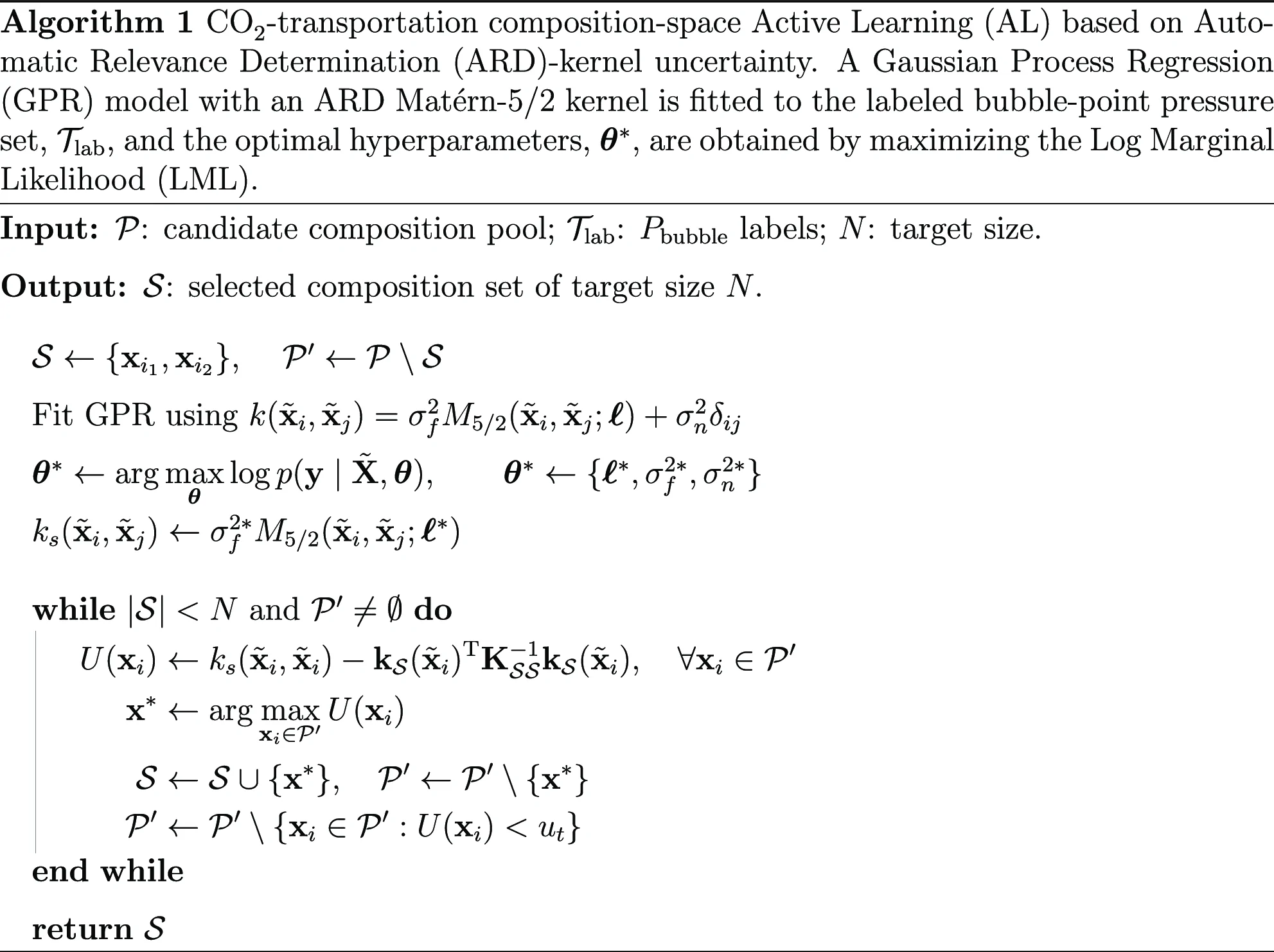


**Table 1:** Temperature, Pressure, and Composition Ranges of the Data Set Used to Train the ML Models, Generated Using the PCP-SAFT EoS + cDFT Framework^a^

variable	minimum	maximum
*T*/[K]	200	300
*P*/[bar]	2.62	162.88
*z*_CO_2__/[mol mol^–1^]	0.95	0.99
*z*_H_2__/[mol mol^–1^]	0	0.0313
*z*_Ar_/[mol mol^–1^]	0	0.05
*z*_N_2__/[mol mol^–1^]	0	0.04
*z*_CH_4__/[mol mol^–1^]	0	0.05
*z*_O_2__/[mol mol^–1^]	0	0.04
*z*_CO_/[mol mol^–1^]	0	0.03
*z*_H_2_S_/[mol mol^–1^]	0	0.03

a The data set comprises 19,361 state points (ca. 1.9 × 10^4^) evaluated for 100 training feeds sampled from the composition pool with sampling fractions (*f*_Dirichlet_, *f*_systematic_, *f*_industrial_) = (0.70, 0.20, 0.10). For each feed, state points were generated at temperatures from 200 to 300 K in steps of 5 K, with 10 pressures spaced equally between the dew-point and bubble-point pressures at each temperature. The impurity mole fractions have a minimum of zero because not every impurity is present in every mixture. Predictions outside the reported ranges constitute extrapolation, for which the reported error metrics do not apply.

### 2.5 Model Evaluation

To evaluate the quantitative performance of the regression ML models, we used the following metrics: the coefficient of determination (*R* ^2^), the root-mean-square error (RMSE), and the mean absolute error (MAE). These metrics are defined as

14 $$R^{2}=1-\frac{\sum_{i=1}^{N}{\left(y_{i}-{ŷ}_{i}\right)}^{2}}{\sum_{i=1}^{N}{\left(y_{i}-\mathrm{y̅}\right)}^{2}}$$

15 $$\mathrm{RMSE}=\sqrt{\frac{1}{N}\sum_{i=1}^{N}{\left(y_{i}-{ŷ}_{i}\right)}^{2}}$$

and

16 $$\mathrm{MAE}=\frac{1}{N}\sum_{i=1}^{N}\left|y_{i}-{ŷ}_{i}\right|$$

Here, *N* is the total number of data points, *i* indexes each data point, *y*_*i*_ is the reference value of the *i*th data point, *ŷ*_*i*_ is the corresponding predicted value from the ML model, and *y̅* is the mean of the reference values. The coefficient of determination (*R* ^2^) measures how well the predicted values from an ML model reproduce the variation in the reference data. Values of *R* ^2^ closer to unity indicate better predictive performance. The RMSE measures the average prediction error in the same physical units as the target property. Because the errors are squared before averaging, larger deviations are penalized more strongly. The MAE measures the average absolute prediction error and is less affected by large individual deviations than the RMSE. To compare prediction errors of target properties with different physical units and numerical ranges, the Normalized Root Mean Square Error (NRMSE) and Normalized Mean Absolute Error (NMAE) were also evaluated. These metrics are defined as

17 $$\mathrm{NRMSE}=\frac{\mathrm{RMSE}}{y_{\max}-y_{\min}}$$

18 $$\mathrm{NMAE}=\frac{\mathrm{MAE}}{y_{\max}-y_{\min}}$$

where *y*_max_ and *y*_min_ denote the maximum and minimum reference values of the corresponding target property, respectively. To ensure consistency, the same composition sets were used to compute *P*_bubble_, *P*_dew_, IFT, interfacial thickness, and the WSD residual using PCP-SAFT EoS + cDFT. The data sets were split into training, testing, and validation sets with a 0.70:0.15:0.15 ratio, and the same split was used for all ML models for each target property. To evaluate model robustness, a 5-fold Cross Validation (CV) was performed on the training set. In a 5-fold CV, the training set was divided into five subsets. For each CV run, four subsets were used to train the model, while the remaining subset was used for validation. This process was repeated five times so that each subset served once as the validation set. The resulting fold-wise metrics were then used to assess model robustness and identify possible overfitting or underfitting of the ML model.

## 3 Results and Discussion

This section evaluates the performance of machine learning (ML) models in predicting the *PT* phase envelope, including bubble- and dew-point pressures, and the interfacial properties (γ and *L*_10_^90^) of multicomponent CO_2_-rich mixtures. The reference phase envelope and interfacial properties were computed using the PCP-SAFT EoS + cDFT^57,59,60^ of the CO_2_-rich mixtures. Section 3.1 describes the composition pool of CO_2_-rich mixtures considered in this study. The effect of impurities on *P*_bubble_, *P*_dew_, γ, and *L*_10_^90^ is presented and discussed in Section 3.2. Section 3.3 evaluates the relationships between the target properties, *P*_bubble_, *P*_dew_, γ, and *L*_10_^90^, and the input features, *T*, *P*, and **z**, using the Spearman rank correlation^112^ and the Kolmogorov–Smirnov (KS)^113^ statistical metrics. Two supervised ML-based modeling strategies are considered in this work. For the full data-driven ML approach, Random Forest (RF),^90^ Support Vector Regression (SVR)^88^ with a Radial Basis Function (RBF) kernel, Gradient Boosting (XGB),^91,92^ and Tabular Prior-Data Fitted Network (TabPFN)^86^ were used. The performance of these models for *P*_bubble_, *P*_dew_, γ, and *L*_10_^90^ is presented in Section 3.4. In the full data-driven models, the thermodynamic state variables *T* and *P*, together with the feed composition vector **z**, were used as input features. The effects of the number of compositions and the sampling strategy on the predictive accuracy of the aforementioned ML models are evaluated in Section 3.5, using random, stratified, and active learning (AL) sampling methods. In addition to the full data-driven models, Section 3.6 discusses hybrid residual ML models for improving the interfacial tension (IFT) predicted by the semiempirical Winterfeld–Scriven–Davis (WSD) correlation.^41^ In this residual-learning approach, Symbolic Regression (SR),^97^ Gaussian Process Regression (GPR),^98^ and Stochastic Variational Gaussian Process Regression (SVGP)^87,100,104^ were trained on the difference between the WSD prediction and the PCP-SAFT EoS + cDFT^57,59,60^ reference IFT.

### 3.1 Composition Pool

In this work, multicomponent CO_2_-rich mixtures were generated with the constraint *z*_CO_2__ ≥ 95% to represent compositions relevant to CO_2_ transportation. The remaining impurity fraction, *z*_impurity_ ≤ 5%, was distributed between the impurity components using two sampling approaches: the Dirichlet distribution and systematic scaling. For each generated mixture, *z*_CO_2__ was then determined from the total impurity fraction to satisfy the composition closure condition. In addition, industrial feed compositions reported by Simonsen et al.^6^ were included to introduce realistic CO_2_ transport mixtures into the composition pool. We considered a pool of 1000 CO_2_-rich compositions. Figure 1a shows the CO_2_ mole fraction distribution for the three composition sources: Dirichlet distribution, systematic scaling, and industrial feeds. The individual *z*_CO_2__ compositions are plotted as markers, and the outliers are not shown separately. The middle 50% of the data, represented by the Interquartile Range (IQR), lies between approximately *z*_CO_2__ = 0.96 and 0.98 for the Dirichlet distribution, between *z*_CO_2__ = 0.95 and 0.97 for systematic scaling, and between *z*_CO_2__ = 0.96 and 1.00 for the industrial feeds. The CO_2_ mole fractions from the Dirichlet distribution and systematic scaling appear at fixed values, such as 0.96 and 0.97, because they were generated with two-decimal precision. This discretization was intentionally introduced so that the ML models could learn and interpolate thermodynamic properties for different feed-composition levels. In contrast, the industrial feed compositions have more continuous *z*_CO_2__ values because they come from reported feed data. Figure 1b shows the *z*_impurity_ distribution in a box plot with outliers (markers) for each impurity component. The majority of *z*_impurity_ is <0.03, since the other impurity component takes the remaining fraction, which is more likely in multicomponent systems. The distribution of *z*_impurity_ with respect to the composition source is presented in Figure 1c. The Dirichlet-sampling compositions mainly have their IQR in the low-impurity region, whereas the systematic scaling compositions have their IQR at higher impurity fractions, mostly for *z*_impurity_ > 0.01. For the industrial feeds, the major impurities, such as H_2_, Ar, N_2_, and CH_4_, have a wide IQR range compared with minor impurities such as O_2_, CO, and H_2_S. This combined strategy ensures that the impurity composition space is sampled efficiently while keeping the mixtures representative of CO_2_-rich transport streams. For the generated composition pool of size |P|=1000, Figure 1d presents the empirical distribution of mixture sizes, where the mixture size denotes the number of active components in each composition. The bars represent the percent-normalized relative frequency, defined as *f̂*_*i*_^*N*^ = (*n*_*i*_/*N*) × 100%, with ∑_*i*_ *f̂*_*i*_^*N*^ = 100%, where *n*_*i*_ is the number of compositions containing exactly *i* components and N=|P| . The solid red line represents the kernel density estimation (KDE) of the same sample, plotted on the same percent-normalized axis, *f̂* ^*N*^. Since the mixture size is integer-valued with unit bin width, the bar heights and KDE^114,115^ values are directly comparable. The distribution is approximately bell-shaped and centered between four and five components. Eight-component mixtures originate mainly from the industrial feed composition source, whereas the Dirichlet sampling, due to the impurity-weighting parameter **α**, favors lower-cardinality mixtures and rarely activates all seven impurities at the same time. Consequently, the shape of *f̂*_*i*_^*N*^ can be controlled through the sampling fractions (*f*_Dirichlet_, *f*_systematic_, *f*_industrial_) = (0.70, 0.20, 0.10), allowing the composition pool to be biased toward low, medium, or high cardinality mixture sets as required. The temperature, pressure, and composition ranges of the training data set generated from this pool are summarized in Table 1.

**Figure 1:**
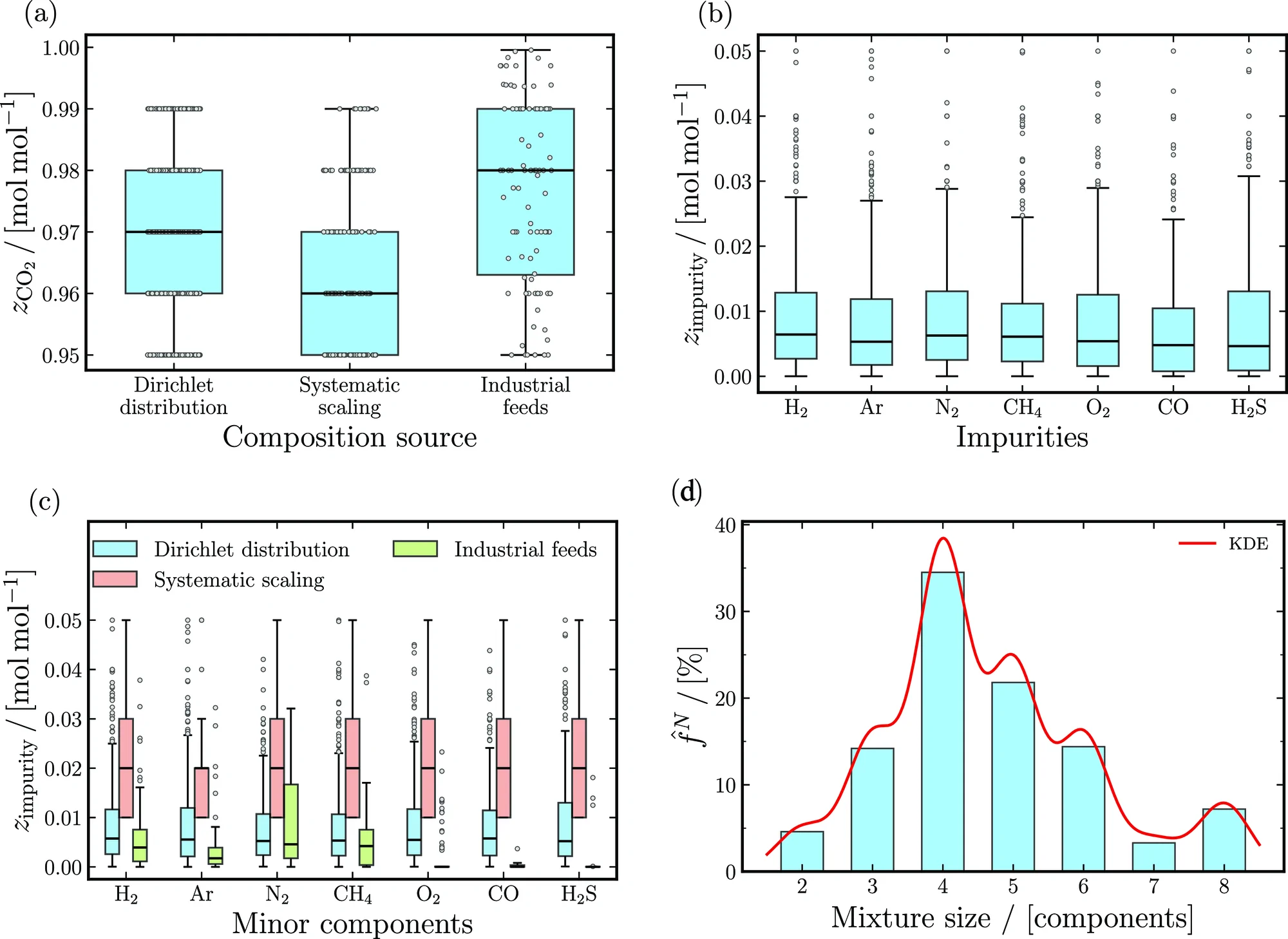
Composition-pool characteristics for the CO_2_-rich mixtures considered in this study for computing the phase envelope and interfacial properties. The composition pool P comprises *N* = 1000 compositions. (a) *z*_CO_2__ distribution grouped by each composition source. The markers represent the individual *z*_CO_2__ compositions from each feed obtained using the Dirichlet distribution,^105^ systematic scaling, and industrial feeds.^6^ (b) Box plots of the impurity mole fractions, *z*_impurity_, distribution for H_2_, Ar, N_2_, CH_4_, O_2_, CO, and H_2_S in the composition pool. (c) Comparison of *z*_impurity_ distributions separated by composition source, illustrating the different *z*_impurity_ ranges generated by the Dirichlet distribution,^105^ systematic scaling, and industrial feeds.^6^ (d) Normalized histogram of the mixture-size distribution. Bars give the percent-normalized relative frequency *f̂*_*i*_^*N*^, and the solid red line shows the kernel density estimation (KDE). The pool spans binary to eight-component mixtures, with the upper tail contributed by the industrial feed sampling fraction.

### 3.2 Effect of Impurities

Figure 2a–c presents the phase envelopes, interfacial tension (IFT), and interfacial thickness of selected CO_2_-rich mixtures computed using the PCP-SAFT EoS.^58,60,63^ The corresponding feed compositions are listed in Table 2. These five feeds were selected from the composition pool, P , to include different numbers of active components present in CO_2_-rich mixtures and to capture distinct *P*_bubble_ behavior. The phase envelopes in Figure 2a are compared with the pure-CO_2_ saturation line. The dew-point curves of the CO_2_-rich mixtures are nearly identical. Compared to the pure CO_2_ saturation line, noticeable differences near the critical region were identified. Compared to dew-point curves, bubble-point curves are much more sensitive to impurity type and its composition. For the investigated composition range, most impurities broaden the phase envelope of CO_2_-rich mixtures, while H_2_S tends to narrow it. For CO_2_ transportation, this broadening increases the range of temperatures and pressures over which vapor–liquid phase splitting can occur, so higher operating pressures may be required to maintain single-phase dense CO_2_ transport. This behavior is mainly related to the impurity critical temperature and volatility relative to CO_2_. Low critical temperature impurities such as H_2_, N_2_, Ar, and CH_4_ are more volatile than CO_2_ and increase the separation between the bubble- and dew-point curves. In contrast, H_2_S has a higher critical temperature and stronger condensability than CO_2_, and therefore behaves more like a heavy component. The broadening effect is especially clear for mixtures containing H_2_. For example, feed 1, a binary mixture without H_2_, has a narrow phase envelope, whereas feed 2, a quaternary mixture containing H_2_, has a much wider phase envelope. For feeds 2 and 4, *P*_bubble_ is higher at lower temperatures than at higher temperatures, which can be attributed to the negative Joule–Thomson behavior of H_2_.^116^ Figure 2b presents IFTs of the same selected mixtures computed at the *P*_bubble_ and *P*_dew_ curves and compared with pure CO_2_. The IFT values along the dew curves remain nearly identical to the pure-CO_2_ interfacial tension (IFT), almost independent of impurity type and its composition. IFT values at bubble curves show a stronger dependence on the feed composition and impurity. Compared with pure CO_2_, all selected CO_2_-rich mixtures have lower IFT on the bubble branch, with mean relative deviation (MRD) values ranging from ca. −14 to −26%. Feed 3 shows the lowest average deviation of ca. −14%, whereas feed 4 exhibits the largest average decrease of ca. −26%. The largest deviations are noticed at the high temperatures, around *T* = 298–300 K. Figure 2c presents the corresponding interfacial thickness, *L*_10_^90^, computed at the *P*_bubble_ and *P*_dew_ curves. Similar to the IFT, the *L*_10_^90^ values of the CO_2_-rich mixtures along the dew branch are almost identical to those of pure CO_2_, indicating that the impurities have only a minor effect on the dew branch interfacial properties. In sharp contrast, the mixtures have larger *L*_10_^90^ values than pure CO_2_ along the bubble branch, with MRD values ranging from approximately 10 to 20%. Feed 3 shows the smallest deviation, with an MRD of ca. 10%, whereas feed 4 exhibits the largest deviation, with an MRD of ca. 20%. However, the differences in *L*_10_^90^ between the CO_2_-rich mixtures listed in Table 2 are small compared with their deviations from pure CO_2_. Overall, impurities lower the IFT and increase the interfacial thickness, indicating a more diffuse interface than in pure CO_2_. From a transportation perspective, these changes can affect two-phase flow behavior once phase splitting occurs, because lower IFT facilitates phase dispersion and increases the interfacial surface area available for heat and mass transfer. The phase envelopes and interfacial properties were computed for several CO_2_-rich mixtures from the composition pool, P , similar to the five representative feeds listed in Table 2. The phase envelopes and interfacial properties data computed using the PCP-SAFT EoS + cDFT were then used for training ML models. For each feed composition, a two-phase contour map was generated to check the consistency of the PCP-SAFT EoS + cDFT computations, as illustrated in Figure 2d for feed 4. The contour map indicates that the IFT is strongly a function of *T*. At constant *T*, the IFT changes almost linearly with pressure, indicating that its pressure dependence along an isotherm can be approximated using a simple linear relation.

**Figure 2:**
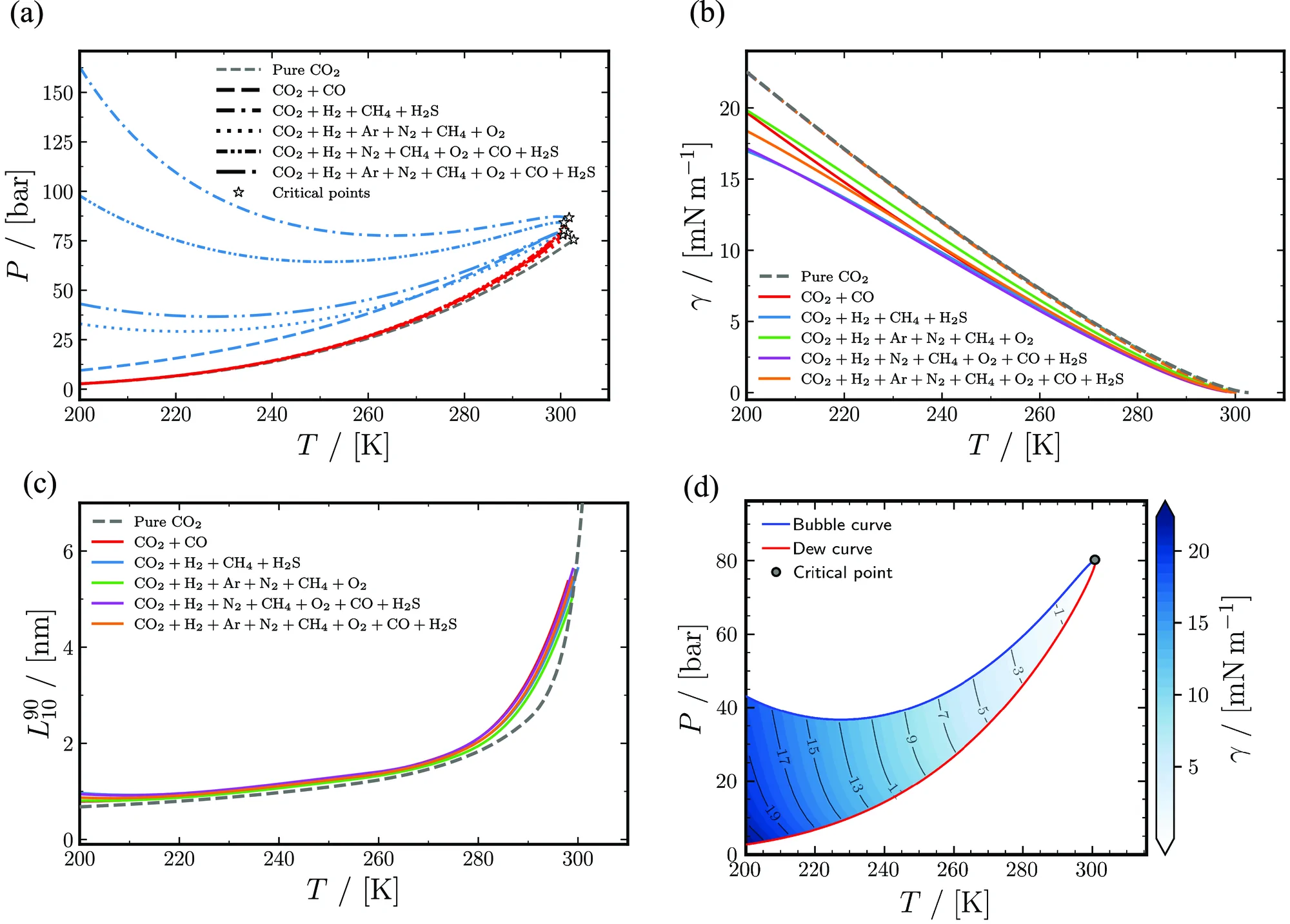
Sensitivity analysis of CO_2_-rich mixtures for the feed compositions listed in Table 2. (a) Bubble and dew-point pressure curves compared with pure CO_2_, with critical points. (b, c) Interfacial tension (IFT), γ, and interfacial thickness, *L*_10_^90^, at the bubble and dew equilibrium curves for the same CO_2_-rich mixtures and pure CO_2_ saturation line. (d) Two-phase envelope for the feed 4 listed in Table 2, including the bubble curve, dew curve, critical point, and the contour distribution of γ within the two-phase region. The phase envelope and interfacial properties were computed using the PCP-SAFT EoS coupled with cDFT using FeOs.^57–60^

**Table 2:** Mixture Compositions *z*_*i*_ in Mole Fractions Used for the Sensitivity Analysis Shown in Figure 2 ^a^

feed	*N*_c_	*z*_CO_2__	*z*_H_2__	*z*_Ar_	*z*_N_2__	*z*_CH_4__	*z*_O_2__	*z*_CO_	*z*_H_2_S_
1	2	0.9700						0.0300	
2	4	0.9500	0.0313			0.0039			0.0148
3	6	0.9800	0.0058	0.0091	0.0011	0.0029	0.0011		
4	7	0.9600	0.0200		0.0080	0.0040	0.0040	0.0020	0.0020
5	8	0.9700	0.0075	0.0040	0.0079	0.0099	3.79 × 10^–5^	6.99 × 10^–4^	3.90 × 10^–7^

a The feeds considered for the sensitivity analysis were selected from a composition pool of size 1000. The selected feeds span mixture sizes from 2 to 8 components.

### 3.3 Correlation Analysis of Features

To guide the input feature selection for the ML models, the feature-target relationships were evaluated using the Spearman correlation matrix^112^ and the Kolmogorov–Smirnov (KS) statistic,^113^ as shown in Figure 3. These analyses were performed on a composition pool with |P|=100, resulting in 19,361 thermodynamic state points generated using the PCP-SAFT EoS + cDFT framework.^57,59,60^ The Spearman correlation matrix in Figure 3a was used to identify monotonic nonlinear feature-target relationships and was preferred over Pearson correlation because the investigated phase-equilibrium and interfacial properties show nonlinear behavior, as illustrated in Figure 2a–c. However, a weak Spearman correlation does not necessarily imply feature irrelevance because nonmonotonic relationships can still give a ρ_s_ value close to zero. For this reason, the KS statistic in Figure 3b was used as a complementary direction-free relevance measure by comparing the target-property distributions between low- and high-feature-value groups. A high KS value with low ρ_s_ therefore indicates an important but potentially nonmonotonic feature–target relationship. This combination is useful here because all target properties show nonlinear behavior. For example, *P*_bubble_ can show a strongly nonlinear dependence on *T* for mixtures with higher H_2_ content, where the curve shape is affected by Joule–Thomson inversion behavior.^116^ The *P*_dew_ curves, which closely follow the pure-CO_2_ saturation line, can be related to an Antoine-type relation, ln *P* ∼ −*A*/*T* + *B*.^117^ Similarly, the IFT decreases nonlinearly with temperature, consistent with semiempirical correlations of the form γ = γ_0_(1 – *T*/*T*_c_)^*n*^,^118^ where *n* ≠ 1. The interfacial thickness also shows critical behavior and can be represented qualitatively as *L* ∼ (1 – *T*/*T*_c_)^−ν^,^119,120^ where the positive exponent ν causes *L* to increase sharply as *T* approaches *T*_c_. Overall, using both Spearman correlation and the KS statistic provides a more useful feature-relevance analysis than relying only on a linear correlation measure.

**Figure 3:**
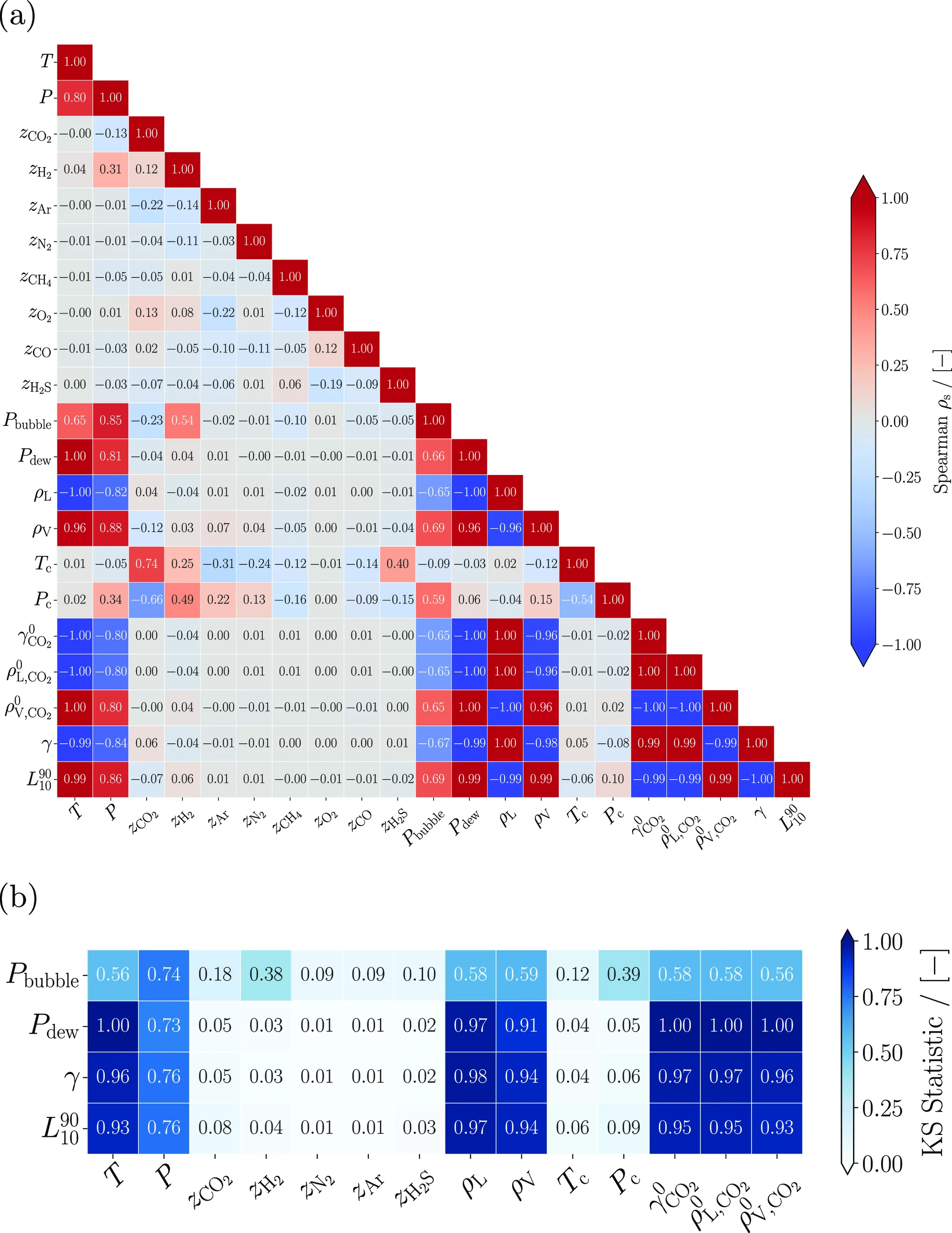
Feature-relevance analysis for the CO_2_-rich composition pool P=100. (a) Spearman rank-correlation matrix^112^ between the input features (*T*, *P*, **z**, ρ_L_, ρ_V_, *T*_c_, *P*_c_, γ_CO_2__^0^, ρ_L,CO_2__^0^, and ρ_V,CO_2__^0^) and the target properties *P*_bubble_, *P*_dew_, γ, and *L*_10_^90^. (b) Kolmogorov–Smirnov (KS) statistic^113^ between each input feature and target property. The Spearman coefficient, ρ_s_, identifies monotonic feature–target relationships, while the KS statistic quantifies distributional differences and provides an additional measure of feature sensitivity.

The KS analysis confirms the importance of the thermodynamic state variables, particularly *T* and *P*. Pressure shows relatively high KS values of ca. 0.7 for *P*_bubble_, *P*_dew_, γ, and *L*_10_^90^, while temperature reaches values close to one for *P*_dew_, γ, and *L*_10_^90^. The phase densities, ρ^L^ and ρ^V^, also show high KS values of ca. 0.9 for *P*_dew_, γ, and *L*_10_^90^. The Spearman matrix supports the same physical interpretation, where *P*_dew_, γ, and *L*_10_^90^ are strongly related to *T*, ρ^L^, and ρ^V^, whereas *P*_bubble_ is more sensitive to the feed composition, particularly *z*_H_2__ and *z*_CO_2__. Based on this feature-relevance analysis, the full data-driven models were trained using *T*, *P*, and the feed composition vector **z** as the final input features, defined as X={T,P,z}. Since the feed mole fractions satisfy ∑_*i*_ *z*_*i*_ = 1, only *N*_c_ – 1 composition variables are independent, giving a scalar feature-set cardinality of $\left|X_{\mathrm{scalar}}\right|=2+\left(N_{c}-1\right).$ The composition vector was kept explicit to ensure that the models remain direct functions of mixture composition and to allow the effects of individual impurities to be evaluated. The phase mole fraction vectors, **x** and **y**, pure-CO_2_ properties, and mixture critical properties were excluded because they largely repeat information already contained in *T*, *P*, and **z**. This avoids multicollinearity and unnecessary input dimensions while keeping the model physically interpretable and composition-dependent.

### 3.4 Machine Learning (ML) Model Performance and Outlier Analysis

The data set for the machine learning (ML) regression models was generated using the PCP-SAFT EoS + cDFT framework.^59,60,63^ To ensure broad coverage of the composition space, a pool of 100 feed compositions was constructed, resulting in approximately 1.9 × 10^4^ data points. The feed pools combined Dirichlet sampling, systematic scaling, and industrial feed compositions, with relative contributions of 0.7, 0.2, and 0.1, respectively. The predictive performance of the full data-driven models was evaluated using parity plots and prediction-error plots. The parity plots are presented in Figures S12–S15 of the Supporting Information for clarity, whereas the prediction-error plots are presented in Figure 4. These plots compare the predicted and reference values for *P*_bubble_, *P*_dew_, γ, and *L*_10_^90^ using Random Forest (RF), Support Vector Regression (SVR), Gradient Boosting (XGB), and Tabular Prior-data Fitted Network (TabPFN). The parity plots report the *R* ^2^ values, while Figure 4 reports the corresponding test and validation RMSE values using consistent axis ranges for each target property. The 5-fold Cross Validation (CV) metrics, including the mean and uncertainty of *R* ^2^, RMSE, and mean absolute error (MAE), are reported in Table 3. Although all models achieved high *R* ^2^ values in the single train-test-validation split, their error magnitudes, outlier behavior, and CV robustness varied widely. Outliers were identified using a 3-standard-deviation criterion (3σ), where a data point was flagged when its absolute residual exceeded 3 times the residual standard deviation. Since the 3σ threshold was computed separately for each model, a more accurate model can have a smaller threshold and still contain flagged outliers while maintaining better overall predictive performance.

**Figure 4:**
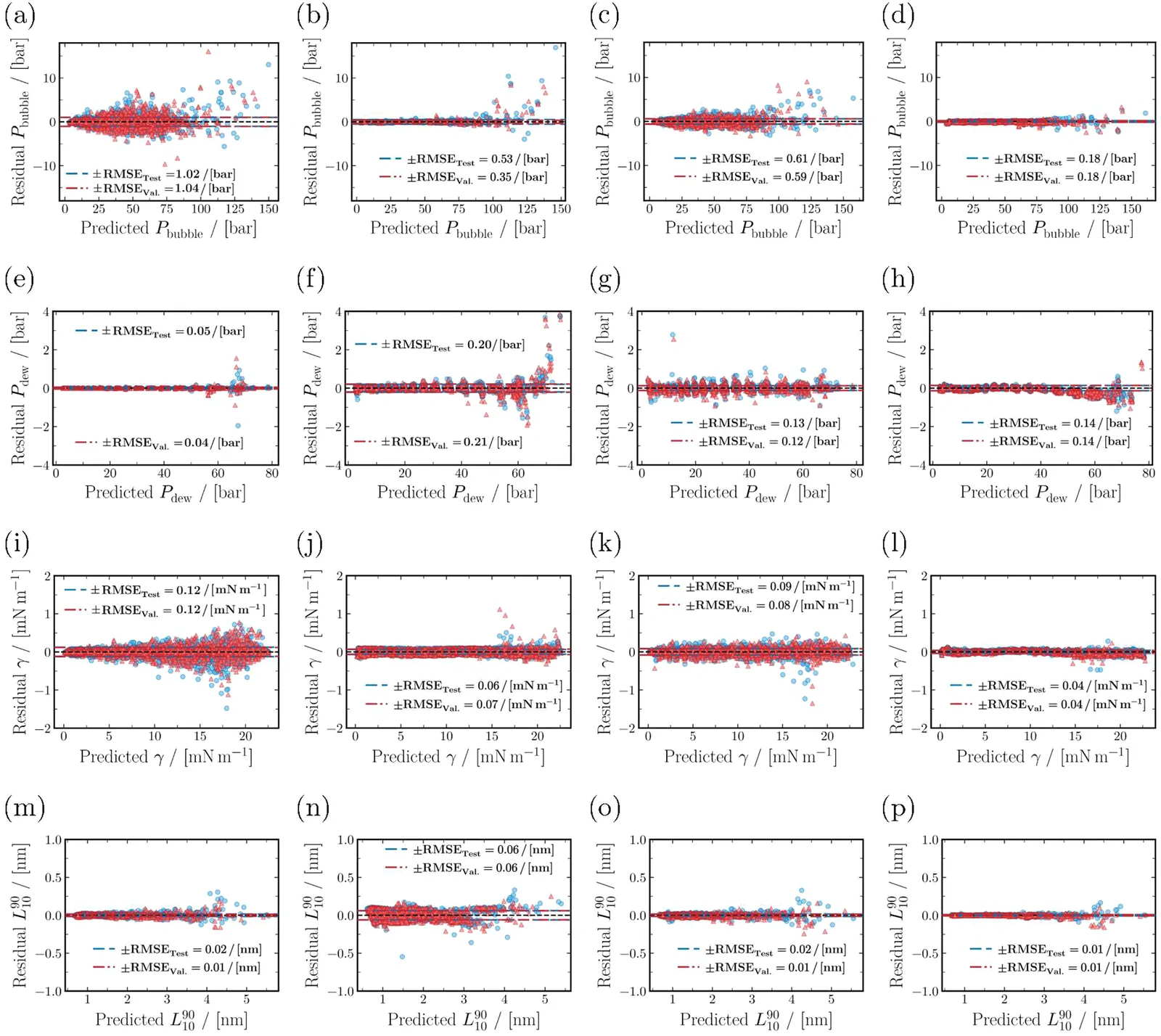
Residual analysis for the four target properties using the selected Machine Learning (ML) models. For each target property, the models are arranged from left to right as Random Forest (RF), Support Vector Regression (SVR), Gradient Boosting (XGB), and Tabular Prior-data Fitted Network (TabPFN). Panels (a–d), (e–h), (i–l), and (m–p) correspond to *P*_bubble_, *P*_dew_, γ, and interfacial thickness, respectively. The test and validation sets are presented separately to evaluate the residual distribution for both fitted and untrained data. The root-mean-square error (RMSE) values of each target property for the test and validation sets are reported in each subplot to quantify the corresponding prediction error. The test set is shown as blue circles with dashed ± RMSE bands, and the validation set is shown as red triangles with dash-dotted ± RMSE bands. The residuals are plotted against the predicted values to identify possible model bias, heteroscedasticity, and systematic deviations throughout the prediction range.^85,89–92^

**Table 3:** Five-Fold Cross Validation (CV) Performance of the Random Forest (RF), Support Vector Regression (SVR), Gradient Boosting (XGB), Tabular Prior-Data Fitted Network (TabPFN), and Random Forest (RF) + Tabular Prior-Data Fitted Network (TabPFN) Machine Learning (ML) Models for All Target Properties^a^

target property	ML model	*R* ^2^ (↑)	RMSE (↓)	MAE (↓)
*P*_bubble_	RF	0.9691 ± 0.0114	3.657 ± 0.849	2.456 ± 0.529
	SVR	0.9402 ± 0.0438	4.658 ± 2.368	2.436 ± 1.041
	XGB	0.9726 ± 0.0161	3.406 ± 1.181	2.300 ± 0.717
	TabPFN^b^	0.9828 ± 0.0062	2.732 ± 0.587	1.841 ± 0.254
	TabPFN^c^	0.9960 ± 0.0016	1.313 ± 0.302	0.766 ± 0.168
	RF + TabPFN^b^	0.9721 ± 0.0176	3.390 ± 1.308	2.034 ± 0.513
*P*_dew_	RF	0.9995 ± 0.0004	0.392 ± 0.139	0.120 ± 0.032
	SVR	0.9730 ± 0.0213	2.708 ± 1.314	1.336 ± 0.500
	XGB	0.9993 ± 0.0006	0.419 ± 0.219	0.170 ± 0.060
	TabPFN^b^	0.9997 ± 0.0002	0.298 ± 0.077	0.167 ± 0.010
	TabPFN^c^	0.9998 ± 0.0004	0.201 ± 0.185	0.041 ± 0.012
	RF + TabPFN^b^	0.9997 ± 0.0003	0.296 ± 0.138	0.124 ± 0.027
γ	RF	0.9991 ± 0.0003	0.186 ± 0.030	0.101 ± 0.011
	SVR	0.9765 ± 0.0184	0.856 ± 0.391	0.449 ± 0.168
	XGB	0.9992 ± 0.0004	0.174 ± 0.042	0.109 ± 0.024
	TabPFN^b^	0.9993 ± 0.0002	0.163 ± 0.017	0.122 ± 0.010
	TabPFN^c^	1.0000 ± 0.0000	0.040 ± 0.009	0.020 ± 0.004
	RF + TabPFN^b^	0.9993 ± 0.0004	0.157 ± 0.043	0.091 ± 0.019
*L*_10_^90^	RF	0.9964 ± 0.0046	0.0353 ± 0.0219	0.0116 ± 0.0019
	SVR	0.7786 ± 0.1583	0.2981 ± 0.1240	0.1726 ± 0.0526
	XGB	0.9965 ± 0.0048	0.0339 ± 0.0230	0.0118 ± 0.0015
	TabPFN^b^	0.9980 ± 0.0021	0.0272 ± 0.0143	0.0111 ± 0.0017
	TabPFN^c^	0.9982 ± 0.0010	0.0277 ± 0.0081	0.0074 ± 0.0026
	RF + TabPFN^b^	0.9970 ± 0.0038	0.0320 ± 0.0205	0.0096 ± 0.0022

a The reported values correspond to the mean ± standard deviation for the five validation folds. The arrows (↑ and ↓) indicate the desired direction of model performance. Higher values are better for *R* ^2^ (↑), whereas lower values are better for root-mean-square error (RMSE) and mean absolute error (MAE) (↓). The RMSE and MAE are reported in bar for *P*_bubble_ and *P*_dew_, mN m^–1^ for γ, and nm for the interfacial thickness (*L*_10_^90^). Superscripts indicate the TabPFN configuration, where *b* denotes no HyperParameter Optimization (HPO) and *c* denotes HPO.

b TabPFN without HPO.

c TabPFN with HPO.

#### 3.4.1 Bubble-Point Pressure (*P*_bubble_)

For *P*_bubble_, the prediction-error plots are shown in Figure 4a–d. The maximum residual is ca. 3 bar for TabPFN, ca. 9 bar for XGB, and ca. 16 bar for both RF and SVR. These large residuals occur mainly at low *T*, especially in the 200–210 K range, suggesting that this region of the thermodynamic space is more difficult to learn. This behavior is associated with mixtures containing higher H_2_ concentrations, where *P*_bubble_ changes strongly with the composition. The single-split test and validation RMSE values show that TabPFN achieves the lowest error, ca. 0.18 bar, followed by SVR, XGB, and RF, with values of ca. 0.53, 0.60, and 1.0 bar, respectively. However, the 5-fold CV metrics in Table 3 indicate that XGB generalizes better than SVR. Overall, TabPFN provides the best balance between accuracy and robustness, followed by XGB, SVR, and RF.

#### 3.4.2 Dew-Point Pressure (*P*_dew_)

For *P*_dew_, the prediction-error plots are shown in Figure 4e–h. Unlike *P*_bubble_, *P*_dew_ is less affected by impurities and remains close to pure CO_2_ saturation behavior. For the single train-test-validation split, RF yielded the lowest RMSE of ca. 0.05 bar, followed by TabPFN and XGB with ca. 0.1 bar and SVR with ca. 0.2 bar. However, TabPFN showed the best 5-fold RMSE, indicating more consistent fold-to-fold generalization. The maximum residuals were ca. 1.4 bar for TabPFN and ca. 2 bar for RF, ∼3 bar for XGB, and ∼4 bar for SVR. Most outliers were found in the near-critical region, mainly at *T* > 280 K. Overall, RF, TabPFN, and XGB perform similarly well for *P*_dew_, while SVR is the weakest model.

#### 3.4.3 Interfacial Tension (γ)

For γ, the prediction-error plots are shown in Figure 4i–l. Most outliers occur in the low-*T* region, particularly in the range of 200–210 K, similar to *P*_bubble_. The maximum residual is ca. 0.5 mN m^–1^ for TabPFN, ca. 1.1 mN m^–1^ for SVR, ca. 1.3 mN m^–1^ for XGB, and ca. 1.5 mN m^–1^ for RF. The single-split RMSE values also show that TabPFN performs best, followed by SVR, XGB, and RF. This suggests that the tree-based models struggle more in the low-*T* region compared to the transformer-based and SVM models. The 5-fold CV RMSE values in Table 3 show that the tree-based models generalize better than SVR, while TabPFN outperforms all models. Overall, TabPFN provides the best γ prediction, combining the lowest RMSE, the lowest worst-case residuals, and the best CV performance, while XGB serves as the closest conventional baseline.

#### 3.4.4 Interfacial Thickness (*L*_10_^90^)

For *L*_10_^90^, the prediction-error plots are shown in Figure 4m–p. Similar to *P*_dew_, *L*_10_^90^ is less sensitive to impurities, leading to low RMSE values for all investigated models. In contrast, SVR yielded the highest RMSE and the lowest *R* ^2^, with a 5-fold CV *R* ^2^ of ca. 0.77, indicating weak generalization. This may be attributed to the difficulty of the ϵ-insensitive loss in capturing the strongly nonlinear increase of *L*_10_^90^ near the critical point. TabPFN, XGB, and RF performed similarly on the single train-test-validation split, with RMSE values below ca. 0.02 nm, while TabPFN achieved the lowest test RMSE and the best 5-fold CV RMSE. Based on the outlier analysis, the maximum residuals for TabPFN, XGB, and RF were below ca. 0.3 nm, whereas for SVR, the maximum residual was about 0.5 nm. Overall, TabPFN is the best model for interfacial thickness prediction, with XGB and RF as close competitors.

#### 3.4.5 Selection and Evaluation of TabPFN-Based Models

Based on the prediction-error, outlier, and 5-fold CV analyses, TabPFN was selected as the most reliable full data-driven model for predicting the bubble- and dew-point pressures and interfacial properties of CO_2_-rich mixtures. For the other models, XGB showed the most consistent overall performance, RF performed well for impurity-insensitive properties, and SVR showed weaker fold-to-fold robustness during CV. Based on this result, different transformer-based TabPFN configurations^93^ were further investigated for *P*_bubble_, *P*_dew_, γ, and *L*_10_^90^. These configurations include the standard TabPFN model without HPO, TabPFN with HPO, and a hybrid RF + TabPFN model. In the hybrid model, RF partitions the input feature space, while TabPFN improves local predictions within the resulting partitions, thereby combining the structured partitioning ability of tree-based models with the predictive capability of the transformer-based TabPFN model.^93^ The same data set used for the RF, SVR, and XGB models was used for this comparison. Figure 5a–c compares the NRMSE, NMAE, and *R* ^2^ values, respectively, for all target properties. To enable direct comparison between properties with different units and magnitudes, the error metrics were normalized with respect to each target property, as shown in Figure 5a,b. All three TabPFN-based models resulted in low normalized errors for the four target properties. Overall, the tuned TabPFN model with HPO showed the best performance, giving the lowest NRMSE and NMAE for *P*_bubble_, *P*_dew_, and γ, as well as the highest *R* ^2^ values for all four properties. The improvement from tuning was most pronounced for γ, where the NRMSE decreased from ca. 0.7% for the default model to ca. 0.2%, corresponding to nearly a 4-fold reduction. For *P*_bubble_, the NMAE also decreased from ca. 1.2 to 0.5% after tuning. The default TabPFN and RF-TabPFN models showed similar performance, although both were slightly less accurate than the tuned model. The RF-TabPFN model yielded the largest errors for *P*_bubble_, with an NRMSE of ca. 2.1% and an NMAE of ca. 1.3%, but performed comparably to the other models for *P*_dew_ and γ. For *L*_10_^90^, the three models showed very similar results: the default TabPFN had the lowest NRMSE of ca. 0.6%, whereas the tuned model had the lowest NMAE of ca. 0.2% and the highest *R* ^2^ of 0.9982. These results show that HPO improved TabPFN for most target properties, with the strongest effect observed for γ. In contrast, the gain was limited for *L*_10_^90^, where all three models already performed similarly well. For CO_2_ transportation, these accuracies show that the tuned TabPFN model can rapidly screen the effects of impurities on phase boundaries and interfacial properties. This can help identify operating conditions near the two-phase region and estimate the compression pressure required to maintain single-phase dense CO_2_ transport without repeatedly performing expensive PCP-SAFT EoS + cDFT computations. The trained TabPFN surrogate for *P*_bubble_ and *P*_dew_ can therefore replace the rigorous flash calculations required in CFD and process simulations. The same holds for γ and *L*_10_^90^ in two-phase flow conditions. A surrogate is applicable only where it can be trusted. For a given temperature, pressure, and feed composition, TabPFN outputs a distribution of possible values for the target property rather than a single predicted value. Each prediction therefore carries a prediction interval (PI) at no additional cost.^85^ For example, of the Supporting Information reports the PI for the HPO-tuned *P*_bubble_ model of feed 2 in Table 2, together with the PCP-SAFT EoS reference values.

**Figure 5:**
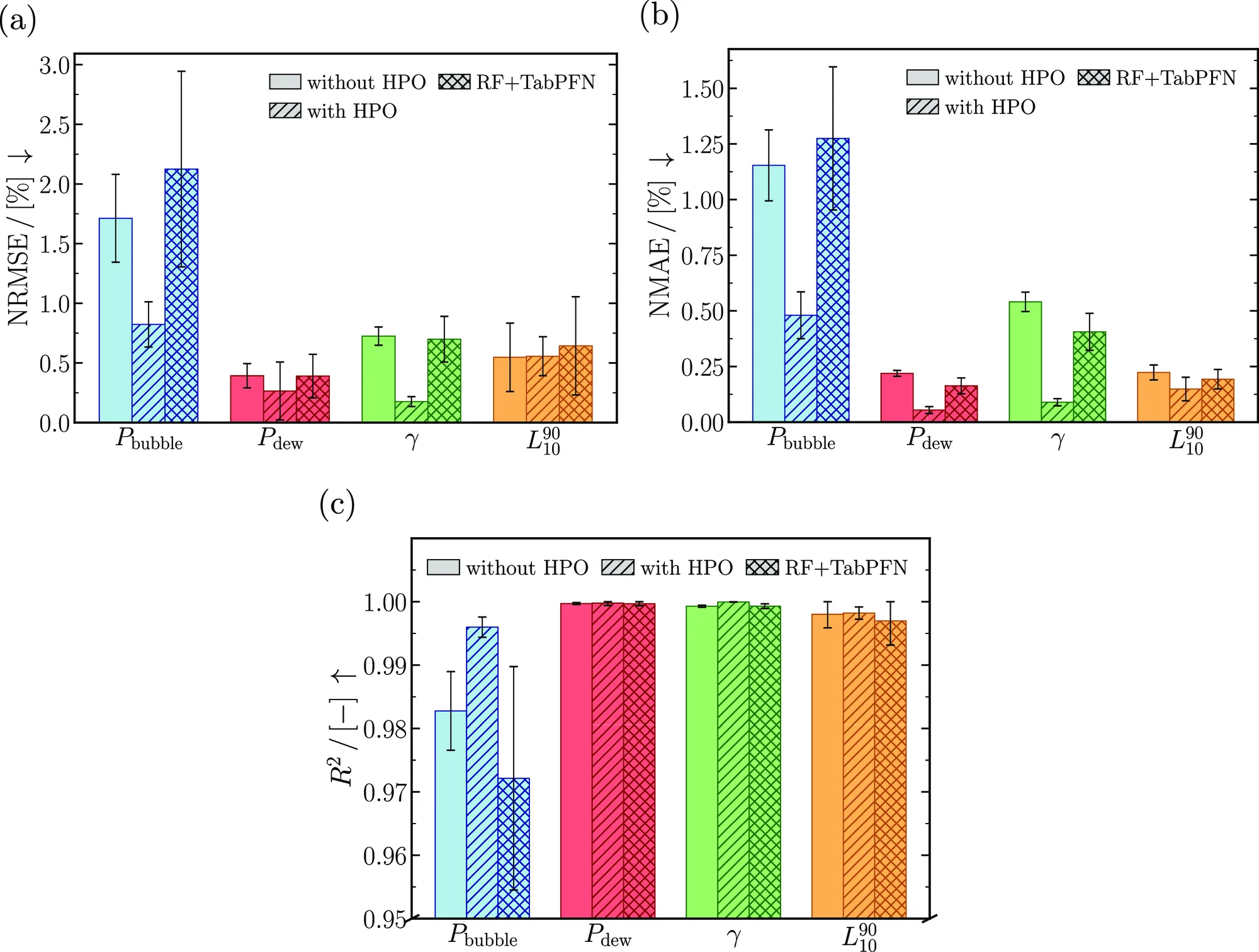
5-fold Cross Validation (CV) performance comparison of the Tabular Prior-data Fitted Network (TabPFN)-based models for the four target properties. (a–c) Comparison of the normalized root mean square error (NRMSE), normalized mean absolute error (NMAE), and *R* ^2^, respectively, for *P*_bubble_, *P*_dew_, γ, and *L*_10_^90^. The three model configurations correspond to TabPFN without HyperParameter Optimization (HPO), TabPFN with HPO, and the hybrid Random Forest (RF) + TabPFN models. The bar heights represent the mean values from the five CV folds, while the error bars indicate the corresponding standard deviations. The downward arrows in panels (a) and (b) indicate that lower error values are preferred, whereas the upward arrow in panel (c) indicates that higher *R* ^2^ values are preferred.^85,93^

#### 3.4.6 SHAP Analysis

To interpret the feature contributions for the best-performing ML models, SHAP analysis was performed for *P*_bubble_ and γ.^93,121^ These properties were selected because they are more sensitive to impurity type and concentration than *P*_dew_ and *L*_10_^90^, and are therefore more critical for CO_2_ transportation. The SHAP distributions for *P*_bubble_ predicted using XGB and TabPFN with HPO are shown in Figure 6a and b, respectively, and the corresponding distributions for γ are shown in Figure 6c and d. In each beeswarm plot, features are ranked by mean absolute SHAP value, and the sign indicates whether a feature increases or decreases the prediction relative to the model base value. For *P*_bubble_, both models identify *z*_H_2__ as the dominant feature, with high *z*_H_2__ values giving positive SHAP values and therefore increasing the predicted bubble-point pressure. This agrees with the expected behavior of H_2_ as a light impurity, which increases the pressure required to maintain the liquid phase. *T* and *P* are the next most important variables, while high *z*_CO_2__ values shift the prediction toward lower *P*_bubble_, consistent with more CO_2_-rich mixtures having lower bubble-point pressures. The remaining impurities have smaller contributions, although TabPFN shows some bidirectional trace-impurity effects, suggesting composition- and state-dependent interactions. For IFT, the dominant features shift from composition to thermodynamic state variables, with *T* ranked highest in both models, followed by *P*, while the composition features remain close to zero. High-temperature points mainly appear on the negative SHAP side, confirming that increasing temperature lowers the predicted interfacial tension. Overall, the agreement between XGB and TabPFN on the dominant features and their direction of influence supports the physical consistency of the learned trends, while the broader impurity response in TabPFN suggests that the transformer-based model captures more composition-dependent interactions than XGB.

**Figure 6:**
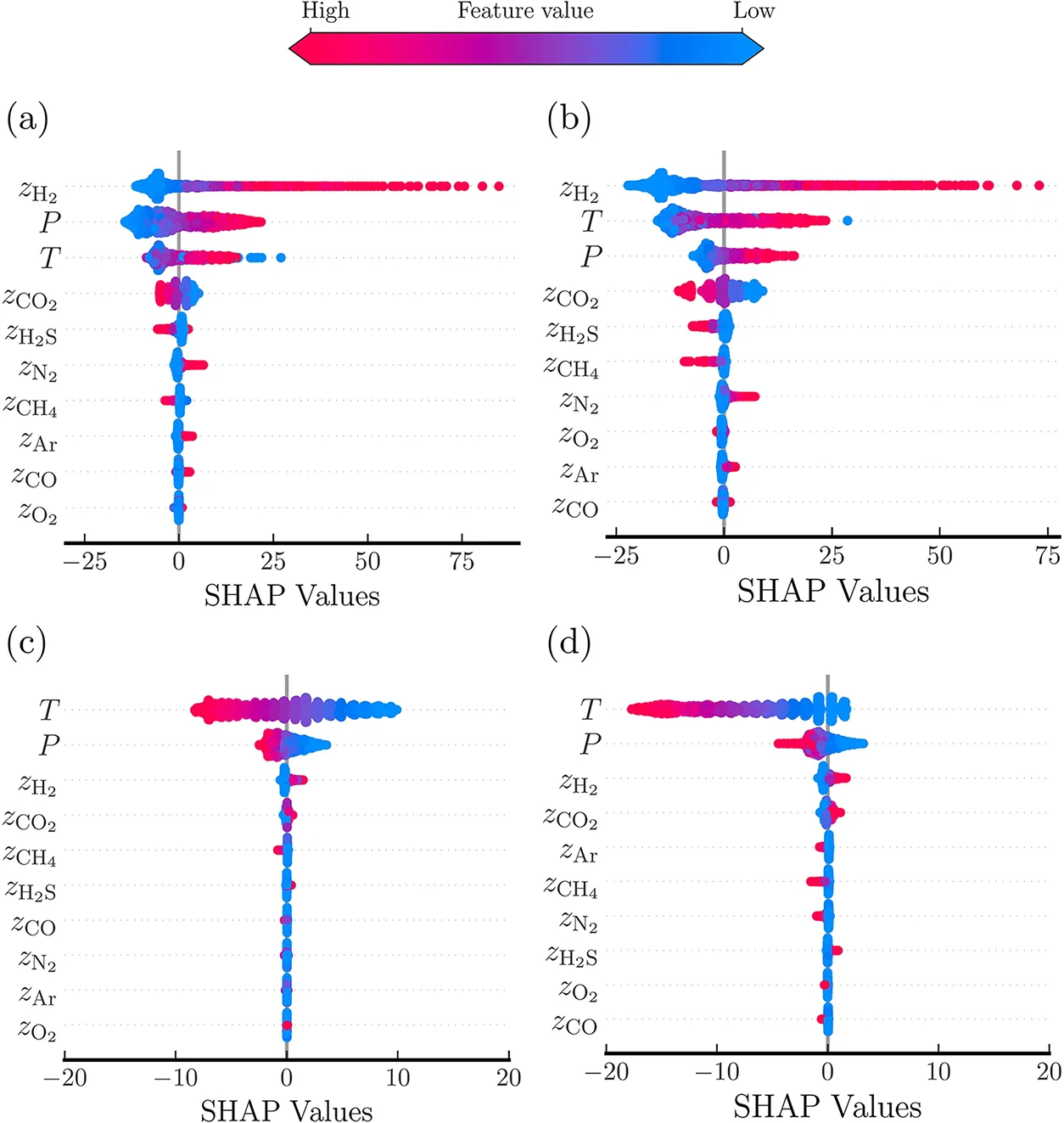
SHapley Additive exPlanation (SHAP) value distribution for the Gradient Boosting (XGB) and Tabular Prior-Data Fitted Network (TabPFN) models with HyperParameter Optimization (HPO). For *P*_bubble_, panel (a) corresponds to Gradient Boosting (XGB) and panel (b) corresponds to Tabular Prior-data Fitted Network (TabPFN). For γ, panel (c) corresponds to XGB and panel (d) corresponds to TabPFN. The features are ordered by overall importance, and each point represents the SHAP value of a feature for a single sample. The color scale indicates the feature value, with high values in red and low values in blue.^85,91,92^

To summarize, the model performance and interpretability analyses show that TabPFN provides the most reliable full data-driven model for predicting phase-equilibrium and interfacial properties of CO_2_-rich mixtures. Compared with the tested models, the HPO-tuned TabPFN model achieves the best overall performance, especially for *P*_bubble_ and γ. These two properties are particularly important because they are more sensitive to impurity type and concentration and therefore cannot be easily generalized for different CO_2_-rich mixture compositions. The SHAP analysis further confirms that *P*_bubble_ predictions are governed mainly by the *z*_H_2__ concentration, whereas γ predictions are governed primarily by *T* and *P*, supporting the physical consistency of the learned trends. Since this study aims not only to screen suitable ML models but also to evaluate the number of feed compositions required to achieve a target prediction accuracy, a composition-space sampling analysis was performed using the two best-performing models, TabPFN and XGB, for *P*_bubble_ and γ, as described in the next section.

### 3.5 Composition Space Sampling

To evaluate the number of feed compositions required to train full data-driven ML models to a given accuracy, a composition-space sampling analysis was performed. The feed compositions were sampled from the composition pool P , which represents CO_2_ transportation-relevant mixtures. The pool contained 1000 feed compositions, and the pool characteristics are shown in Figure 1. The three sampling methods investigated in this study were random sampling, stratified sampling, and AL sampling with four feed composition sizes (*N*_train_) of 25, 50, 75, and 100. The minimum size was set to 25 because the stratified sampling method used 25 strata. For each selected feed composition, the target properties were generated using the PCP-SAFT EoS + cDFT framework.^59,60,63^ For each sampling method and *N*_train_ size, evaluation was performed over 20 independent trials. In each trial, a different random seed was used for both *N*_train_ composition selection and 5-fold CV partitioning so that the reported metrics reflect the effect of the selected mixture compositions rather than a particular split. For TabPFN, the default model without HPO was used, because performing HPO for 20 independent trials and 4 *N*_train_ sizes was computationally demanding. Figure 7a,b shows the 5-fold CV RMSE of *P*_bubble_, averaged over the 20 trials for the TabPFN and XGB models, respectively. For both models, the RMSE decreases as the number of feed compositions increases, with the largest improvement observed when increasing the training size from 25 to 50 compositions. For AL sampling, the TabPFN RMSE decreases from 6.8 to 4.1 bar, while the MAE decreases from 3.6 to 2.0 bar, as shown in Figure 7c, corresponding to an error reduction of about 40%. For XGB, the RMSE decreases from 6.9 to 5.0 bar, while the MAE decreases from 3.5 to 2.4 bar, as shown in Figure 7d, corresponding to an error reduction of about 28%. For *N*_train_ larger than 50, the error continues to decrease, but the improvement becomes smaller, reaching RMSE values of 2.3 bar for TabPFN and 3.8 bar for XGB at 100 compositions. This indicates that the models are approaching a performance plateau where further training compositions yield diminishing improvements. The same trend is observed in the CV MAE results shown in Figure 7c,d for TabPFN and XGB, respectively. Compared with random and stratified sampling, AL achieves the lowest RMSE and MAE for all *N*_train_ sizes, indicating that adaptive selection of informative feed compositions is more data-efficient than nonadaptive sampling. Random and stratified sampling show similar performance at larger training sizes, namely, *N*_train_ = 50, 75, and 100, but at the smallest size, *N*_train_ = 25, random sampling performs better than stratified sampling, with RMSE values of 7.5 and 8.8 bar, respectively, for TabPFN. For this reason, random sampling is preferred as the nonadaptive baseline because it matches stratified sampling at larger training sizes while outperforming it in the low-data regime. Overall, these results indicate that approximately 50 feed compositions provide a favorable balance between the accuracy and computational cost for predicting *P*_bubble_. Moreover, AL provides the most data-efficient sampling strategy by reducing the number of feed compositions required to reach a given level of prediction accuracy.

**Figure 7:**
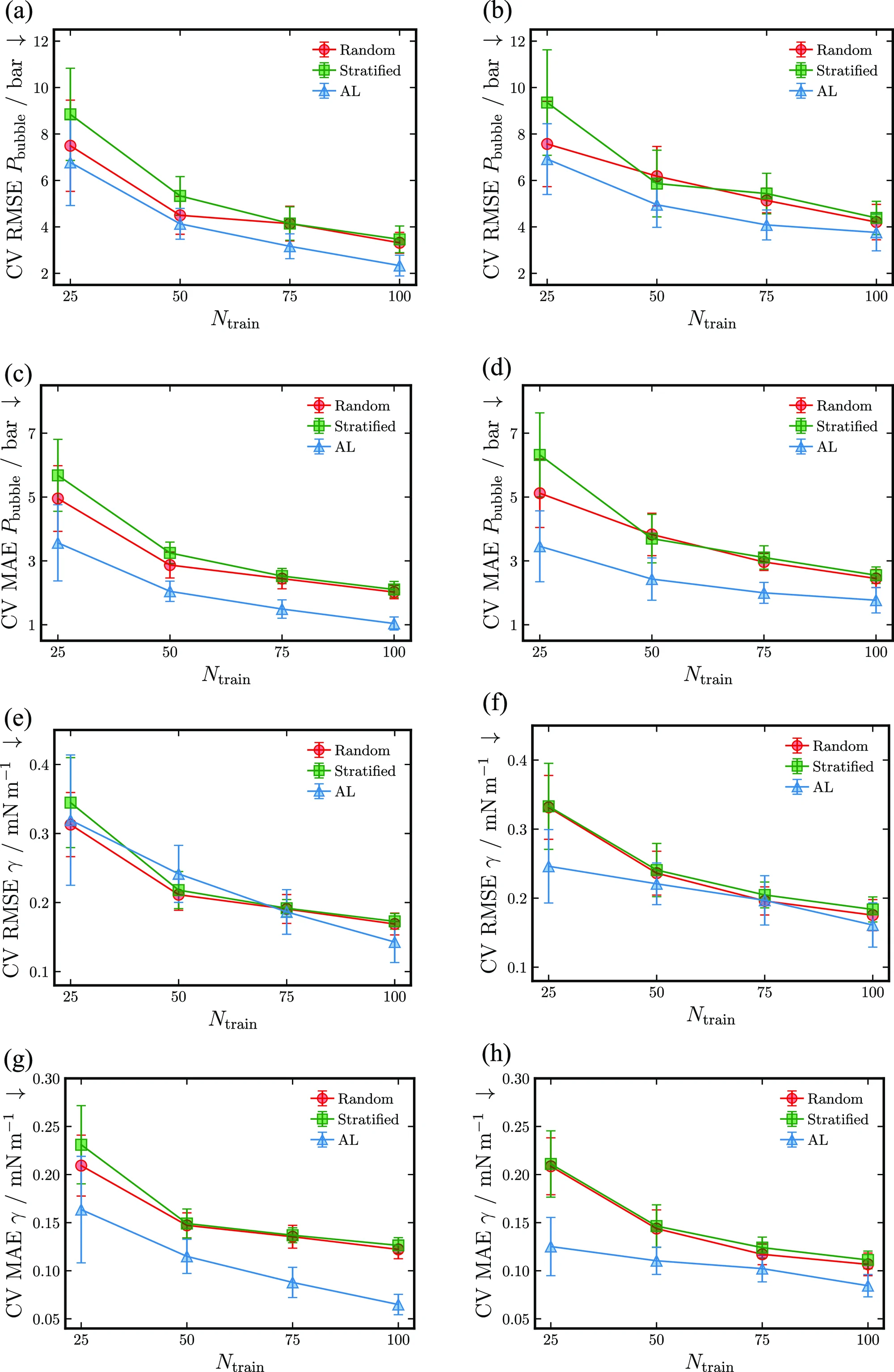
Comparison of random, stratified, and active learning (AL) sampling strategies for different training composition sizes, *N*_train_. For *P*_bubble_, panels (a) and (b) correspond to root-mean-square error (RMSE) for Tabular Prior-data Fitted Network (TabPFN) and Gradient Boosting (XGB), respectively, while panels (c) and (d) correspond to mean absolute error (MAE) for TabPFN and XGB, respectively. For γ, panels (e) and (f) correspond to RMSE and (g, h) to MAE, for TabPFN and XGB, respectively. The RMSE and MAE values were obtained from 5-fold Cross Validation (CV). The markers represent the mean values over 20 independent trials, and the error bars represent the corresponding 95% confidence intervals. Low RMSE and MAE values indicate better predictive performance, as indicated by the arrow ↓.^85,91,92,109^

Figure 7e and f shows the 5-fold CV RMSE of γ for the TabPFN and XGB models, respectively. The values are averaged over the 20 independent trials and are shown as a function of *N*_train_. The corresponding 5-fold CV MAE values are shown in Figure 7g,h. For TabPFN, all sampling strategies show similar RMSE values for the investigated *N*_train_ sizes, indicating that the model is less sensitive to the sampling strategy for γ than for *P*_bubble_. Similar to *P*_bubble_, the largest improvement is observed when *N*_train_ is increased from 25 to 50. For AL sampling, the TabPFN RMSE decreases from 0.32 to 0.24 mN m^–1^, while the MAE decreases from 0.16 to 0.12 mN m^–1^. For XGB, the RMSE decreases from 0.25 to 0.22 mN m^–1^, while the MAE decreases from 0.13 to 0.11 mN m^–1^. When the *N*_train_ is increased to 100, the RMSE values of 0.14 mN m^–1^ for TabPFN and 0.16 mN m^–1^ for XGB are noticed, showing that the improvement becomes smaller beyond 50 compositions. Overall, the γ predictions are highly accurate, with errors below 0.5 mN m^–1^ for all sampling methods and *N*_train_. For XGB, AL sampling produces the lowest RMSE for all *N*_train_, whereas random and stratified sampling perform similarly. The MAE results show the benefit of AL more clearly, as AL produces the lowest mean error for both TabPFN and XGB for the investigated feed composition sizes. This indicates that AL selects more informative feed compositions for predicting γ, although for TabPFN, the RMSE values of AL and random sampling remain comparable at *N*_train_ < 100. Random and stratified sampling again show similar performance, with random sampling slightly better at *N*_train_ = 25, making it the preferred nonadaptive baseline. Together with the *P*_bubble_ results, these findings indicate that approximately 50 feed compositions provide a good balance between accuracy and computational cost for training full data-driven ML models, while AL offers the most data-efficient sampling strategy, especially when the target is to reduce the number of training compositions required to reach a given accuracy. Based on these results, we recommend GPR-based AL as the sampling strategy for extending the present data set, for example, to additional impurity types.

### 3.6 Hybrid Machine Learning (ML) Model for Interfacial Tension (IFT)

We further evaluated γ using a hybrid residual-learning approach, in which the simple semiempirical WSD model^41^ was combined with an ML model trained to learn the systematic residuals of the WSD predictions. This approach tests whether incorporating physical prior knowledge improves the predictive accuracy of IFT. The residual, Δγ, was defined as the difference between the IFT computed using the PCP-SAFT EoS + cDFT framework^57,59,60^ and that predicted by the WSD correlation, i.e., Δγ = γ_cDFT_ – γ_wsd_. The residuals were computed for the same 100 feed compositions constructed in Section 3.4. Figure 8a shows the contour map of Δγ within the two-phase region, bounded by the dew- and bubble-point curves, for feed 4 listed in Table 2. The residuals are mostly negative, indicating that the WSD correlation overpredicts IFT compared to the PCP-SAFT EoS + cDFT framework.^57,59,60^ This overprediction is more pronounced at lower temperatures and higher pressures, where the magnitude of Δγ increases. This behavior can be attributed to the larger density difference between the coexisting phases at these conditions. At lower temperatures, the liquid phase becomes more cohesive and denser, whereas volatile impurities preferentially partition into the vapor phase. As a result, the WSD model tends to overestimate the cohesive contribution of the subcritical mixture and does not fully capture the impurity-induced reduction in IFT. The residual distribution also shows a smooth, structured pattern within the two-phase region rather than random scatter. In particular, Δγ remains close to zero near the critical point and becomes increasingly negative toward the low-temperature and high-pressure region. This indicates that the error of the WSD correlation has a systematic dependence on the thermodynamic state. Similar contour maps were generated for all 100 feed compositions, and the residuals were found to range from 0 to ca. −5 mN m^–1^, as shown in Figure 8b. The pressure-colored scatter plot shows that the largest negative residuals are mainly associated with high-pressure states, particularly above ca. 75 bar. This trend is also evident in Figure 8c, where Δγ is plotted as a function of both *T* and *P*, confirming that the largest deviations are concentrated in the low-temperature and high-pressure region of the two-phase envelope. The structured residual field shown in Figure 8c supports the residual-learning strategy because the ML model only needs to learn a smooth state-dependent (*T* and *P*) correction to the semiempirical WSD prediction rather than learning the complete IFT response. For this purpose, we used Symbolic Regression (SR), Gaussian Process Regression (GPR), and Stochastic Variational Gaussian Process Regression (SVGP).

**Figure 8:**
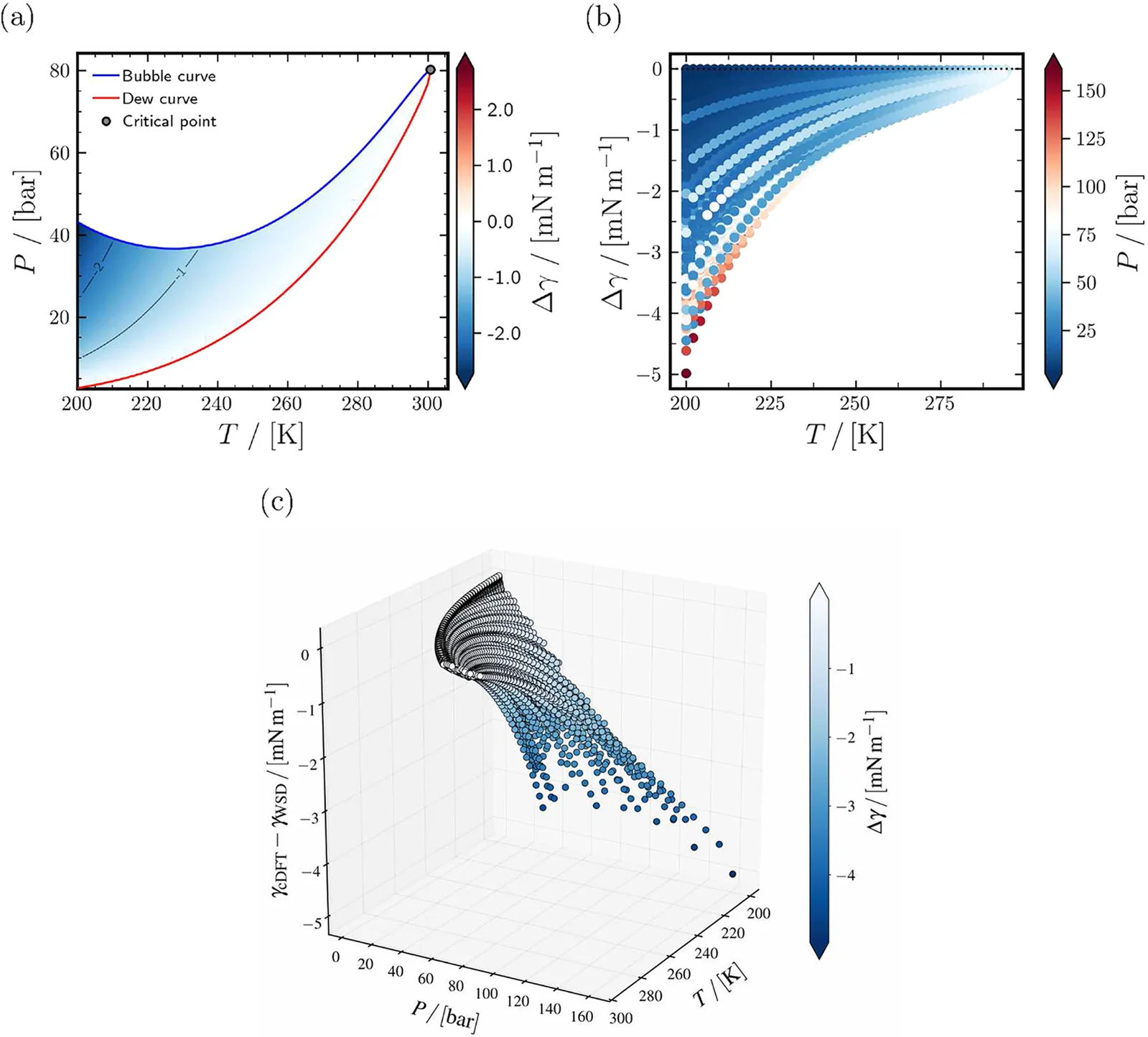
Residual analysis of the Winterfeld–Scriven–Davis (WSD) model for interfacial tension (IFT), using the Perturbed-Chain Polar Statistical Associating Fluid Theory (PCP-SAFT) Equation of State (EoS) + classical Density Functional Theory (cDFT) results as reference. (a) *PT* two-phase envelope with the bubble curve, dew curve, critical point, and contour distribution of the IFT residual, Δγ. (b) Variation of Δγ with temperature inside the two-phase region, with the color scale indicating pressure. (c) Three-dimensional residual surface of Δγ as a function of temperature and pressure. The residual maps show the deviation of the WSD model from the PCP-SAFT + cDFT reference calculations throughout the two-phase region.^41^

#### 3.6.1 Physically Interpretable Symbolic Regression (SR) Correction for the Winterfeld–Scriven–Davis (WSD) Semiempirical Correlation

Figure 9a and b shows the parity plot and prediction-error analysis for the Symbolic Regression (SR)-based hybrid model for IFT, in which the baseline WSD semiempirical correlation was combined with a learned correction for the relative residual. Instead of training the SR model directly on the dimensional residual Δγ (mN m^–1^), the target was defined as the dimensionless relative residual ϵ_γ_ = (γ_cDFT_ – γ_base_)/γ_base_, where γ_cDFT_ is the PCP-SAFT EoS + cDFT reference value and γ_base_ is the baseline WSD prediction. The predicted residual was then mapped back to the IFT using γ̂ = γ_base_(1 + ϵ_γ_). Hence, the parity plot and prediction-error analysis for the reconstructed γ̂_cDFT_ are shown in Figure 9a and b, respectively. The input feature set F for training the ϵ_γ_ was constructed from two groups of physically motivated variables. The first group contained reduced state and interfacial quantities, including *T*_r_^*^ = *T*/*T*_c_, 1 – *T*_r_^*^, *P*_r_^*^ = *P*/*P*_c_, Π* = *P*/*P*_sat_^0^, Δρ = ρ_l_ – ρ_v_, Δρ* = (ρ_l_ – ρ_v_)/(ρ_l_^0^ – ρ_v_^0^), and γ_r_^*^ = γ_base_/γ_CO_2__^0^. The dimensionless input features used are represented by *. The *T*_c_ and *P*_c_ of pure CO_2_ are used in *T*_r_^*^ and *P*_r_^*^. The second group contained composition-based impurity aggregates such as the liquid-, vapor-, and feed-phase impurity sums, which are defined as *x*_imp_ = ∑_*i*≠CO_2__ *x*_*i*_, *y*_imp_ = ∑_*i*≠CO_2__ *y*_*i*_, and *z*_imp_ = ∑_*i*≠CO_2__ *z*_*i*_, respectively. In addition, the second group was also combined with the phase-partitioning difference Δ_imp_ = *x*_imp_ – *y*_imp_ and the impurity partition coefficient $l_{K}=\ln\left[\left(y_{\mathrm{imp}}+\xi \right)/\left(x_{\mathrm{imp}}+\xi \right)\right],$ with ξ = 10^–12^ to avoid division by zero. Combining both groups resulted in a 12-feature set, i.e., |F|=12, which provides the SR model with physically informed variables to correct the limitations of the baseline WSD model for CO_2_-rich mixtures with impurities. Because the baseline WSD model already contains the dominant temperature- and pressure-dependent contribution to IFT, the SR model was also tested using only the composition-based impurity aggregates $\left\{z_{\mathrm{imp}},x_{\mathrm{imp}},y_{\mathrm{imp}},\Delta_{\mathrm{imp}},l_{K}\right\}$ to examine whether the missing correction could be represented only through impurity partitioning features. For this purpose, the SR search was performed over 200 iterations using simple unary and binary operators, yielding several candidate equations with varying complexities and corresponding RMSE values. This reduced feature set SR search recovered the previously suggested correction ϵ̂_γ_ ≈ −2.56*x*_imp_,^25^ where the prefactor 2.56 is an additional empirical constant obtained compared to the previous correction factor suggested by us.^25^ This shows that the correction is approximately proportional to the liquid-phase impurity fraction, although it remains less accurate. From the full 12-feature set, the selected correction from the Pareto frontier was

19 $${\mathrm{\epsilon ̂}}_{\gamma }\approx \ln\left(\Delta \rho^{*}\right)/\left[\sqrt{P_{r}^{*}}+\left(\gamma_{r}^{*}-\Delta \rho^{*}\right)\right]$$

which achieved a test *R* ^2^ of 0.94 in residual space. After reconstruction to IFT, this correction yielded a *R* ^2^ ≈ 1 (as shown in Figure 9a), with an RMSE of 0.14 mN m^–1^ and an MAE of 0.074 mN m^–1^. In comparison, the correction (ϵ̂_γ_ ≈ −2.56*x*_imp_) obtained using only the composition-based feature set resulted in a residual-space test *R* ^2^ of 0.618 and a reconstructed-IFT RMSE of 0.29 mN m^–1^. The robustness of both the full 12-feature and composition-only corrections was further confirmed by 5-fold CV. The corresponding scores are summarized in Table 4.

**Figure 9:**
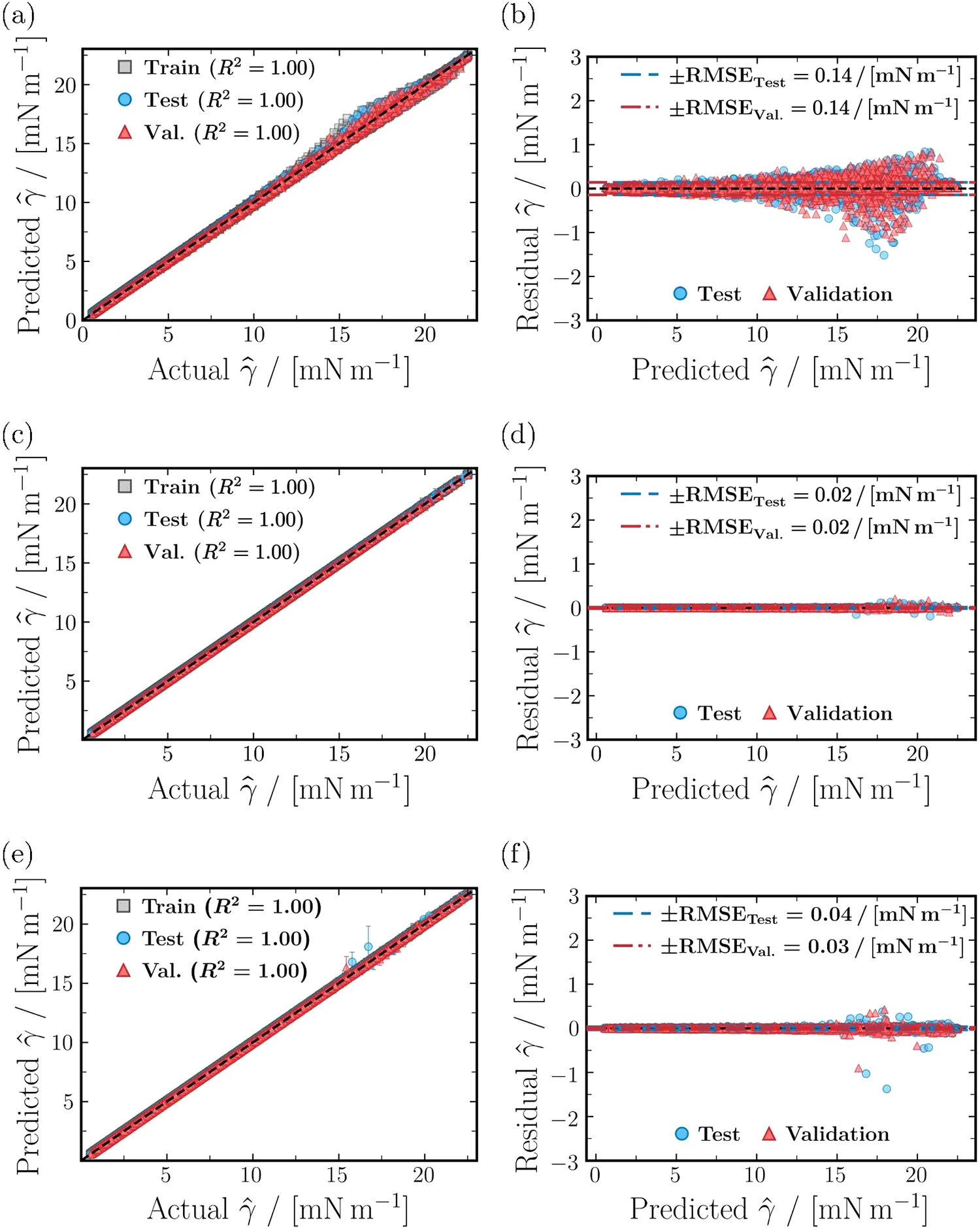
Parity and prediction-error analysis for the hybrid interfacial tension (IFT) residual models applied to γ. (a, c, e) Comparison of the predicted values with the reference values and report the corresponding *R* ^2^ values. The right panels (b, d, f) present the residuals as a function of the predicted values with the corresponding root-mean-square error (RMSE) values for the test and validation data sets. (a, b), (c, d), and (e, f) correspond to Symbolic Regression (SR), Gaussian Process Regression (GPR), and Stochastic Variational Gaussian Process Regression (SVGP), respectively. For GPR and SVGP, the parity plots include the model-predicted uncertainties.^87,97–100^

**Table 4:** Five-Fold Cross Validation (CV) Performance of the Hybrid ML Residual Model, Evaluated for the Reconstructed IFT (γ̂): Symbolic Regression (SR), Gaussian Process Regression (GPR), and Stochastic Variational Gaussian Process Regression (SVGP)^a^,^b^

model	*R* ^2^ (↑)	RMSE/[mN m^–1^] (↓)	MAE/[mN m^–1^] (↓)
SR $\left(F_{\mathrm{Comp}.}\right)$	0.9983 ± 0.0002	0.2475 ± 0.0110	0.1434 ± 0.0073
SR (F)	0.9996 ± 0.0000	0.1130 ± 0.0073	0.0603 ± 0.0031
GPR	0.9999 ± 0.0001^b^	0.0379 ± 0.0262	0.0051 ± 0.0011
SVGP	1.0000 ± 0.0000^b^	0.0233 ± 0.0030	0.0119 ± 0.0014

a The reported *R* ^2^, root-mean-square error (RMSE), and mean absolute error (MAE) values are the mean ± standard deviation over the five folds. The arrows indicate the desired direction of model performance. The SR model was trained on the dimensionless relative residual ϵ_γ_, whereas the GPR and SVGP models were trained on the dimensional residual Δγ. For the GPR and SVGP, the reconstructed IFT RMSE and MAE are therefore identical to the residual-space values. The two SR entries correspond to the composition feature set $F_{\mathrm{Comp}.}=\left\{z_{\mathrm{imp}},x_{\mathrm{imp}},y_{\mathrm{imp}},\Delta_{\mathrm{imp}},l_{K}\right\}$ and the full physics-informed feature set $F=F_{\mathrm{Comp}.}+\{T_{r}^{*},1-T_{r}^{*},P_{r}^{*},\Pi^{*},\Delta \rho ,\Delta \rho^{*},\gamma_{r}^{*}\}.$

b The reconstructed-IFT *R* ^2^ is obtained per fold as 1 – RMSE^2^/Var­(γ_cDFT_).

Although both corrections were obtained from the SR search, the selected input features are physically motivated and allow a clear interpretation. The Pareto fronts obtained from the physics-informed and composition-only feature sets are shown in Tables S9 and S10, respectively. The Pareto-frontier equations shown in Table S9 are expressed in terms of the density difference (Δρ) between the liquid and vapor phases. Similarly, the equations listed in Table S10 are expressed in terms of the total liquid mole fraction of impurities (*x*_imp_). The *x*_imp_-based equations have a direct physical analogy to surfactant behavior because the impurities decrease the IFT of the CO_2_-rich mixture much like a surfactant, consistent with our previous findings.^25^ The negative prefactor (−2.56) reflects this monotonic reduction in IFT with increasing liquid-phase impurity content. However, the WSD residual is not governed by composition alone. It also varies with temperature and pressure, with the largest deviations observed at low temperature and high pressure, as shown in Figure 8. The physics-informed Pareto-frontier equations account for these dependencies, even though the baseline WSD model already contains strong *T*- and *P*-dependent terms. For the chosen correction (eq 19), the numerator ln­(Δρ*) reflects the sharpness of the interface. A large density difference corresponds to a steep density gradient and a highly cohesive, sharp interface. In sharp contrast, a smaller density difference corresponds to a more diffuse interface, with Δρ* vanishing smoothly toward the critical point. The impurity effect enters implicitly through the bulk densities because impurities lower both the liquid and vapor densities relative to pure CO_2_. The denominator, $\sqrt{P_{r}^{*}}+\left(\gamma_{r}^{*}-\Delta \rho^{*}\right)$ , combines a pressure-dependent term with a mismatch between the reduced baseline IFT and the reduced density difference. The term $\sqrt{P_{r}^{*}}$ increases with pressure and therefore damps the correction at higher pressures. The difference (γ_r_^*^ – Δρ*) accounts for deviations between the interfacial response and the bulk density contrast, which can arise from impurity partitioning and interfacial enrichment. Together, these terms make the denominator a state-dependent damping factor. The equations, feature-engineering details, and SR configuration are provided in Section S5.2 and Tables S8–S12 of the Supporting Information.

#### 3.6.2 GP-Based Models

Figure 9c and d shows the parity plot and prediction-error analysis for the GPR hybrid model, respectively. GPR is well-suited for learning the residual function shown in Figure 8c because it defines a posterior distribution over functions consistent with the training data. This allows the GPR to provide both a posterior mean prediction and a calibrated predictive uncertainty for each test and validation data point. However, the main limitation of GPR is its poor scalability with data set size, as the training cost of exact GPR grows as $O\left(n^{3}\right)$ with the number of training points. To keep the training tractable, a subset of ca. 5000 points was selected from the full ∼1.9 × 10^4^-point data set by stratified sampling over *T* and *P* using 10 bins for each variable, ensuring adequate coverage of the state-space variables. With a 70/15/15 split, this left ca. 3500 data points for training. The GPR model was trained on the dimensional residual Δγ = γ_cDFT_ – γ_base_ using the feature set X={T,P,z}, which is the same feature set used for the full data-driven ML models. The model reproduced the residual field with a test *R* ^2^ of 0.99, an RMSE of 0.016 mN m^–1^ for both the test and validation data sets, and an MAE of 0.006 mN m^–1^. After reconstructing the IFT using γ̂ = γ_base_ + Δγ̂, the model achieved *R* ^2^ ≈ 1 for both the test and validation sets, with a reconstructed IFT RMSE of 0.02 mN m^–1^ for both data sets, as shown in Figure 9c,d. Because γ_base_ is a fixed quantity, the reconstruction leaves the absolute errors unchanged, so the reconstructed-IFT RMSE and MAE equal their residual-space values, whereas the reconstructed-IFT *R* ^2^ is rescaled to the larger variance of γ_cDFT_ as 1 – RMSE^2^/Var­(γ_cDFT_). A 5-fold CV was performed to further confirm the robustness of the model. The corresponding reconstructed-IFT CV scores are summarized in Table 4. From Table 4, it is evident that the GPR correction outperforms the SR correction in terms of absolute accuracy while additionally providing a per-point uncertainty estimate, although this improvement comes with a strict limitation on the trainable data set size. To overcome this scalability limit and use the full data set, a Stochastic Variational Gaussian Process Regression (SVGP) model was used. The SVGP model uses inducing points and a variational approximation to retain the probabilistic, uncertainty-aware character of GPR, while reducing the training cost so that it scales linearly with the number of data points. Figure 9e and f shows the parity plot and prediction-error analysis for the SVGP hybrid model, respectively. In contrast to the exact GPR, the SVGP model approximates the full posterior using 2000 inducing points. This strategy allowed the model to be trained on the complete ∼1.9 × 10^4^ data set while retaining per-point predictive uncertainty. Using the same residual target, Δγ, the SVGP model reproduced the residual field with a test *R* ^2^ of 0.996, an RMSE of 0.037 mN m^–1^, and an MAE of 0.010 mN m^–1^. For the validation set, the model produced an *R* ^2^ = 0.998 and an RMSE of 0.026 mN m^–1^. After reconstructing the IFT, the model achieved *R* ^2^ ≈ 1 on both the test and validation sets, as shown in Figure 9e. The reconstructed IFT RMSE values shown in Figure 9f were 0.04 and 0.03 mN m^–1^ for the test and validation sets, respectively, while the corresponding MAE values were 0.01 mN m^–1^ for both data sets. 5-fold CV also confirmed the robustness of the SVGP model (Table 4), with a very small spread for all of the folds, indicating robust performance.

Figure 10a,b shows the posterior mean ⟨Δγ⟩ and its predictive uncertainty σ_Δγ_ within the two-phase *PT* envelope considered in this study for the exact GPR model. The markers in Figure 10a represent the training data overlaid, sharing the same color map as the posterior mean. The posterior mean is negative throughout the envelope, ranging from ca. −0.02 to −2.84 mN m^–1^. This is consistent with the actual training residuals spanning a range of 0 to ca. −5 mN m^–1^ (Figure 8b). In Figure 10a, the underestimation of IFT is most pronounced at low *T* and high *P*, where the overlaid training markers show the largest negative residuals, reaching ca. −5 mN m^–1^ at *T* ≈ 200 K and *P* ≈ 160 bar. In this region, the posterior-mean surface reaches only ca. −2.8 mN m^–1^, indicating sparse training data. The exact GPR nevertheless reproduces these markers accurately (test *R* ^2^ = 0.99, RMSE = 0.016 mN m^–1^), so the apparent outlier reflects that more training data at high pressures need to be included for training to improve the posterior mean predictions. From Figure 10b, the predictive uncertainty rises sharply in this same high-pressure region, where σ_Δγ_ exceeds 2.4 mN m^–1^ above 100 bar, also reflecting the sparse training coverage in the high-pressure zone. This also reflects that more CO_2_–H_2_-based mixtures need to be added to generate the training data set for GPR. Figure 10c,d show the ⟨Δγ⟩ and σ_Δγ_ of the SVGP model. Compared to the exact GPR, the overlaid training data are well reproduced by the SVGP, especially in the low-temperature and high-pressure zones, where the posterior-mean surface reaches ca. −4.6 mN m^–1^ and closely matches the dark markers, in contrast to the exact GPR surface that flattened to ca. −2.8 mN m^–1^. As observed in Figure 10c, ⟨Δγ⟩ exceeds 3.8 mN m^–1^ in magnitude for pressures larger than ca. 100 bar, showing that training the SVGP on the full data set yields better predictions of Δγ. This improved performance is also reflected in the σ_Δγ_ contour shown in Figure 10d, which reveals that σ_Δγ_ is well below ca. 1.2 mN m^–1^. This is roughly half the peak uncertainty of the exact GPR (ca. 2.4 mN m^–1^), with the largest values again confined to the high-pressure zones. Although the residual-space accuracy of the SVGP model (Table 4) is marginally lower than that of the exact GPR trained on the 3500-point subset, the SVGP attains better performance while training on the full data set and scaling linearly with the number of data points. This makes the SVGP a more practical uncertainty-aware correction for large data sets.

**Figure 10:**
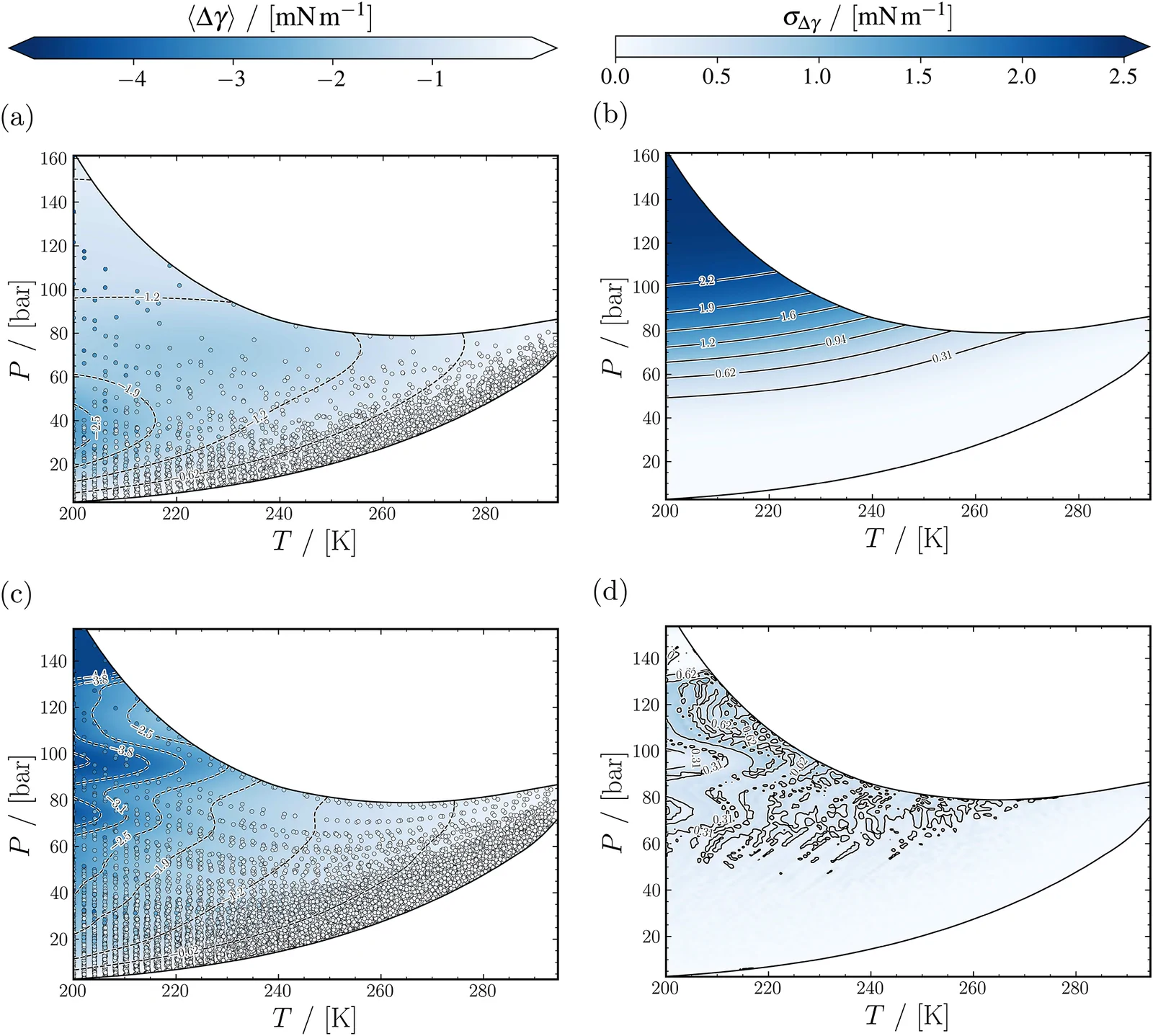
Two-dimensional response surfaces of the hybrid residual Machine Learning (ML) models over the *PT* phase envelope for (a, b) Gaussian Process Regression (GPR) and (c, d) Stochastic Variational Gaussian Process Regression (SVGP). (a, c): Posterior-mean interfacial tension (IFT) residual ⟨Δγ⟩, with the training data overlaid as points colored on the same scale. (b, d) Predictive standard deviation σ_Δγ_. Each column shares the color bar at the top of the column. The black curves denote the bubble and dew boundaries.^87,98–100^

In summary, the SVGP learns the residual function in Figure 8c more accurately than the exact GPR, whereas the corrected WSD expression obtained from SR reproduces the same residual only approximately, with a reconstructed-IFT CV RMSE of ca. 0.1 mN m^–1^ for the full physics-informed feature set (Table 4). The SR correction is nonetheless the most computationally efficient route to compute IFT, since its WSD expression carries essentially no inference cost, illustrating the trade-off between accuracy and computational efficiency. To compare the two best ML surrogate strategies investigated in this study, namely, the full data-driven model (TabPFN with HPO) and the residual-correction model (SVGP), we examine their CV RMSE and MAE in Tables 3 and 4. Both models predict IFT accurately, with CV RMSE values of 0.040 mN m^–1^ (TabPFN) and 0.023 mN m^–1^ (SVGP). Likewise, the CV MAE of SVGP (0.012 mN m^–1^) is about 1.7 times lower than that of TabPFN (0.020 mN m^–1^). We further compared the per-state-point inference time, TabPFN with HPO infers in ca. 1.7 ms on an NVIDIA H100 GPU, whereas the SVGP requires ca. 5.2 ms on an Intel Xeon Silver 4214R CPU. On the corresponding hardware, the SVGP is about three times slower, reflecting the same accuracy versus cost trade-off. Regardless of the surrogate, both TabPFN and SVGP reproduce the IFT of the PCP-SAFT EoS + cDFT reference with high accuracy. To quantify the cost, we benchmarked the wall time to compute the IFT map (similar to Figure 2d) of the multicomponent CO_2_ mixtures listed in Table 2 with temperature-dependent *k*_*ij*_ on the AMD EPYC 9754 node and reduced it to a per-state-point cost. This cost ranges from ca. 184 ms for the binary system to ca. 1791 ms for the 8-component system. The cDFT wall time generally increases with the number of components, roughly by 1 order of magnitude from the 2-component to the 8-component feeds. However, the trend is not strictly monotonic because the cDFT cost also depends on the convergence behavior of the coupled density profiles, which varies between mixtures. Consequently, for each state point, the cDFT reference computation is approximately 2–3 orders of magnitude slower than TabPFN and 1–2 orders of magnitude slower than SVGP. This confirms that the ML surrogates reduce the physics-engine cost by 2–3 orders of magnitude with negligible loss of accuracy.

## 4 Conclusions

In this study, we used the PCP-SAFT EoS + cDFT framework to compute the phase equilibria and interfacial properties of multicomponent CO_2_-rich mixtures. The phase-equilibrium properties include the bubble- and dew-point pressures in the *PT* phase envelope, while the interfacial properties include the IFT and interfacial thickness. The mixtures contain seven CO_2_ transportation-relevant impurities, namely, CH_4_, H_2_, N_2_, Ar, O_2_, CO, and H_2_S. To obtain reliable mixture predictions, the values of *k*_*ij*_ were first optimized for the 23 binary CO_2_-containing pairs using available experimental *P*_*xy*_ data. For binary systems with sufficient data at different temperatures, temperature-dependent *k*_*ij*_ correlations were fitted to capture the variation in phase behavior with temperature. Using the optimized BIP, we generated a data set of ca. 1.9 × 10^4^ points for 100 multicomponent CO_2_-rich mixtures. The mixture compositions were sampled by combining Dirichlet sampling, systematic scaling, and CO_2_ feeds from standards and operating pipelines in the ratio 0.7:0.2:0.1. A sensitivity analysis was performed for five multicomponent CO_2_-rich mixtures with varying compositions and numbers of components from the 100 multicomponent CO_2_-rich mixtures. The results showed that the dew-point pressure and interfacial thickness are less sensitive to impurity concentration than the bubble-point pressure and the IFT at the bubble pressure. The bubble-point pressure and bubble-point IFT both respond strongly to the impurity content. Screening of the full data-driven ML models was performed using the same input variables used to plan an experiment or molecular simulation, namely temperature (*T*), pressure (*P*), and mixture composition (**z**). Compared to the tested models, TabPFN with HyperParameter Optimization (HPO) provided the best balance between accuracy and robustness for all four properties, especially for the impurity-sensitive *P*_bubble_ and γ, for which HPO was most beneficial, reaching 5-fold CV RMSE values of 1.31 bar for *P*_bubble_ and 0.040 mN m^–1^ for γ. The next-best models were XGB, RF, and SVR, except for *P*_dew_, where RF and XGB showed comparable performance. The SHAP analysis further confirmed that the prediction of *P*_bubble_ is influenced primarily by the H_2_ concentration, followed by *T* and *P*, whereas γ is governed mainly by *T* and *P*. It is also shown that TabPFN captures more composition-dependent impurity interactions than XGB. We also evaluated the number of feed compositions required to train the full data-driven ML models to a given accuracy by comparing random, stratified, and GPR-based active-learning sampling strategies. The results showed that active learning is the most data-efficient strategy and that ∼50 feed compositions already provide a favorable accuracy-cost balance for the CO_2_-rich multicomponent mixtures. Active learning is therefore the strategy we recommend for extending the data set to new impurity types. In addition, we developed a physics-informed hybrid model for IFT, in which the semiempirical WSD correlation provides the baseline γ_base_, and an ML model learns its residual obtained from the PCP-SAFT EoS + cDFT. The IFT is then reconstructed as γ̂ = γ_base_(1 + ϵ̂_γ_). Using SR, we obtained the interpretable correction ${\mathrm{\epsilon ̂}}_{\gamma }=\ln\left(\Delta \rho^{*}\right)/\left[\sqrt{P_{r}^{*}}+\left(\gamma_{r}^{*}-\Delta \rho^{*}\right)\right]$ , with a CV RMSE of 0.11 mN m^–1^. The SVGP, trained on the full data set, achieved the lowest CV RMSE of 0.023 mN m^–1^, outperforming the exact GPR, which was limited to a ∼3500-point subset and yielded 0.038 mN m^–1^. Notably, the best residual-correction model (SVGP, 0.023 mN m^–1^) outperformed the best full data-driven model for IFT (TabPFN with HPO, 0.040 mN m^–1^), confirming that embedding the WSD physical baseline improves the prediction of IFT. Finally, benchmarking the computational cost showed that a single cDFT evaluation of γ ranges from ca. 184 to 1791 ms as the system size increases from the binary to the 8-component system. In sharp contrast, the trained surrogates predict a state point in ca. 2 ms for TabPFN and ca. 5 ms for SVGP, resulting in substantial speed-ups with negligible loss of accuracy. This rapid inference is particularly relevant for dynamic CO_2_ transportation simulations, including CFD-based models and transient multiphase flow simulators such as Olga^122^ and LedaFlow,^123,124^ where efficient pipeline-scale computations require fast property evaluations or precomputed lookup tables. In such applications, the surrogate models act as a composition-aware replacement for precomputed lookup tables, taking *T*, *P*, and **z** as direct inputs so that no new table is required per feed. To the best of our knowledge, this is the first systematic comparison of the physics engine, PCP-SAFT + cDFT, with two complementary ML surrogate strategies for the interfacial and phase-equilibrium properties of multicomponent CO_2_-rich mixtures relevant to CO_2_ transportation. These strategies include a full data-driven model and a physics-informed residual-correction model. This framework enables rapid and uncertainty-aware estimation of IFT and saturation properties for multiphase process simulations and inverse design. This study supports the design of experiments and molecular simulations by prescreening the most informative feed compositions for measurement or simulation and by identifying the most suitable ML model for each target property.

## Supplementary Material





## Data Availability

All raw and processed data supporting this work are available in the Supporting Information and are archived in an open Zenodo repository (DOI: 10.5281/zenodo.21825307).

## Notes

Repository contains the raw and processed data, the trained machine-learning models, and their predictions supporting this manuscript (ZIP)
PCP-SAFT EoS pure-component parameters for CO_2_, H_2_, Ar, N_2_, CH_4_, O_2_, CO, and H_2_S; the methodologies used to determine the temperature-dependent binary interaction parameter (BIP) (*k*_*ij*_) by fitting to experimental *P*_*xy*_ data, the experimental *P*_*xy*_ data for the binary CO_2_ pairs, and the resulting *k*_*ij*_(*T*) correlations; the stratified composition-space sampling algorithm with *k*-means clustering and elbow/silhouette cluster selection; the kernel variance active-learning sampling algorithm with ARD feature importance and learning curves; the full data-driven ML parity plots for bubble-point pressure, dew-point pressure, IFT, and interfacial thickness using RF, SVR, XGB, and TabPFN, together with the model hyperparameter optimization details; the SR pure-component IFT correlations (Guggenheim–Katayama form); and the hybrid WSD + SR residual-correction model for the IFT. The supporting material also provides the experimental *P*_*xy*_ data (in Excel format), the data sets used to train the ML models, which contain the raw and processed phase-equilibrium and interfacial-property data of the multicomponent CO_2_-rich mixtures, and the trained TabPFN and SVGP models, together with their test and validation set predictions (PDF)
