# Supplementary material for: Machine Learning-Based Prediction of Phase Equilibria and Interfacial Properties of Multicomponent CO2 Mixtures with Impurities: Application to CO2 Transportation
Source: Ind Eng Chem Res. 2026 Aug 28;65(35):18863–90. doi: 10.1021/acs.iecr.6c03388 (PMC13564437; doi:10.1021/acs.iecr.6c03388)
Supplement: Supplementary file 1 [file ie6c03388_si_002.pdf]

Supporting Information for: Machine Learning-Based Prediction of Phase Equilibria and Interfacial Properties of Multi-component CO_2 Mixtures with Impurities: Application to CO_2 Transportation

Darshan Raju[†], Roar Skartlien[‡], Mahinder Ramdin[†], and Thijs J.H. Vlugt[*,†]

†*Engineering Thermodynamics, Process & Energy Department, Faculty of Mechanical Engineering, Delft University of Technology, Leeghwaterstraat 39, Delft 2628CB, The Netherlands*

‡*Institute for Energy Technology, P.O Box 40, Kjeller, N-2027, Norway*

E-mail: T.J.H.Vlugt@tudelft.nl

The following items are presented in this Supporting Information:

1. The Perturbed Chain Polar Statistical Associating Fluid Theory (PCP-SAFT) equation of state (EoS) parameters of pure components used to compute phase envelopes and interfacial properties of multi-component CO_2 mixtures in Framework for Equations of State and Classical Density Functional Theory (FeO_s) (Table S1).

2. Temperature-dependent binary interaction parameters (k_{ij}s) of dissimilar molecule pairs used in the PCP-SAFT EoS, including the $k_{ij}(T)$ polynomial fit expressions and experimental Vapor-Liquid Equilibrium (VLE) sources (Tables S2–S3) and the corresponding temperature-dependence of $k_{ij}(T)$ (Figures S1–S7).

3. The stratified composition-space sampling algorithm and the clustering diagnostics underlying its strata (Figures S8–S11).

4. Data-driven Machine Learning (ML) parity plots for all four target properties (P_{bubble}, P_{dew}, γ, interfacial thickness) obtained with the Random forest (RF), Support Vector Regression (SVR), eXtreme Gradient Boosting (XGB), and Tabular Prior-data Fitted Network (TabPFN) models (Figures S12–S15), together with hyperparameter selection details comprising the candidate and selected hyperparameter (Table S4), the TabPFN Bayesian optimization search space and selected values (Table S5), and the Root Mean Square Error (RMSE) sweep curves for RF, SVR, and XGB (Figures S16–S25).

5. Symbolic Regression (SR) of the interfacial tension (IFT): pure-component IFT correlations (Tables S6–S7 and Figures S27–S31) and a hybrid classical density functional theory (cDFT)–Winterfeld Scriven Davis (WSD) residual-correction model for CO_2-rich mixtures (Tables S8–S12 and Figures S32–S33).

S1 The Perturbed Chain Polar Statistical Associating Fluid Theory (PCP-SAFT) equation of state (EoS) parameters of pure components to compute the phase envelopes and interfacial properties of multi-component CO_2 mixtures in FeO_s

Component	M [g mol^{-1}]	m [-]	ε/k_B [K]	σ [Å]	q [D.Å]	μ [D]	Association scheme	ε^{AB}/k_B [K]	κ^{AB} [-]	Reference
CO_2	44.010	1.6298	163.34	3.0867	3.9546	-	-	-	-	Gross 2005 [1]
H_2	2.016	0.6800	31.57	3.5400	-	-	-	-	-	Alanazi et al. 2022 [2]
Ar	39.962	1.0000	117.81	3.3775	-	-	-	-	-	Esper et al. 2023 [3]
N_2	28.010	1.1879	90.99	3.3353	1.1151	-	-	-	-	Gross 2005 [1]
CH_4	16.031	1.0000	150.07	3.7005	-	-	-	-	-	Esper et al. 2023 [3]
O_2	31.990	1.1470	113.63	3.1793	-	-	-	-	-	Esper et al. 2023 [3]
CO	27.995	1.3229	91.17	3.2453	-	-	-	-	-	Esper et al. 2023 [3]
H_2S	33.988	1.3330	235.54	3.3320	2.9380	0.978	$2A$–$2B$	566.3	0.001	Tang and Gross 2010 [4]

Table S1: PCP-SAFT pure-component parameters for CO_2, H_2, Ar, N_2, CH_4, O_2, CO, and H_2S. Here, M is the molar mass, m is the segment number, ε and σ are the segment energy and size parameters, q is the quadrupole moment, μ is the dipole moment, and ε^{AB} and κ^{AB} are the association energy and association volume, respectively.

S2 Temperature-dependent binary interaction parameters (k_{ij}s) of dissimilar molecule pairs used in the PCP-SAFT EoS for phase envelopes and interfacial properties computation in FeO_s

The binary interaction parameters k_{ij}s were estimated using the PCP-SAFT EoS[5,6] from FeOs v0.9.5 framework[7] by performing three separate nonlinear least-squares optimizations that minimize the deviation between experimental and computed VLE data. For each trial value of k_{ij}, the phase equilibrium properties at a constant temperature, including compositions, saturation pressure, and chemical potentials, are computed using the PCP-SAFT EoS. Depending on the formulation of the objective function, the residuals were defined either as the distance between computed and experimental points in the Pxy phase diagram, as the difference in chemical potentials of the coexisting phases, or as the difference between computed and experimental saturation pressures. In all cases, the optimization cost (objective) function was expressed as

$$\text{Cost}(k_{ij}) = \sum_{n=1}^{N} [r_n(k_{ij})^2], \tag{S1}$$

where $r_n(k_{ij})$ denotes the appropriate residual for the nth data point and N is the number of experimental VLE data points at the considered temperature. The minimization was carried out using the `scipy.optimize.least_squares` algorithm, which iteratively adjusts k_{ij} to reduce $\text{Cost}(k_{ij})$. After each optimization, the quality of the resulting k_{ij} was assessed by computing the Mean Absolute Percentage Deviation (MAPD) for the residuals of distance, saturation pressure, and chemical potential. The Pxy diagrams of all the dissimilar binary pairs are evaluated at multiple temperatures using the optimized k_{ij} values. The k_{ij} values that produced the lowest MAPD relative to the experimental data were selected to compute the phase equilibria and interfacial properties with PCP-SAFT EoS + cDFT for multi-

component CO_2-rich mixtures. The optimized single-temperature k_{ij} values were then fitted as polynomial functions of temperature,

$$k_{ij}(T) = \sum_{m=0}^{M} a_m T^m, \tag{S2}$$

where $M \in \{0, 1, 2\}$ corresponds to a constant ($M = 0$), linear ($M = 1$), or quadratic ($M = 2$) fit. For an N-component mixture, $N(N-1)/2$ binary interaction parameters are required. The eight-component system considered in this study (CO_2, H_2, Ar, N_2, CH_4, O_2, CO, and H_2S) therefore requires 28 binary pairs. Experimental Pxy data were available for 23 of these pairs, for which the temperature-dependent k_{ij} values were obtained as described above. For the remaining five pairs, CH_4–O_2, H_2–O_2, Ar–H_2S, O_2–CO, and O_2–H_2S, no experimental Pxy data were available in the literature, to the best of our knowledge. Hence, k_{ij} was set to zero for these pairs. The functional form of the k_{ij} correlation, namely linear, constant, or quadratic, was selected based on the temperature range of interest, 200–300 K, and the temperature range for which each binary pair exhibits a two-phase region. For example, the H_2–O_2 pair forms a two-phase region only between 100 and 141 K, which lies outside the temperature range considered in this work. Instead of extrapolating a temperature-dependent correlation beyond its two-phase range, a constant k_{ij} value was adopted and assumed to remain representative for 200–300 K. This choice provides the closest reliable estimate available for such binary pairs. Only binary interaction parameters were used in this work. Multi-component phase behavior was predicted using the corresponding binary interaction parameters, without introducing additional ternary or higher-order correction terms. The resulting numerical expressions for 23 binary pairs are listed in Tables S2 and S3. The corresponding experimental data used for obtaining the $k_{ij}(T)$ expression are provided as Excel (`.xlsx`) files located in the folder `BINARY_INTERACTION_PARAMETERS/KIJ/⟨pair⟩/`.

Table S2: Binary interaction parameter expressions as a function of temperature $k_{ij}(T)$ and the experimental VLE (Pxy data) sources used in the fitting of binary pairs consisting of CO_2, H_2, and Ar. For each pair, the fit type (constant, linear, or quadratic) was selected as the polynomial order, while pairs without experimental data were assigned $k_{ij} = 0$ (fit type zero). Experimental data are provided as Excel (`.xlsx`) files located in the folder `BINARY_INTERACTION_PARAMETERS/KIJ/⟨pair⟩/`.

Binary pair	Fit	T range (K)	$k_{ij}(T)$ expression	Experimental data source(s)	Data file
CO_2–H_2	linear	220–290	$k_{ij}(T) = -9.101\times10^{-4}\,T + 0.03044$	Tsang and Street,[8] Fandiño et al.[9]	`VLE_CO2_H2.xlsx`
CO_2–Ar	quadratic	213–283	$k_{ij}(T) = 2.413\times10^{-5}\,T^2 - 1.183\times10^{-2}\,T + 1.416$	Coquelet et al.,[10] Lasala et al.,[11] Løvseth et al.[12]	`VLE_CO2_AR.xlsx`
CO_2–N_2	quadratic	218–293	$k_{ij}(T) = 7.095\times10^{-6}\,T^2 - 4.015\times10^{-3}\,T + 0.4909$	Lasala et al.,[11] Al-Sahhaf et al.,[13] Brown et al.,[14] Somait and Kidnay;[15] Zenner (1963)	`VLE_CO2_N2.xlsx`
CO_2–CH_4	linear	219–298	$k_{ij}(T) = -7.183\times10^{-4}\,T + 0.1626$	Al-Sahhaf et al.,[13] Wei et al.,[16] Petropoulou et al.,[17] Xu et al.[18]	`VLE_CO2_CH4.xlsx`
CO_2–O_2	quadratic	223–283	$k_{ij}(T) = -4.567\times10^{-6}\,T^2 + 2.139\times10^{-3}\,T - 0.2581$	Fredenslund and Sather,[19] Lasala et al.[11]	`VLE_CO2_O2.xlsx`
CO_2–CO	linear	223–283	$k_{ij}(T) = 8.852\times10^{-4}\,T - 0.3135$	Christiansen et al.,[20] Souza et al.[21]	`VLE_CO2_CO.xlsx`
CO_2–H_2S	linear	258–313	$k_{ij}(T) = 9.174\times10^{-5}\,T + 0.01035$	Chapoy et al.[22]	`VLE_CO2_H2S.xlsx`
H_2–Ar	constant	100–141	$k_{ij} = -4.321\times10^{-2}$	Calado and Streett,[23] Xiao et al.[24]	`VLE_Ar_H2.xlsx`
H_2–N_2	constant	91–110	$k_{ij} = -9.450\times10^{-3}$	Streett and Calado,[25] Xiao et al.[24]	`VLE_N2_H2.xlsx`
H_2–CH_4	constant	92–180	$k_{ij} = -2.054\times10^{-2}$	Tsang et al.[26]	`VLE_CH4_H2.xlsx`
H_2–O_2	zero	—	$k_{ij} = 0$	No experimental data available	—
H_2–CO	constant	85–125	$k_{ij} = -5.680\times10^{-3}$	Tsang et al. (1981)	`VLE_CO_H2.xlsx`
H_2–H_2S	quadratic	243–273	$k_{ij}(T) = 8.068\times10^{-4}\,T^2 - 0.4184\,T + 53.93$	Yorizane et al. (1969)	`VLE_H2_H2S.xlsx`
Ar–N_2	constant	91–121	$k_{ij} = -7.491\times10^{-3}$	Baba-Ahmed et al.,[27] Narinskii[28]	`VLE_Ar_N2.xlsx`
Ar–CH_4	constant	91–178	$k_{ij} = 1.942\times10^{-2}$	Calado and Staveley,[29] Christiansen et al.,[30] Shatskaya and Zhirnova,[31] Sprow and Prausnitz[32]	`VLE_Ar_CH4.xlsx`
Ar–O_2	constant	90–120	$k_{ij} = 9.549\times10^{-3}$	Narinskii,[33] Clark et al.[34]	`VLE_Ar_O2.xlsx`
Ar–CO	constant	123–137	$k_{ij} = -6.832\times10^{-3}$	Christiansen et al.[30]	`VLE_CO_Ar.xlsx`
Ar–H_2S	zero	—	$k_{ij} = 0$	No experimental data available	—

Table S3: Binary interaction parameter expressions as a function of temperature, $k_{ij}(T)$, and the experimental VLE (Pxy data) sources used for fitting binary pairs comprising N_2, CH_4, O_2, and CO. For each binary pair, the fit type, namely constant, linear, or quadratic, was selected based on the available experimental temperature range and the two-phase region relevant to the present study. Pairs without suitable experimental data were assigned $k_{ij} = 0$ and denoted by fit type zero. The notation and fitting conventions are the same as those in Table S2, and the corresponding experimental data are provided as Excel (`.xlsx`) files located in the folder `BINARY_INTERACTION_PARAMETERS/KIJ/⟨pair⟩/`.

Binary pair	Fit	T range (K)	$k_{ij}(T)$ expression	Experimental data source(s)	Data file
N_2–CH_4	constant	140–180	$k_{ij} = 2.904\times10^{-2}$	Kidnay et al.,[35] Somait and Kidnay[15]	`VLE_N2_CH4.xlsx`
N_2–O_2	constant	100–121	$k_{ij} = -8.508\times10^{-3}$	Baba-Ahmed et al.[27]	`VLE_N2_O2.xlsx`
N_2–CO	constant	70–120	$k_{ij} = -2.561\times10^{-4}$	Torocheshnikov[36]	`VLE_CO_N2.xlsx`
N_2–H_2S	linear	200–300	$k_{ij}(T) = 2.150\times10^{-3}\,T - 0.4800$	Besserer and Robinson[37]	`VLE_N2-H2S.xlsx`
CH_4–O_2	zero	—	$k_{ij} = 0$	No experimental data available	—
CH_4–CO	constant	123–178	$k_{ij} = 1.264\times10^{-2}$	Christiansen et al.[30]	`VLE_CO_CH4.xlsx`
CH_4–H_2S	linear	203–273	$k_{ij}(T) = -5.485\times10^{-4}\,T + 0.1807$	Yarym-Agaev et al.,[38] Coquelet et al.[39]	`VLE_CH4-H2S.xlsx`
O_2–CO	zero	—	$k_{ij} = 0$	No experimental data available	—
O_2–H_2S	zero	—	$k_{ij} = 0$	No experimental data available	—
CO–H_2S	quadratic	233–293	$k_{ij}(T) = -3.696\times10^{-5}\,T^2 +2.037\times10^{-2}\,T - 2.755$	Fredenslund and Mollerup[40]	`VLE_CO_H2S.xlsx`

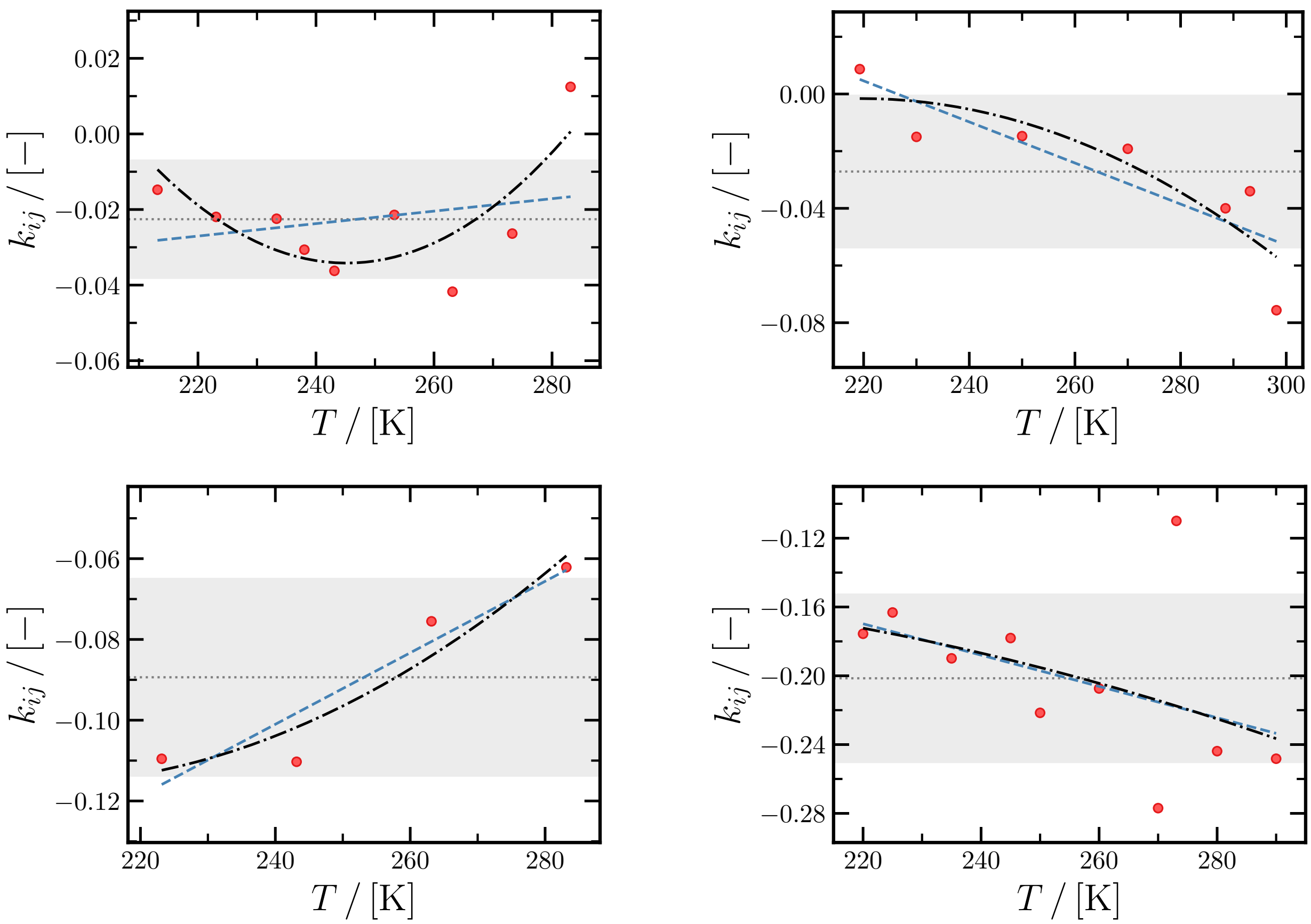

Figure S1: Variation of the binary interaction parameter k_{ij} as a function of temperature for binary mixtures of CO_2 with (a) Ar, (b) CH_4, (c) CO, and (d) H_2. The red markers in each panel indicate the k_{ij} values determined by fitting to experimental data at every temperature for which experimental Pxy data existed, the dotted gray line and surrounding gray band represent the average k_{ij} together with its standard deviation, and the dashed and dash-dotted curves correspond to the linear and quadratic temperature fits, respectively. These polynomial fits were used to estimate k_{ij} at temperatures where experimental VLE data were unavailable.

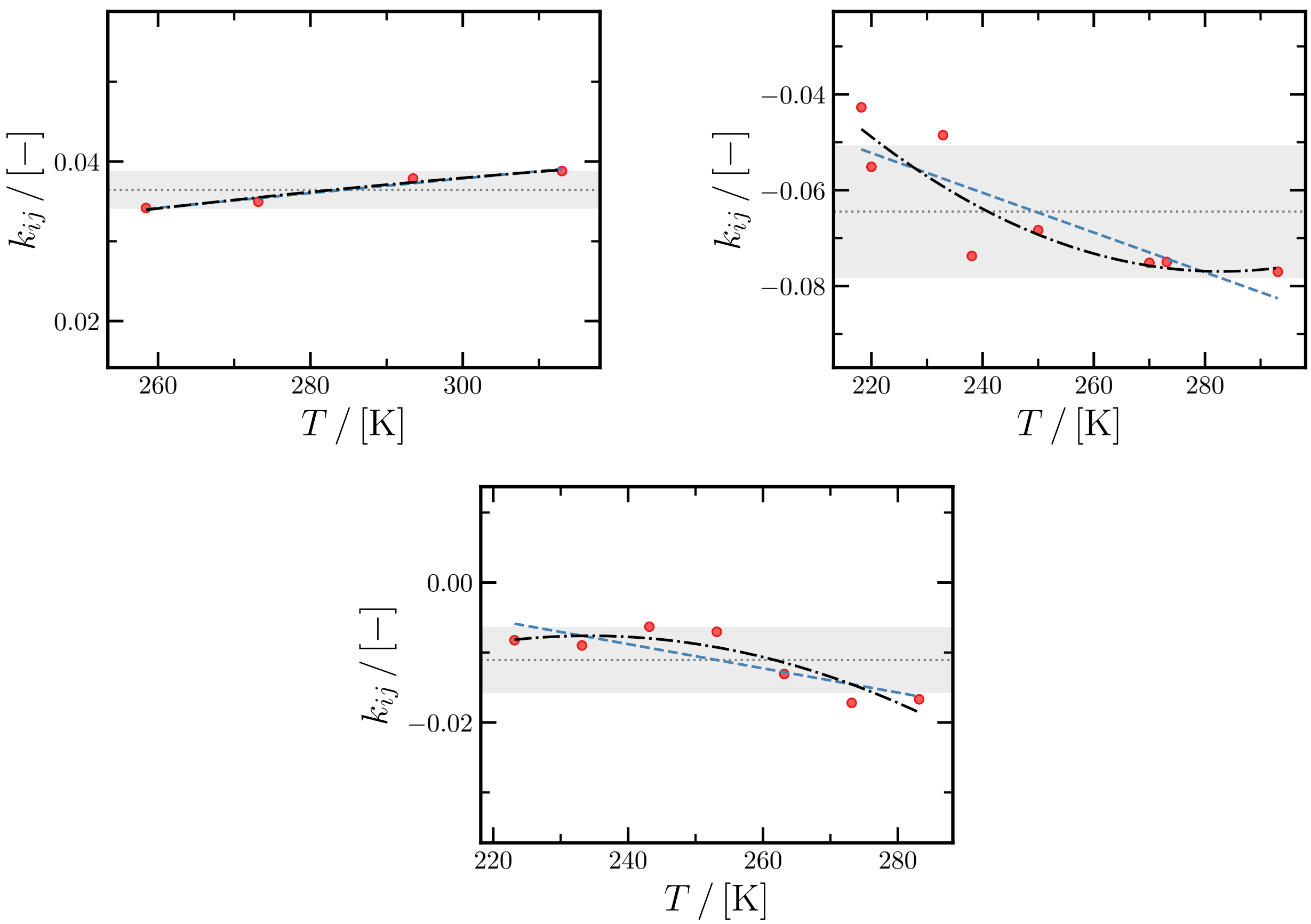

Figure S2: (Continued from Fig. S1.) Temperature dependence of k_{ij} for binary pairs of CO_2 with (a) H_2S, (b) N_2, and (c) O_2. Symbols and lines are as in Fig. S1.

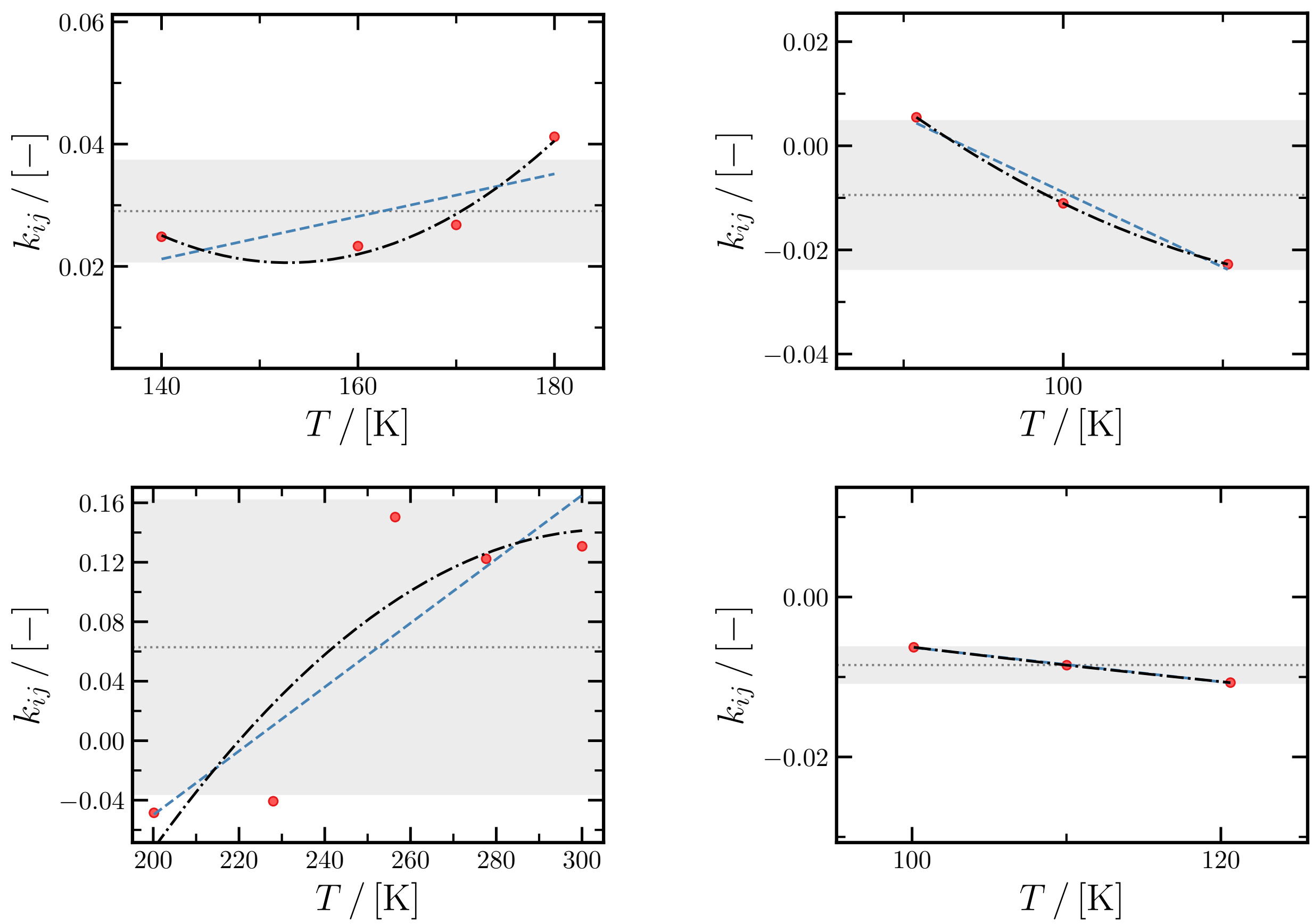

Figure S3: Variation of the binary interaction parameter k_{ij} as a function of temperature for binary mixtures of N_2 with (a) CH_4, (b) H_2, (c) H_2S, and (d) O_2. The red markers in each panel indicate the k_{ij} values determined by fitting to experimental data at every temperature for which experimental Pxy data existed, the dotted gray line and surrounding gray band represent the average k_{ij} together with its standard deviation, and the dashed and dash-dotted curves correspond to the linear and quadratic temperature fits, respectively. These polynomial fits were used to estimate k_{ij} at temperatures where experimental VLE data were unavailable.

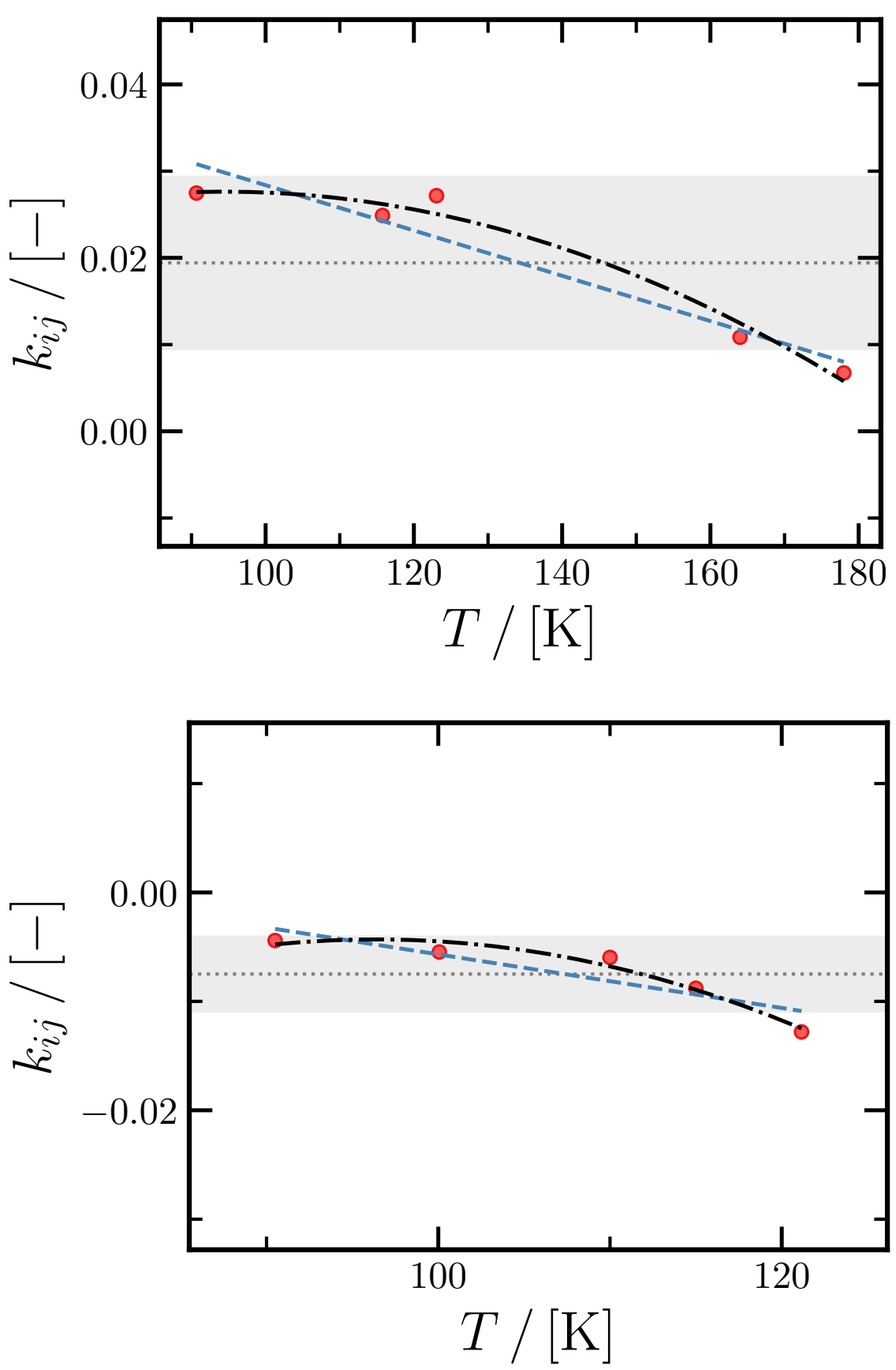

Figure S4: Variation of the binary interaction parameter k_{ij} as a function of temperature for binary mixtures of Ar with (a) CH_4, (b) H_2, (c) N_2, and (d) O_2. The red markers in each panel indicate the k_{ij} values determined by fitting to experimental data at every temperature for which experimental Pxy data existed, the dotted gray line and surrounding gray band represent the average k_{ij} together with its standard deviation, and the dashed and dash-dotted curves correspond to the linear and quadratic temperature fits, respectively. These polynomial fits were used to estimate k_{ij} at temperatures where experimental VLE data were unavailable.

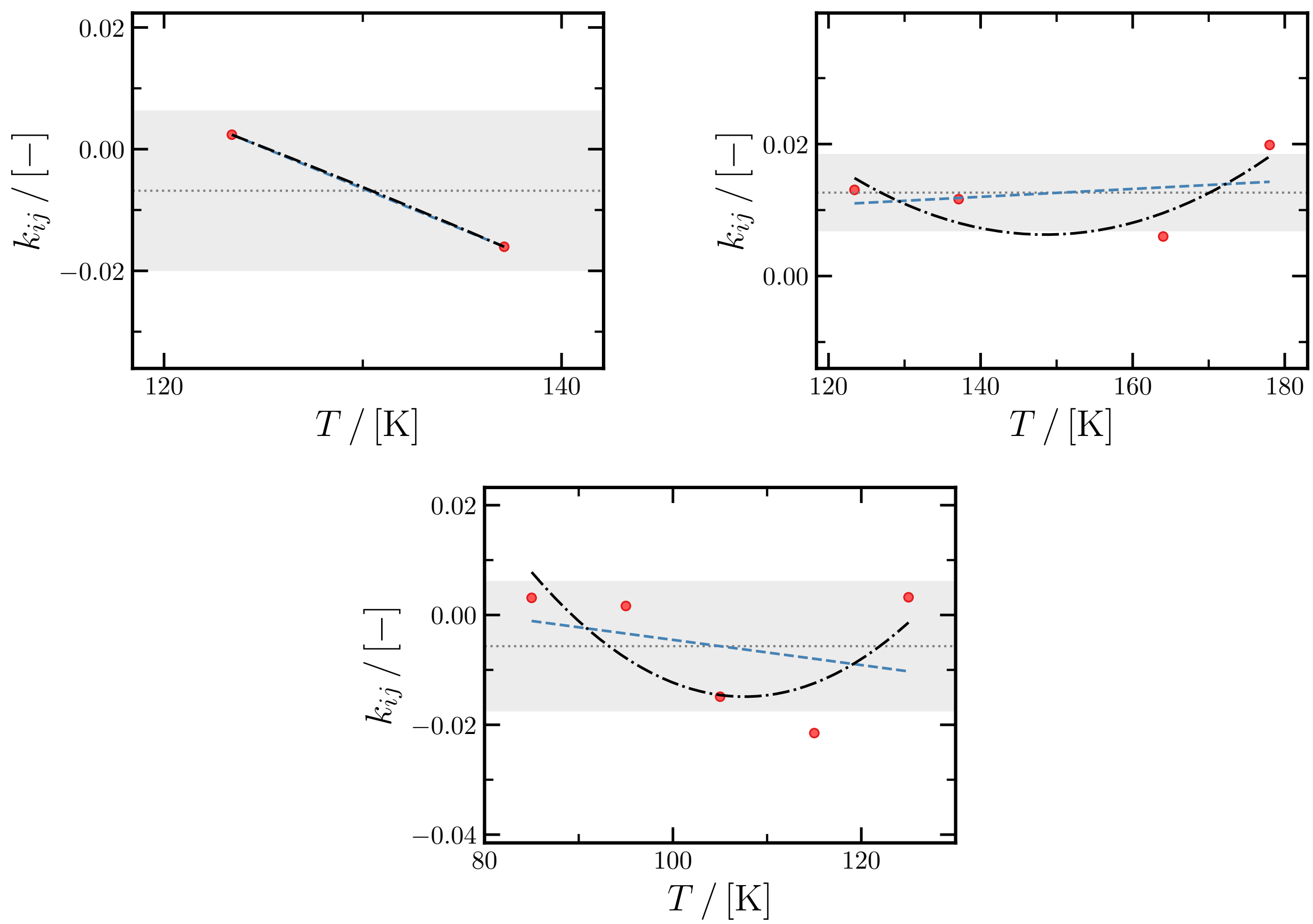

Figure S5: Variation of the binary interaction parameter k_{ij} as a function of temperature for binary mixtures of CO with (a) Ar, (b) CH_4, and (c) H_2. The red markers in each panel indicate the k_{ij} values determined by fitting to experimental data at every temperature for which experimental Pxy data existed, the dotted gray line and surrounding gray band represent the average k_{ij} together with its standard deviation, and the dashed and dash-dotted curves correspond to the linear and quadratic temperature fits, respectively. These polynomial fits were employed to estimate k_{ij} at temperatures lacking experimental VLE data.

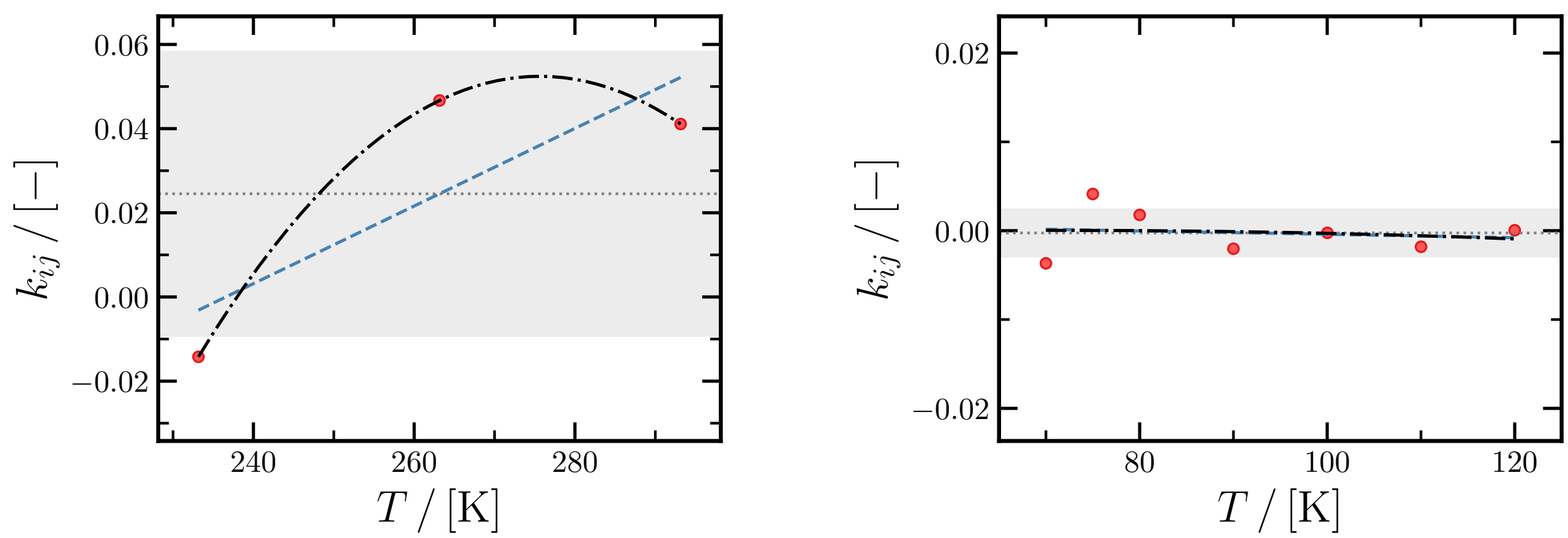

Figure S6: Variation of the binary interaction parameter k_{ij} as a function of temperature for binary mixtures of CO with (a) H_2S and (b) N_2. The red markers in each panel indicate the k_{ij} values determined by fitting to experimental data at every temperature for which experimental Pxy data existed, the dotted gray line and surrounding gray band represent the average k_{ij} together with its standard deviation, and the dashed and dash-dotted curves correspond to the linear and quadratic temperature fits, respectively. These polynomial fits were used to estimate k_{ij} at temperatures where experimental VLE data were unavailable.

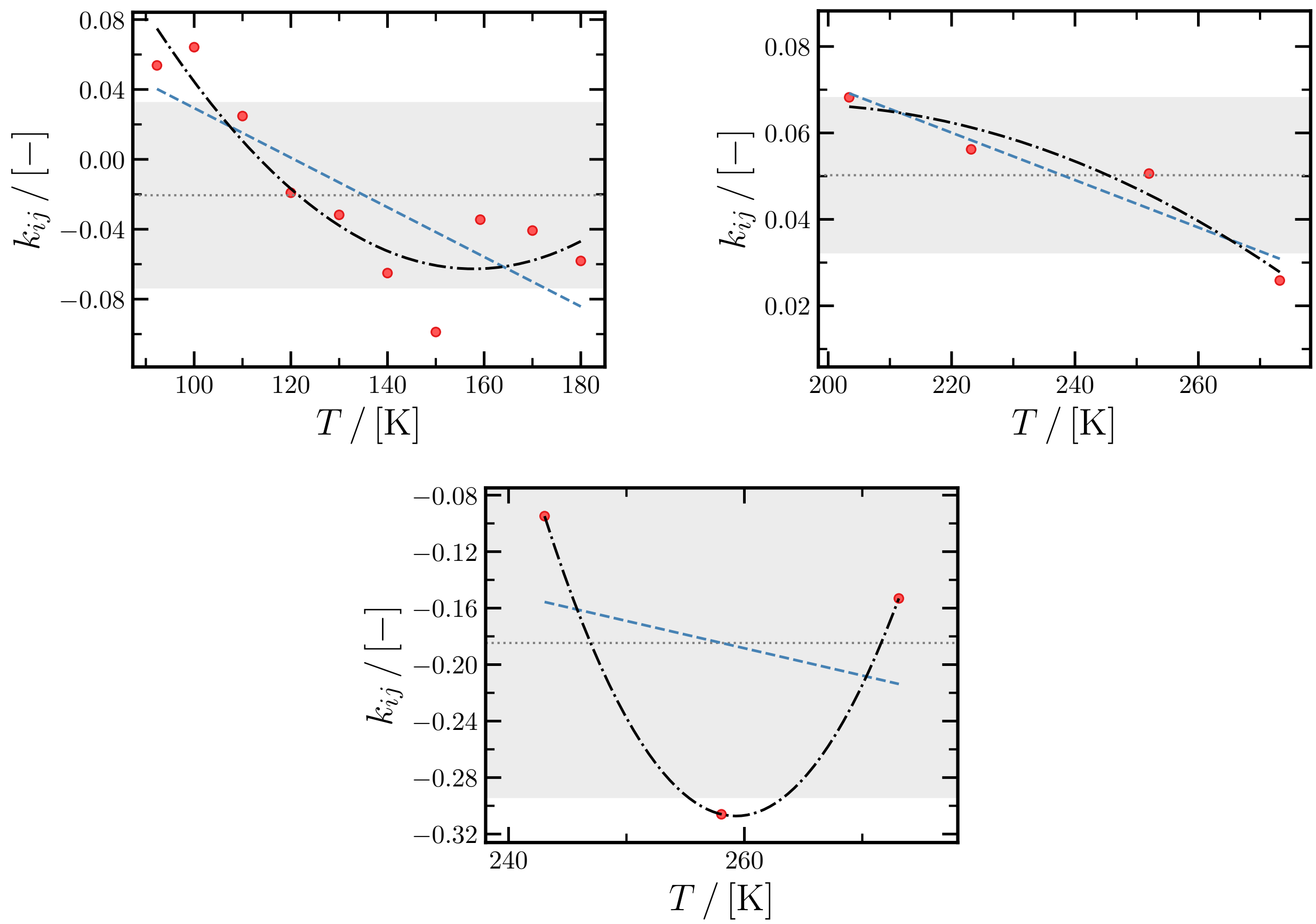

Figure S7: Variation of the binary interaction parameter k_{ij} as a function of temperature for the binary pairs (a) CH_4–H_2, (b) CH_4–H_2S, and (c) H_2–H_2S. The red markers in each panel indicate the k_{ij} values determined by fitting to experimental data at every temperature for which experimental Pxy data existed, the dotted gray line and surrounding gray band represent the average k_{ij} together with its standard deviation, and the dashed and dash-dotted curves correspond to the linear and quadratic temperature fits, respectively. These polynomial fits were used to estimate k_{ij} at temperatures where experimental VLE data were unavailable.

S3 Stratified composition-space sampling

To efficiently sample the composition space of CO_2-rich mixtures with impurities while retaining representative coverage, a stratified sampling scheme was used as an alternative to simple random sampling. The stratification was constructed in two stages. First, the CO_2 mole fraction z_{CO2} was divided into five bins spanning $z_{CO2} \in [0.95, 1.00)$, namely $[0.95, 0.96)$, $[0.96, 0.97)$, $[0.97, 0.98)$, $[0.98, 0.99)$, and $[0.99, 1.00)$. Second, for each CO_2 bin, the impurity composition vectors were normalized by the total impurity fraction, min–max scaled, and grouped into K clusters using k-means clustering.[41] The number of impurity clusters was guided by the elbow method and the silhouette score,[41–43] evaluated separately for each CO_2 bin, as shown in Figs. S8 and S9. As the number of clusters k increases, the inertia (a measure of how tightly points group within each cluster) drops quickly at first and then levels off, with little further gain beyond about $k \approx 6$. The silhouette score, which measures how cleanly the clusters separate, stays roughly flat for this same range. While the silhouette score is marginally higher at $k \approx 7$–8, selecting such a value is not appropriate here, since that would be close to the total number of distinct impurity species in the dataset, meaning we would essentially be giving each impurity its own cluster rather than finding meaningful groupings. This indicates that mixtures dominated by the same impurity would tend to form separate clusters. We therefore chose a fixed $K = 5$ for every bin, which captures most of the grouping structure while keeping the number of strata manageable. With five CO_2 bins and five impurity clusters, we obtain up to 25 strata. Consequently, the minimum sample size we adopt for the composition-space sampling is 25, corresponding to one representative composition per stratum. For a desired total sample size N, we draw samples from each stratum in proportion to the number of compositions it contains in the pool, subject to at least one sample per stratum and exactly N samples overall. To visualize the resulting clustering, the impurity composition vectors of the whole pool were min–max scaled (pooled across all bins to provide a single unified view) and projected onto their first two principal components via Principal Component Analysis (PCA),[44] with the per-bin k-means cluster

assignments distinguished by color in Fig. S10. Since the impurity vectors are normalized to a constant total, the vectors lie on a simplex, whose projection produces the bounded, roughly triangular envelope seen in the figure, with vertices corresponding to compositions dominated by a single impurity species. The first two components capture only 38.1% of the total variance (19.3% and 18.8%, respectively), so this plane reflects only part of the full structure, and the overlap visible here need not imply overlap in the complete feature space. The clusters nonetheless occupy broadly distinct regions, indicating that the stratification reflects genuine structure in the impurity-composition patterns rather than arbitrary divisions. The corresponding stratum populations across the five CO_2 bins and five impurity clusters are shown as a heatmap in Fig. S11.

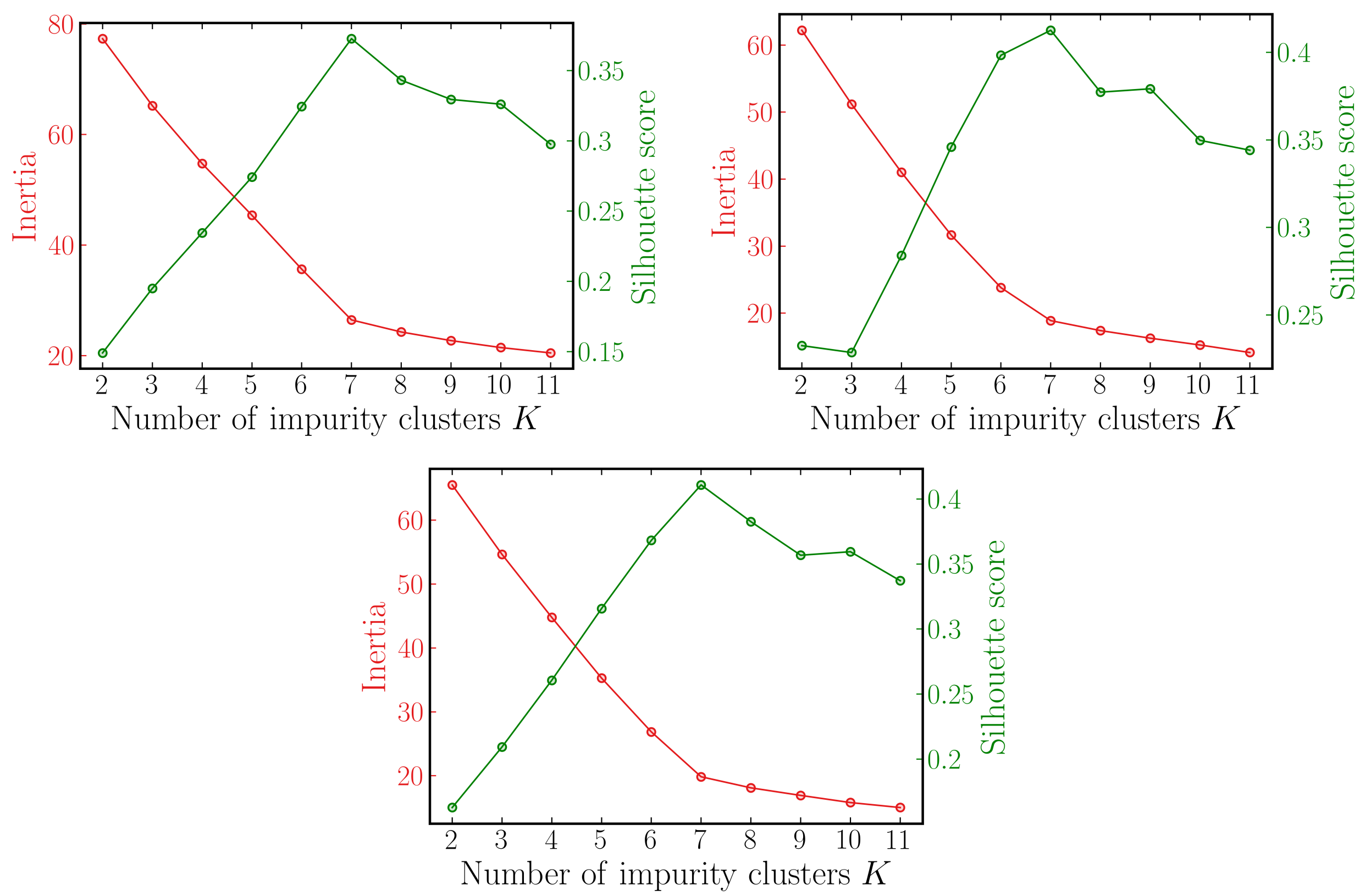

Figure S8: Selection of the number of impurity clusters, K, for stratified sampling using k-means clustering of the per-bin normalized impurity composition vectors. Panels (a)-(c) show the results for the CO_2 mole-fraction bins $z_{CO2} \in [0.95, 0.96)$, $[0.96, 0.97)$, and $[0.97, 0.98)$, respectively. In each panel, the within-cluster sum of squares, or inertia, is plotted on the left axis, while the mean silhouette score is plotted on the right axis as a function of K. Based on this analysis, a fixed value of $K = 5$ was used for all composition bins.

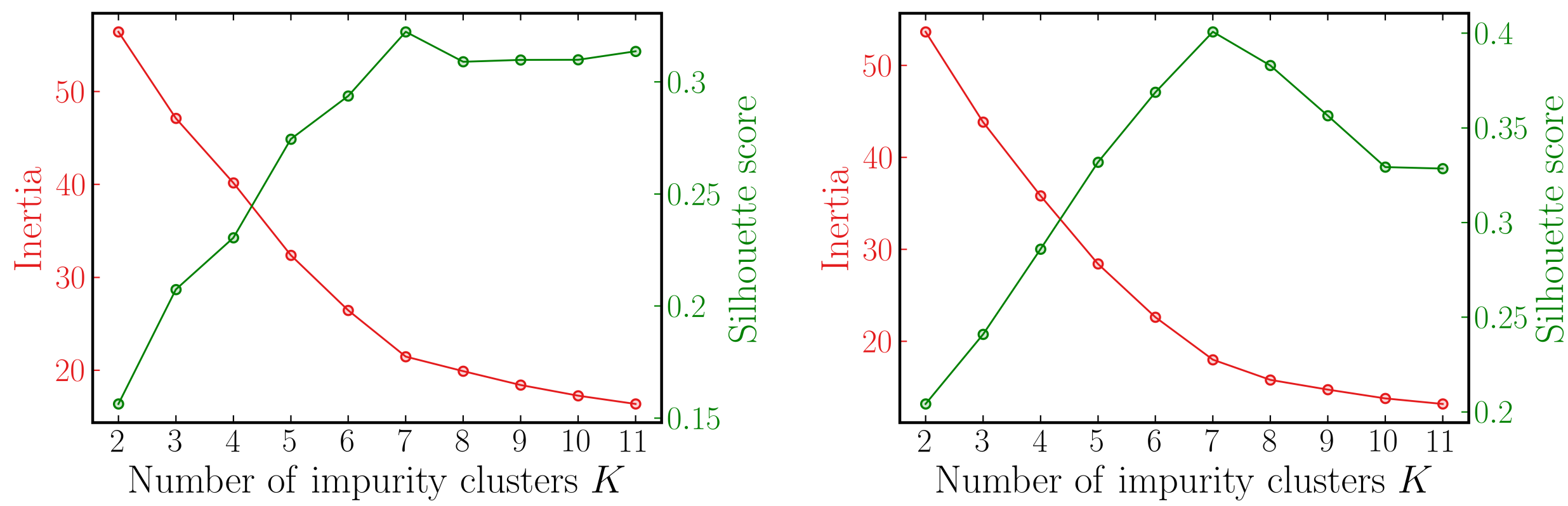

Figure S9: Selection of the number of impurity clusters, K, for stratified sampling using k-means clustering of the per-bin normalized impurity composition vectors. Panels (a) and (b) show the results for the CO_2 mole-fraction bins $z_{CO2} \in [0.98, 0.99)$ and $[0.99, 1.00)$, respectively. In each panel, the within-cluster sum of squares, or inertia, is plotted on the left axis, while the mean silhouette score is plotted on the right axis as a function of K. Based on this analysis, a fixed value of $K = 5$ was used for all composition bins.

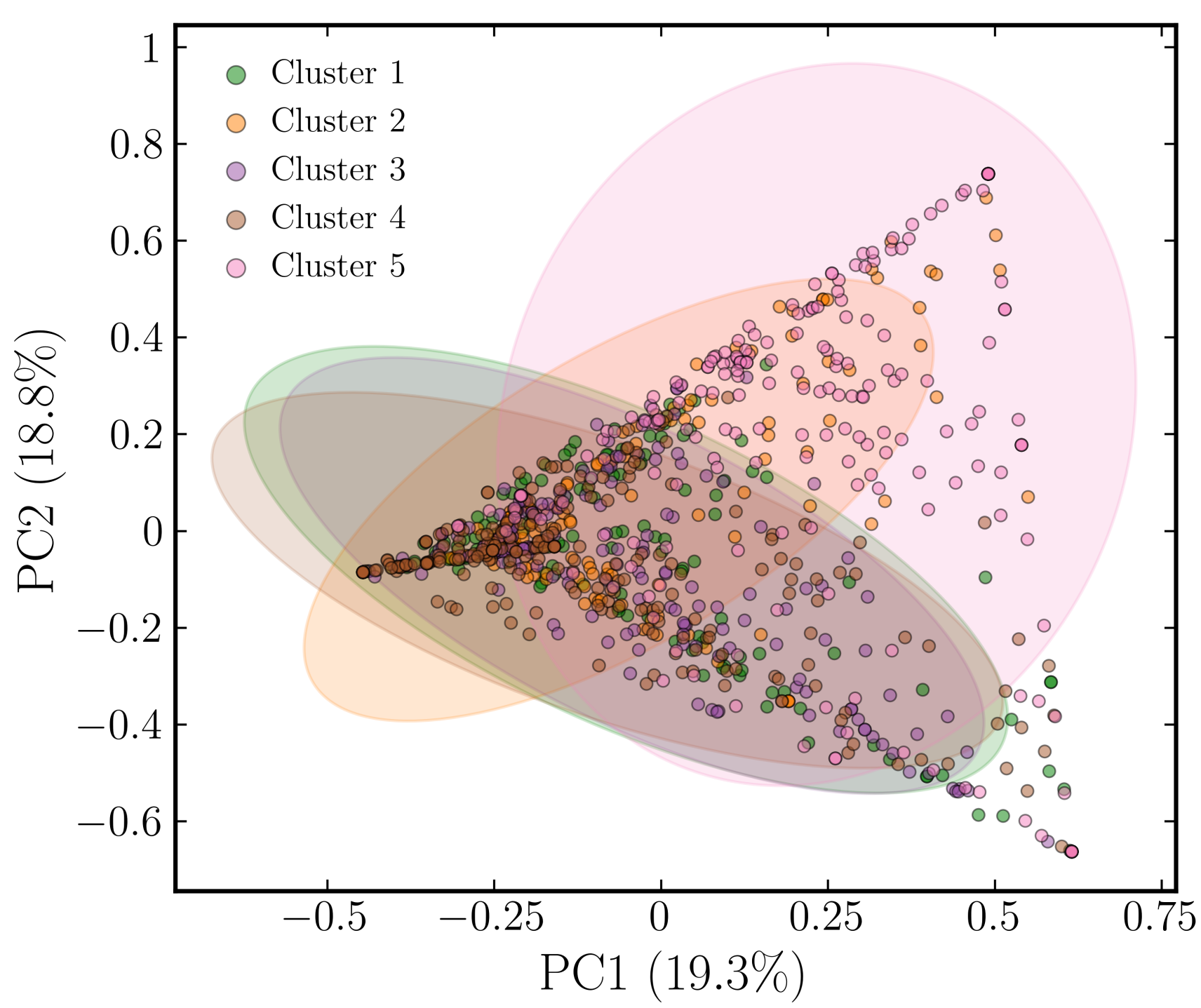

Figure S10: Per-bin k-means impurity clusters projected onto the plane defined by the first two principal components of the min–max scaled impurity composition vectors. Color denotes cluster assignment, shaded ellipses indicate the approximate extent of each cluster, and the axis labels report the variance explained by each principal component.

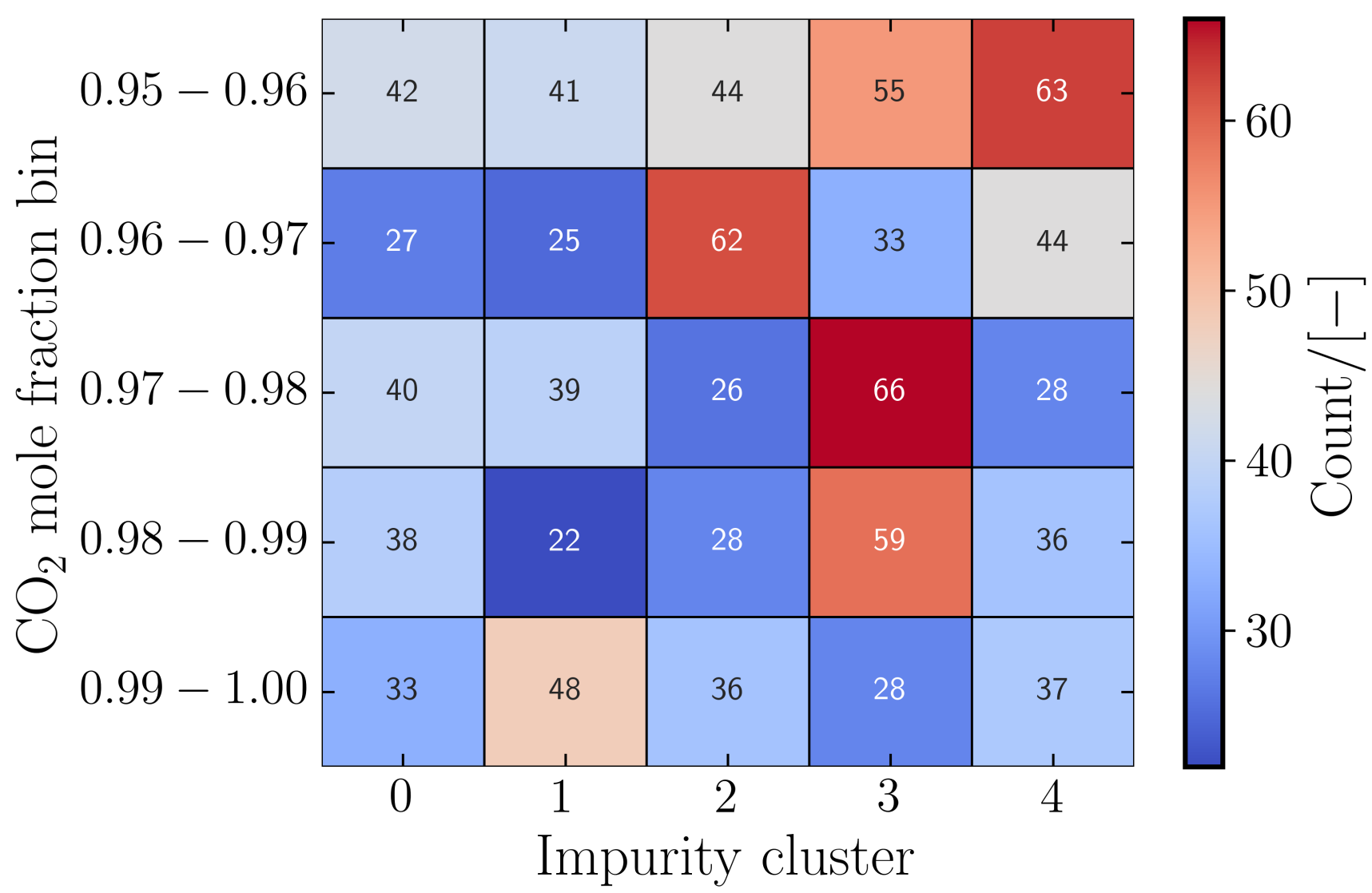

Figure S11: Stratum populations of the composition pool $\mathcal{P}$ of size 1000, reported as the number of compositions in each combined stratum defined by the CO_2 mole-fraction bin and impurity cluster. The non-uniform distribution of compositions across strata motivates the use of proportional allocation when selecting samples.

S4 Full data-driven Machine Learning (ML) models

We present the parity plots Figures S12 to S15 of the four target properties (P_{bubble}, P_{dew}, γ, and interfacial thickness) obtained from the full data-driven ML models (RF,[45] SVR,[46,47] XGB,[48,49] and TabPFN[50]) in Section S4.1. These complement the prediction-error analysis plots reported in the main text (Figure 4) by reporting the coefficient of determination (R^2) for the training, test, and validation splits of each model. In addition, we detail the hyperparameter selection for the RF,[45] SVR,[46,47] XGB,[48,49] and TabPFN[50,51] in Section S4.2

S4.1 Parity plots of full data-driven Machine Learning (ML) models

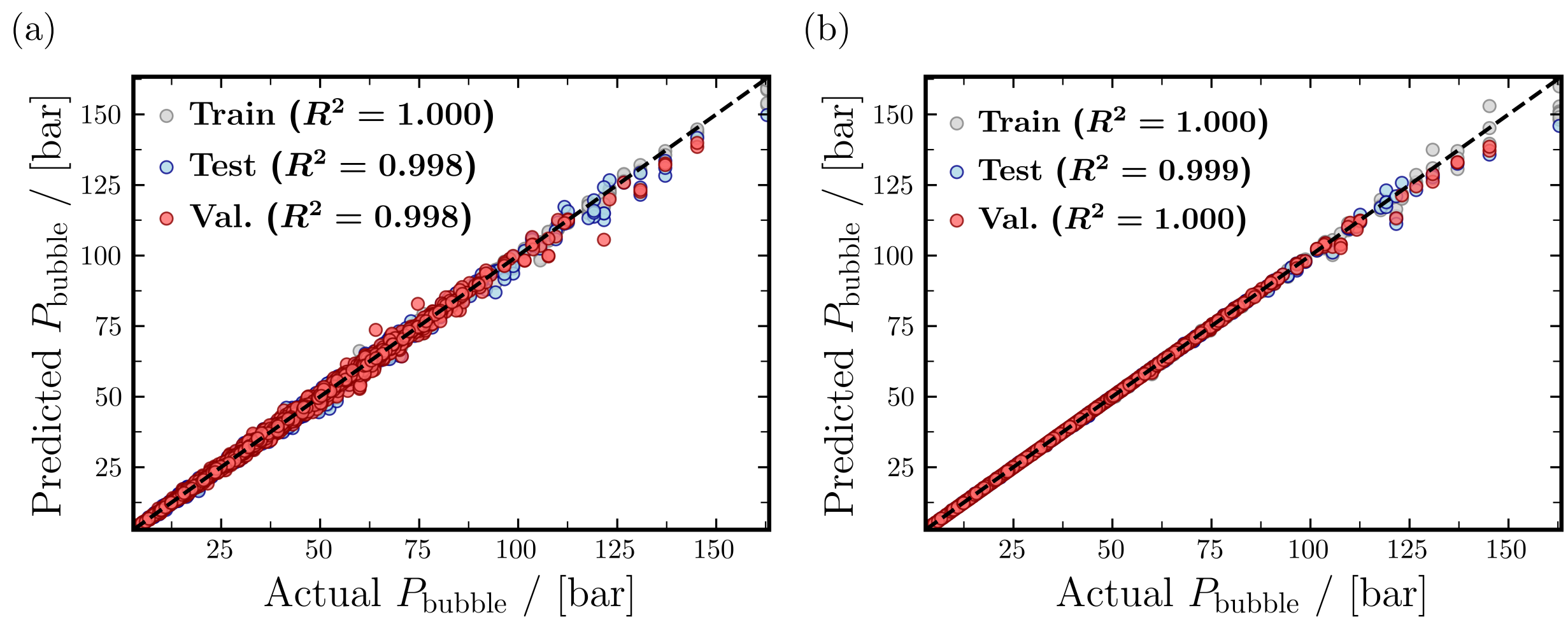

Figure S12: Parity plots for P_{bubble} obtained from (a) RF, (b) SVR, (c) XGB, and (d) TabPFN. The training, test, and validation sets are presented separately to evaluate both fitting and predictive performance on untrained data. The R^2 values reported in each subplot quantify the agreement between the predicted and reference values. The diagonal line represents the ideal prediction. Most data points lie close to the ideal-prediction line, indicating strong agreement between the predicted and reference values, with R^2 values very close to 1.

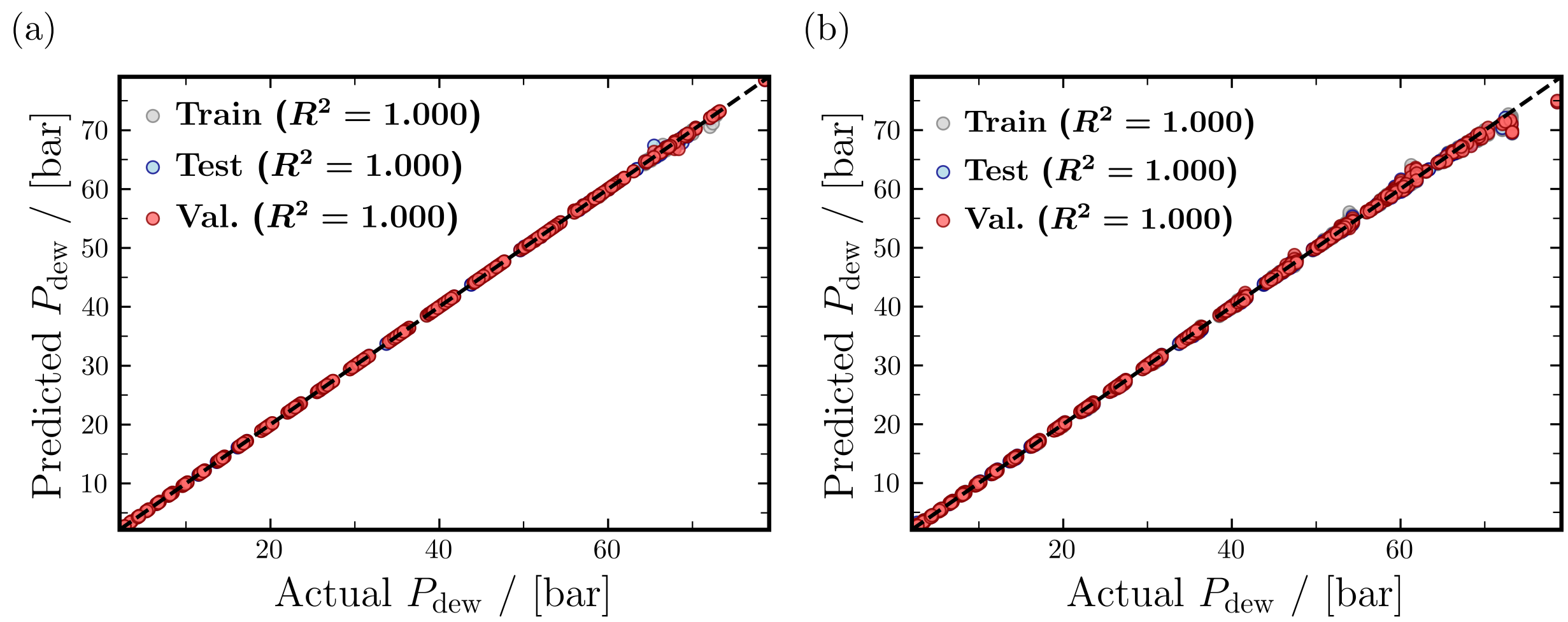

Figure S13: Parity plots for P_{dew} obtained from (a) RF, (b) SVR, (c) XGB, and (d) TabPFN. The training, test, and validation sets are presented separately to evaluate both fitting and predictive performance on untrained data. The R^2 values reported in each subplot quantify the agreement between the predicted and reference values. The diagonal line represents the ideal prediction. Most data points lie close to the ideal-prediction line, indicating strong agreement between the predicted and reference values, with R^2 values very close to 1.

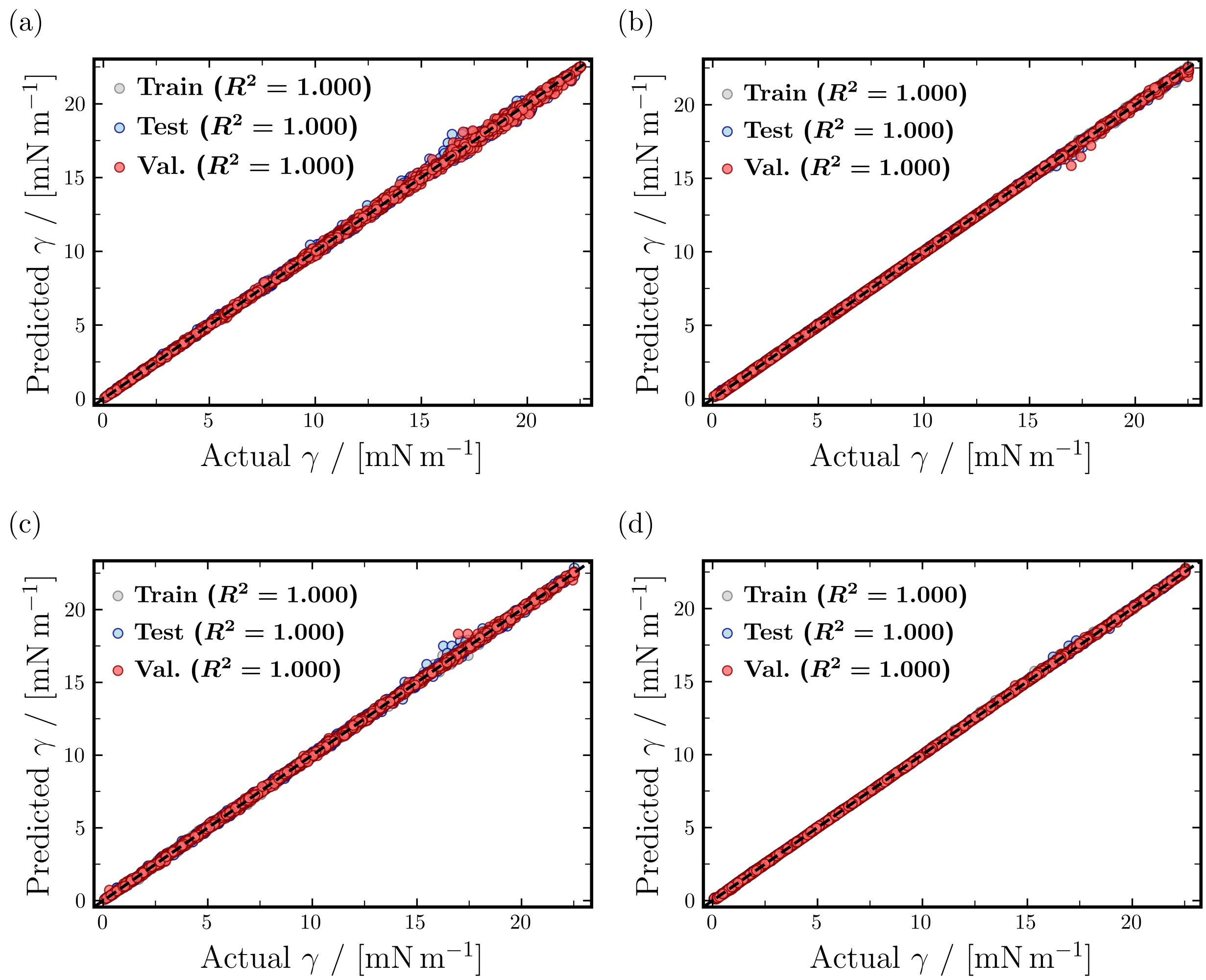

Figure S14: Parity plots for γ obtained from (a) RF, (b) SVR, (c) XGB, and (d) TabPFN. The training, test, and validation sets are presented separately to evaluate both fitting and predictive performance on untrained data. The R^2 values reported in each subplot quantify the agreement between the predicted and reference values. The diagonal line represents the ideal prediction. Most data points lie close to the ideal-prediction line, indicating strong agreement between the predicted and reference values, with R^2 values very close to 1.

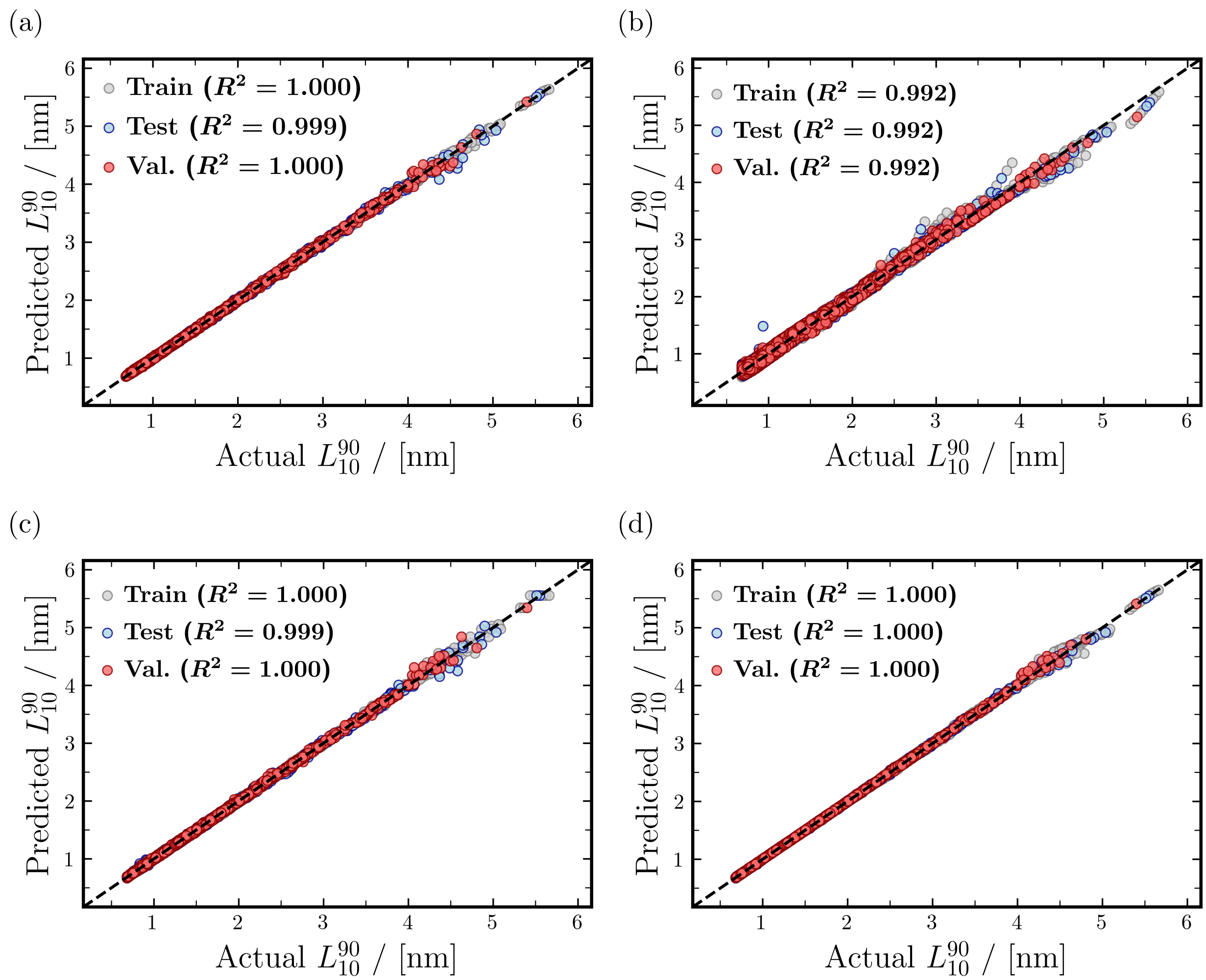

Figure S15: Parity plots for interfacial thickness, L_{10}^{90} obtained from (a) RF, (b) SVR, (c) XGB, and (d) TabPFN. The training, test, and validation sets are presented separately to evaluate both fitting and predictive performance on untrained data. The R^2 values reported in each subplot quantify the agreement between the predicted and reference values. The diagonal line represents the ideal prediction. Most data points lie close to the ideal-prediction line, indicating strong agreement between the predicted and reference values, with R^2 values very close to 1.

S4.2 Hyperparameter selection

Table S4 lists the candidate values considered for each hyperparameter and the corresponding selected configuration for each model. The same configuration was used for all four target properties (P_{bubble}, P_{dew}, γ, and interfacial thickness). For RF[45] and SVR,[46,47] each hyperparameter was swept individually for the grid shown in Table S4, and the value minimizing the test RMSE was retained. For XGB,[48,49] the hyperparameters were swept sequentially, with each one optimized in turn while the others were held at their previously-selected values. For TabPFN,[50] Bayesian optimization with 100 trials per target (Optuna[52]) was performed using the TabPFN-extensions package.[53] The RF and SVR models were implemented in scikit-learn,[54] and XGB in the XGBoost Python package.[49] The corresponding RMSE sweep curves are shown in Figs. S16 and S17 (RF), Figs. S18 to S21 (SVR), and Figs. S22 to S25 (XGB). The TabPFN[50,51,53] selected hyperparameters are listed in Table S5.

Table S4: Candidate hyperparameter values and selected values for each ML model. All four target properties used the same configuration.

Model	Hyperparameter	Candidate values	Selected
RF	No. trees (n_{trees})	10, 25, 50, 75, 100, 150, 200, 300, 400, 500	200
	Max. tree depth	1, 2, 3, 4, 5, 6, 8, 10, None	None
SVR	Regularization (C)	0.01, 0.1, 1, 10, 100, 500, 1000, 5000	100
	Loss bandwidth (ε)	0.001, 0.005, 0.01, 0.05, 0.1, 0.5, 1.0, 2.0	0.1
XGB	No. boosting rounds (n_{trees})	50, 100, 200, 300, 500, 750, 1000, 1500	500
	Max. tree depth	2, 3, 4, 5, 6, 7, 8, 10	6
	Learning rate (η)	0.001, 0.005, 0.01, 0.05, 0.1, 0.2, 0.3, 0.5	0.05
TabPFN	Bayesian optimization (Optuna), 100 trials per target; see Table S5		

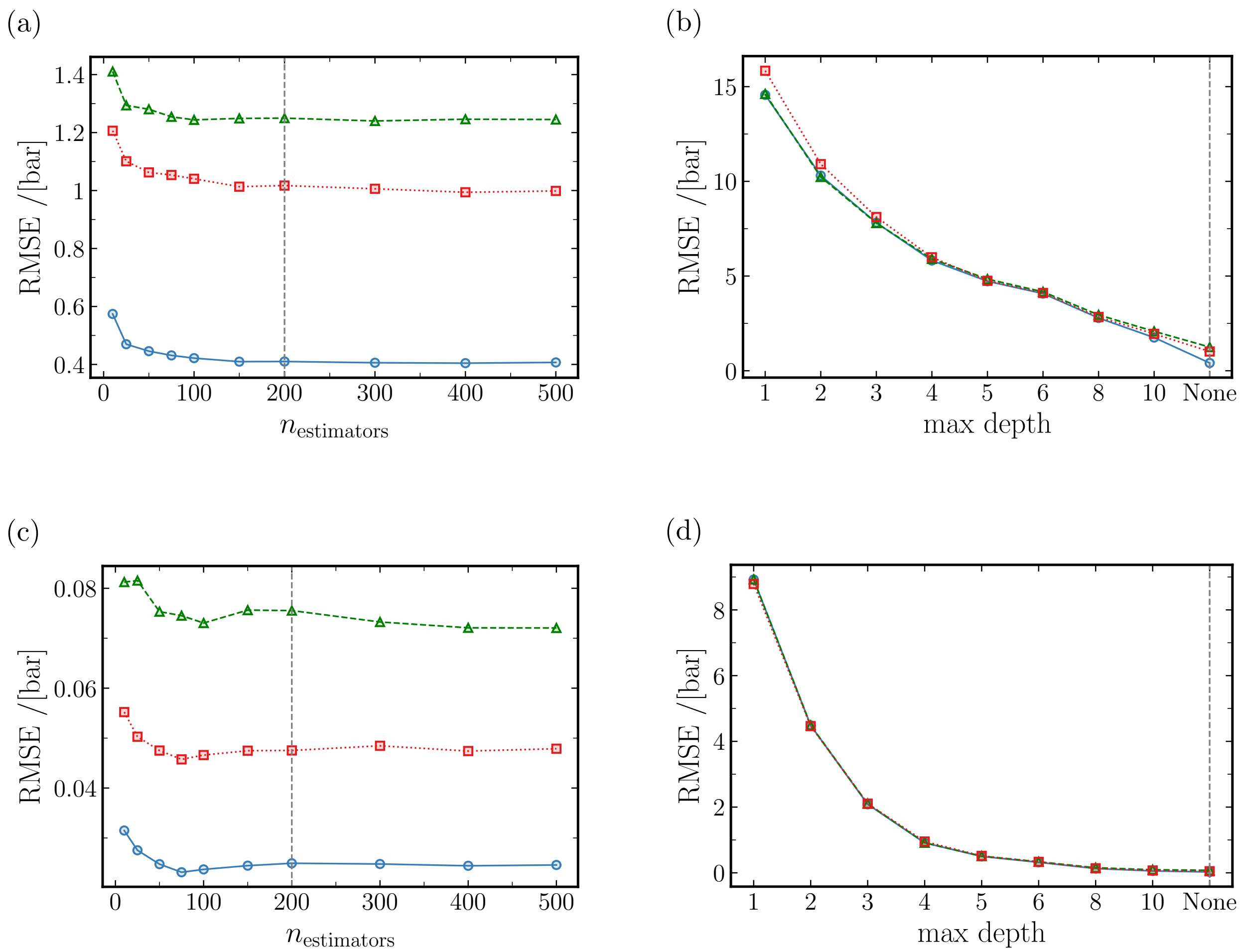

Figure S16: Root Mean Square Error (RMSE) of the training, cross-validation, and test sets as a function of the number of trees (left column) and maximum tree depth (right column) for the Random forest (RF)[45] model. Rows correspond to P_{bubble} (a,b) and P_{dew} (c,d). Selected values: $n_{\text{trees}} = 200$ and unlimited tree depth (`max_depth=None`), where nodes are expanded until all leaves are pure. In each panel, the training, cross-validation, and test RMSE are shown as blue circles with a solid line, green triangles with a dashed line, and red squares with a dotted line, respectively, while the vertical gray dashed line marks the selected hyperparameter value adopted for the RF model.

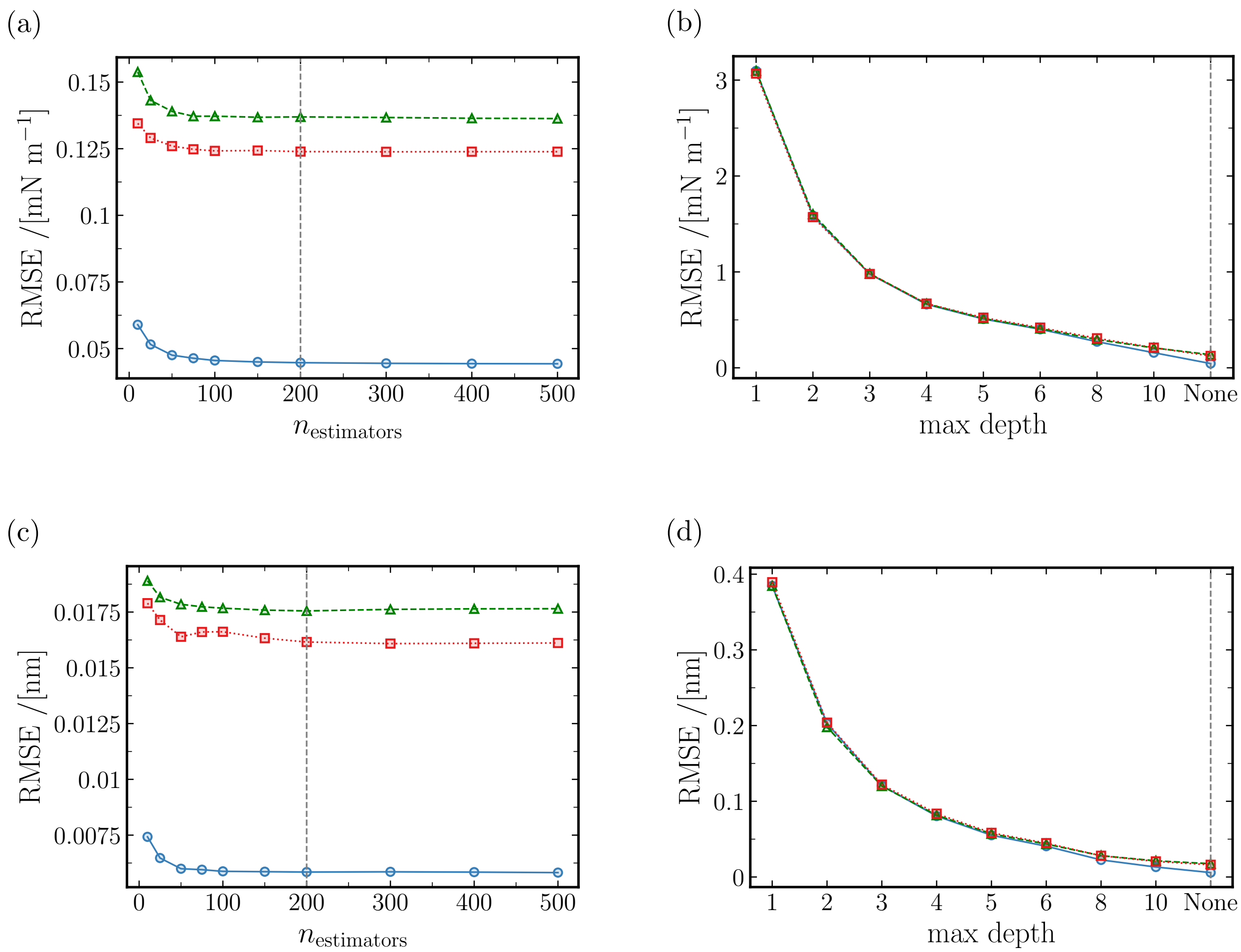

Figure S17: Root Mean Square Error (RMSE) on the training, cross-validation, and test sets as a function of the number of trees (left column) and maximum tree depth (right column) for the Random forest (RF)[45] model. Rows correspond to γ (a,b) and interfacial thickness (c,d). Selected values: $n_{\text{trees}} = 200$ and unlimited tree depth (`max_depth=None`), where nodes are expanded until all leaves are pure. In each panel, the training, cross-validation, and test RMSE are shown as blue circles with a solid line, green triangles with a dashed line, and red squares with a dotted line, respectively, while the vertical gray dashed line marks the selected hyperparameter value adopted for the RF model.

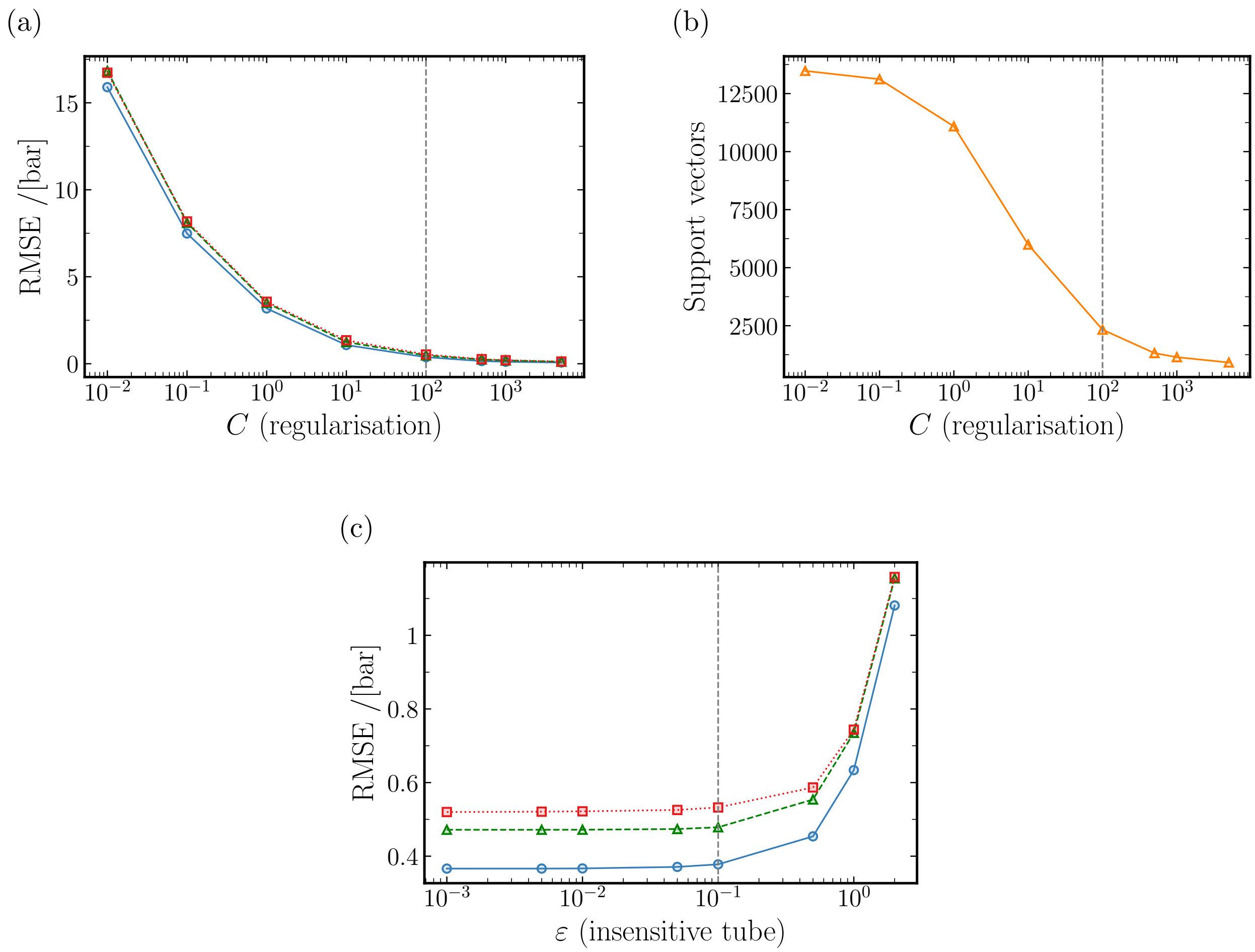

Figure S18: Hyperparameter sensitivity of the Radial Basis Function (RBF)-kernel Support Vector Regression (SVR)[47] model for P_{bubble}. Panel (a) shows the Root Mean Square Error (RMSE) for the training, cross-validation, and test sets as a function of the regularization parameter C, while panel (b) shows the corresponding number of support vectors. Panel (c) reports the RMSE as a function of the ε-insensitive loss bandwidth. The selected hyperparameters are $C = 100$ and $\varepsilon = 0.1$. In panels (a) and (c), the training, cross-validation, and test RMSE are shown as blue circles with a solid line, green triangles with a dashed line, and red squares with a dotted line, respectively, while panel (b) shows the number of support vectors as orange triangles with a solid line. In all panels, the vertical gray dashed line marks the selected hyperparameter value, namely C in panels (a) and (b) and ε in panel (c).

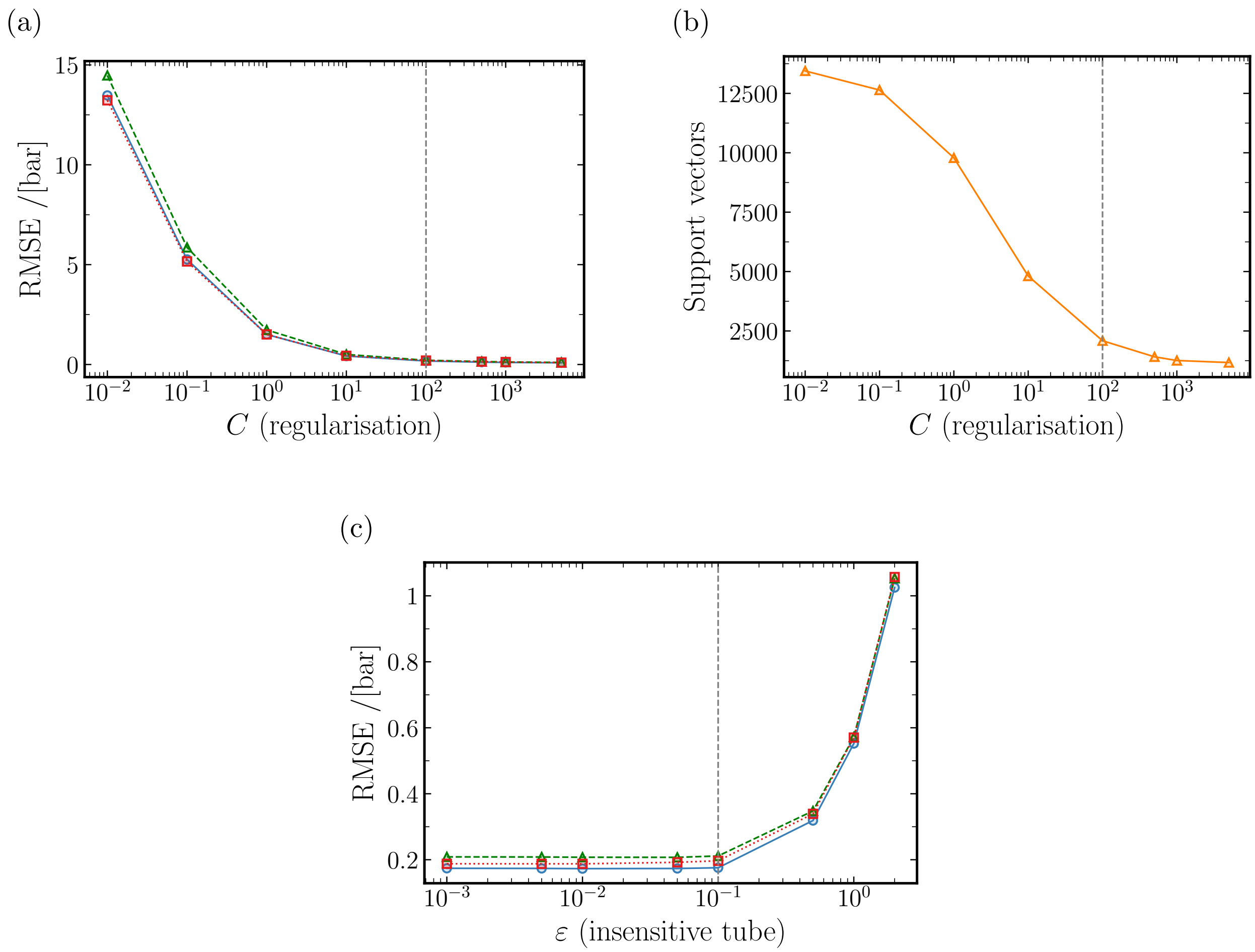

Figure S19: Hyperparameter sensitivity of the Radial Basis Function (RBF)-kernel Support Vector Regression (SVR)[47] model for P_{dew}. Panel (a) shows the Root Mean Square Error (RMSE) for the training, cross-validation, and test sets as a function of the regularization parameter C, while panel (b) shows the corresponding number of support vectors. Panel (c) reports the RMSE as a function of the ε-insensitive loss bandwidth. The selected hyperparameters are $C = 100$ and $\varepsilon = 0.1$. In panels (a) and (c), the training, cross-validation, and test RMSE are shown as blue circles with a solid line, green triangles with a dashed line, and red squares with a dotted line, respectively, while panel (b) shows the number of support vectors as orange triangles with a solid line. In all panels, the vertical gray dashed line marks the selected hyperparameter value, namely C in panels (a) and (b) and ε in panel (c).

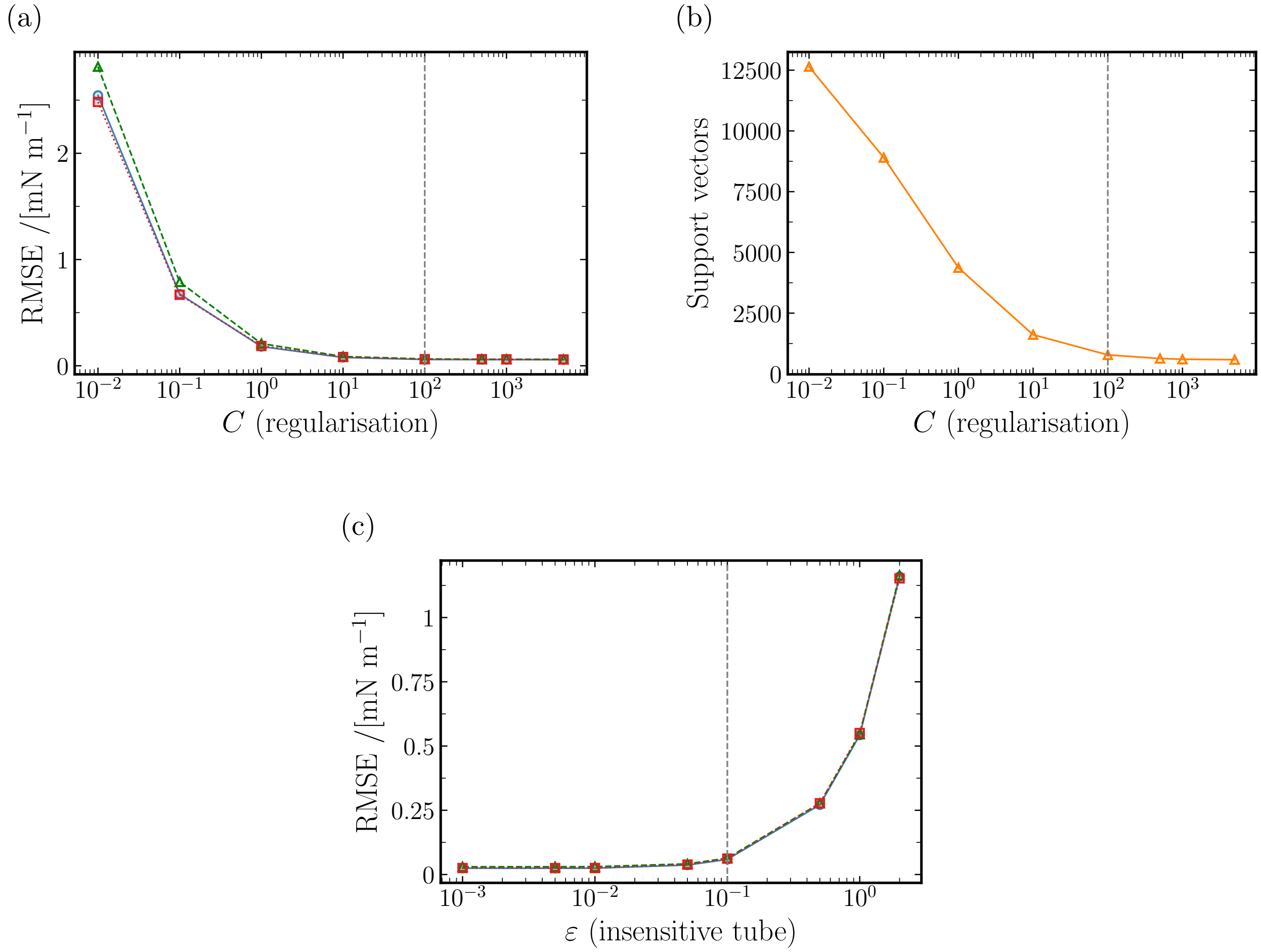

Figure S20: Hyperparameter sensitivity of the Radial Basis Function (RBF)-kernel Support Vector Regression (SVR)[47] model for γ. Panel (a) shows the Root Mean Square Error (RMSE) for the training, cross-validation, and test sets as a function of the regularization parameter C, while panel (b) shows the corresponding number of support vectors. Panel (c) reports the RMSE as a function of the ε-insensitive loss bandwidth. The selected hyperparameters are $C = 100$ and $\varepsilon = 0.1$. In panels (a) and (c), the training, cross-validation, and test RMSE are shown as blue circles with a solid line, green triangles with a dashed line, and red squares with a dotted line, respectively, while panel (b) shows the number of support vectors as orange triangles with a solid line. In all panels, the vertical gray dashed line marks the selected hyperparameter value, namely C in panels (a) and (b) and ε in panel (c).

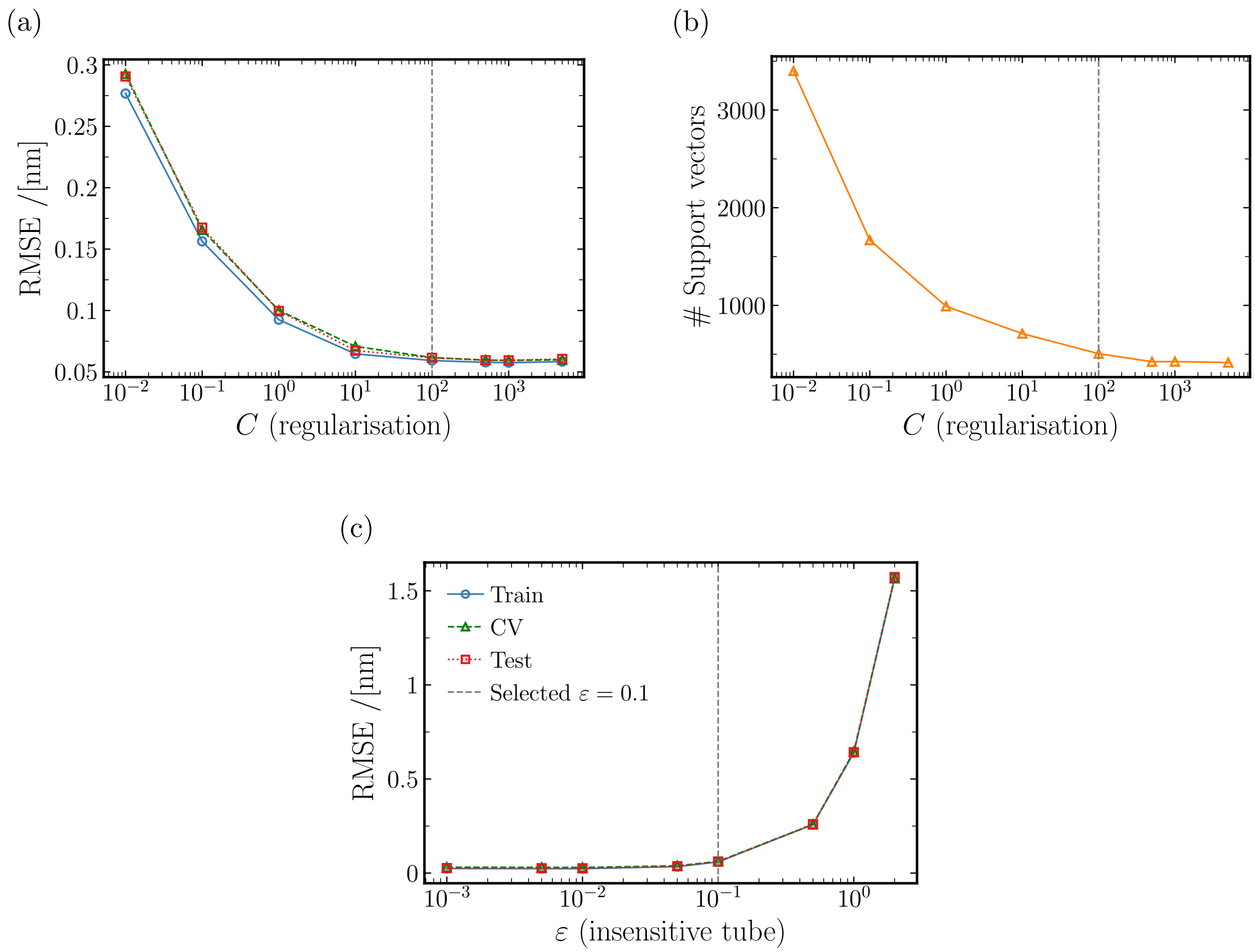

Figure S21: Hyperparameter sensitivity of the Radial Basis Function (RBF)-kernel Support Vector Regression (SVR)[47] model for interfacial thickness. Panel (a) shows the Root Mean Square Error (RMSE) for the training, cross-validation, and test sets as a function of the regularization parameter C, while panel (b) shows the corresponding number of support vectors. Panel (c) reports the RMSE as a function of the ε-insensitive loss bandwidth. The selected hyperparameters are $C = 100$ and $\varepsilon = 0.1$. In panels (a) and (c), the training, cross-validation, and test RMSE are shown as blue circles with a solid line, green triangles with a dashed line, and red squares with a dotted line, respectively, while panel (b) shows the number of support vectors as orange triangles with a solid line. In all panels, the vertical gray dashed line marks the selected hyperparameter value, namely C in panels (a) and (b) and ε in panel (c).

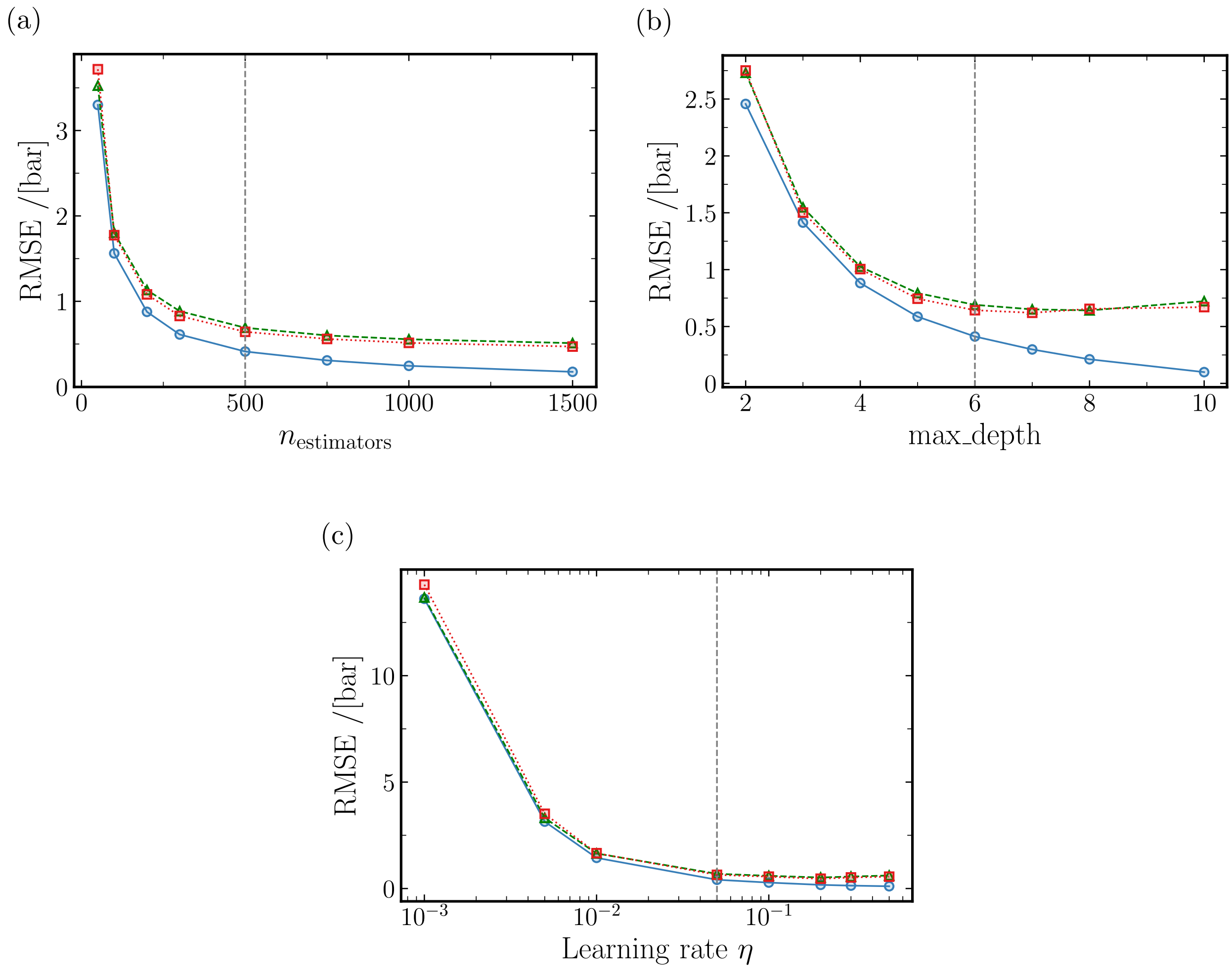

Figure S22: Hyperparameter sensitivity of the eXtreme Gradient Boosting (XGB) [48,49] model for P_{bubble}. Panel (a) shows the Root Mean Square Error (RMSE) for the training, cross-validation, and test sets as a function of the number of estimators, $n_{\mathrm{estimators}}$, while panel (b) shows the corresponding sensitivity to the maximum tree depth. Panel (c) reports the RMSE as a function of the learning rate, η. In each sweep, the remaining hyperparameters were fixed at their selected values: $n_{\mathrm{trees}} = 500$, max depth $= 6$, and $\eta = 0.05$. In each panel, the training, cross-validation, and test RMSE are shown as blue circles with a solid line, green triangles with a dashed line, and red squares with a dotted line, respectively, while the vertical gray dashed line marks the selected hyperparameter value adopted for the final model.

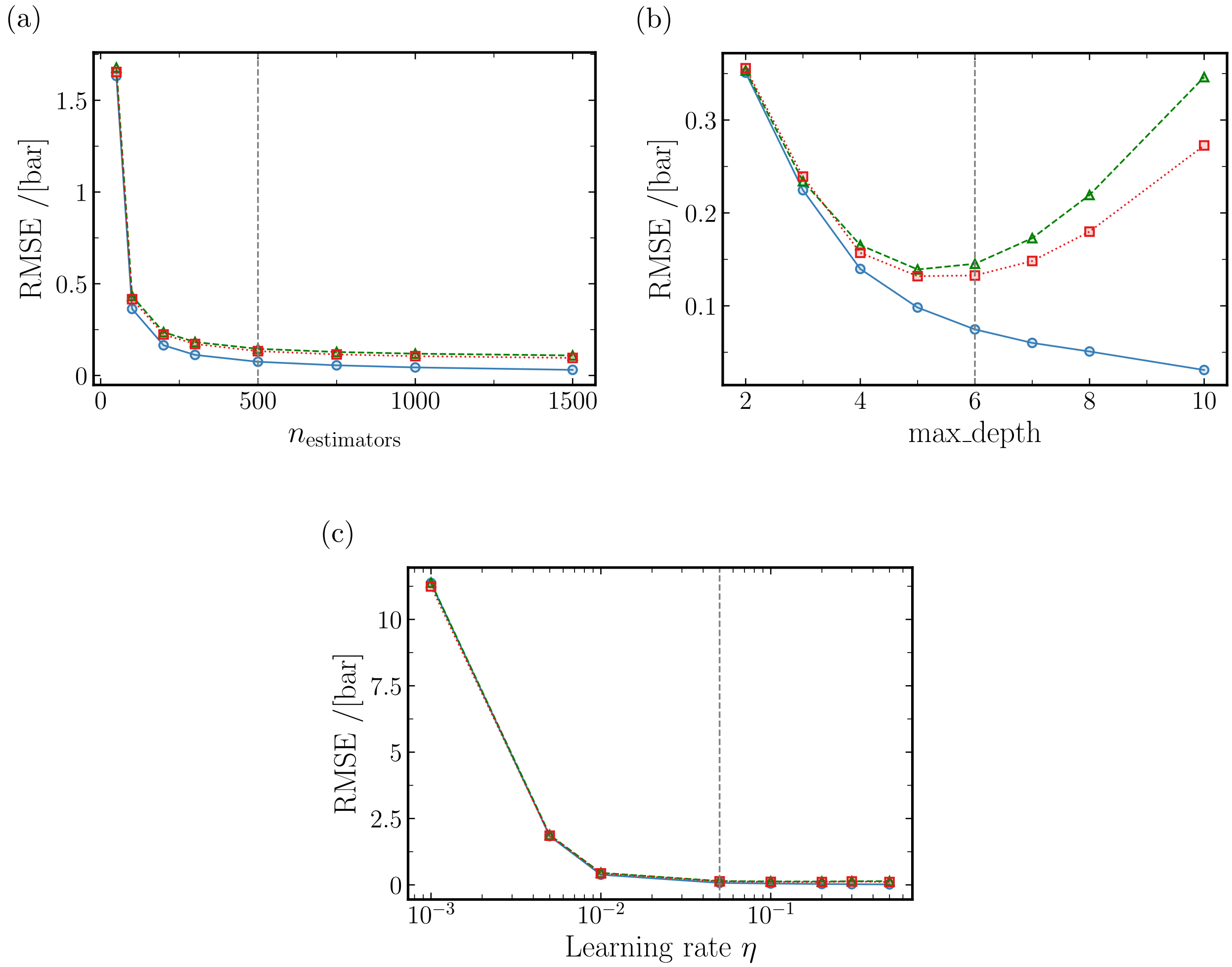

Figure S23: Hyperparameter sensitivity of the eXtreme Gradient Boosting (XGB)[48,49] model for P_{dew}. Panel (a) shows the Root Mean Square Error (RMSE) for the training, cross-validation, and test sets as a function of the number of estimators, $n_{\text{estimators}}$, while panel (b) shows the corresponding sensitivity to the maximum tree depth. Panel (c) reports the RMSE as a function of the learning rate, η. In each sweep, the remaining hyperparameters were fixed at their selected values: $n_{\text{trees}} = 500$, max depth $= 6$, and $\eta = 0.05$. In each panel, the training, cross-validation, and test RMSE are shown as blue circles with a solid line, green triangles with a dashed line, and red squares with a dotted line, respectively, while the vertical gray dashed line marks the selected hyperparameter value adopted for the final model.

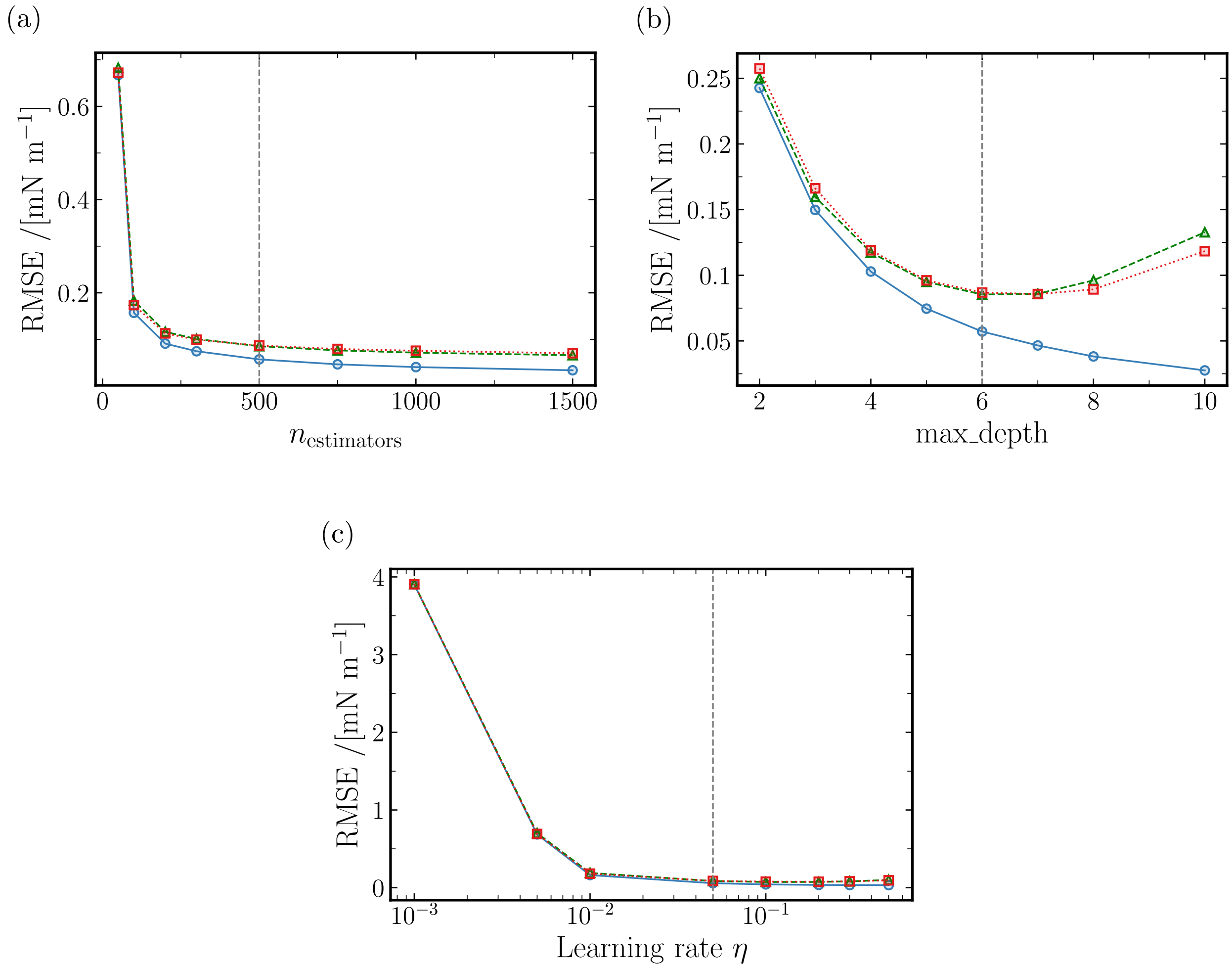

Figure S24: Hyperparameter sensitivity of the eXtreme Gradient Boosting (XGB) [48,49] model for γ. Panel (a) shows the Root Mean Square Error (RMSE) for the training, cross-validation, and test sets as a function of the number of estimators, $n_{\mathrm{estimators}}$, while panel (b) shows the corresponding sensitivity to the maximum tree depth. Panel (c) reports the RMSE as a function of the learning rate, η. In each sweep, the remaining hyperparameters were fixed at their selected values: $n_{\mathrm{trees}} = 500$, $\max\mathrm{depth} = 6$, and $\eta = 0.05$. In each panel, the training, cross-validation, and test RMSE are shown as blue circles with a solid line, green triangles with a dashed line, and red squares with a dotted line, respectively, while the vertical gray dashed line marks the selected hyperparameter value adopted for the final model.

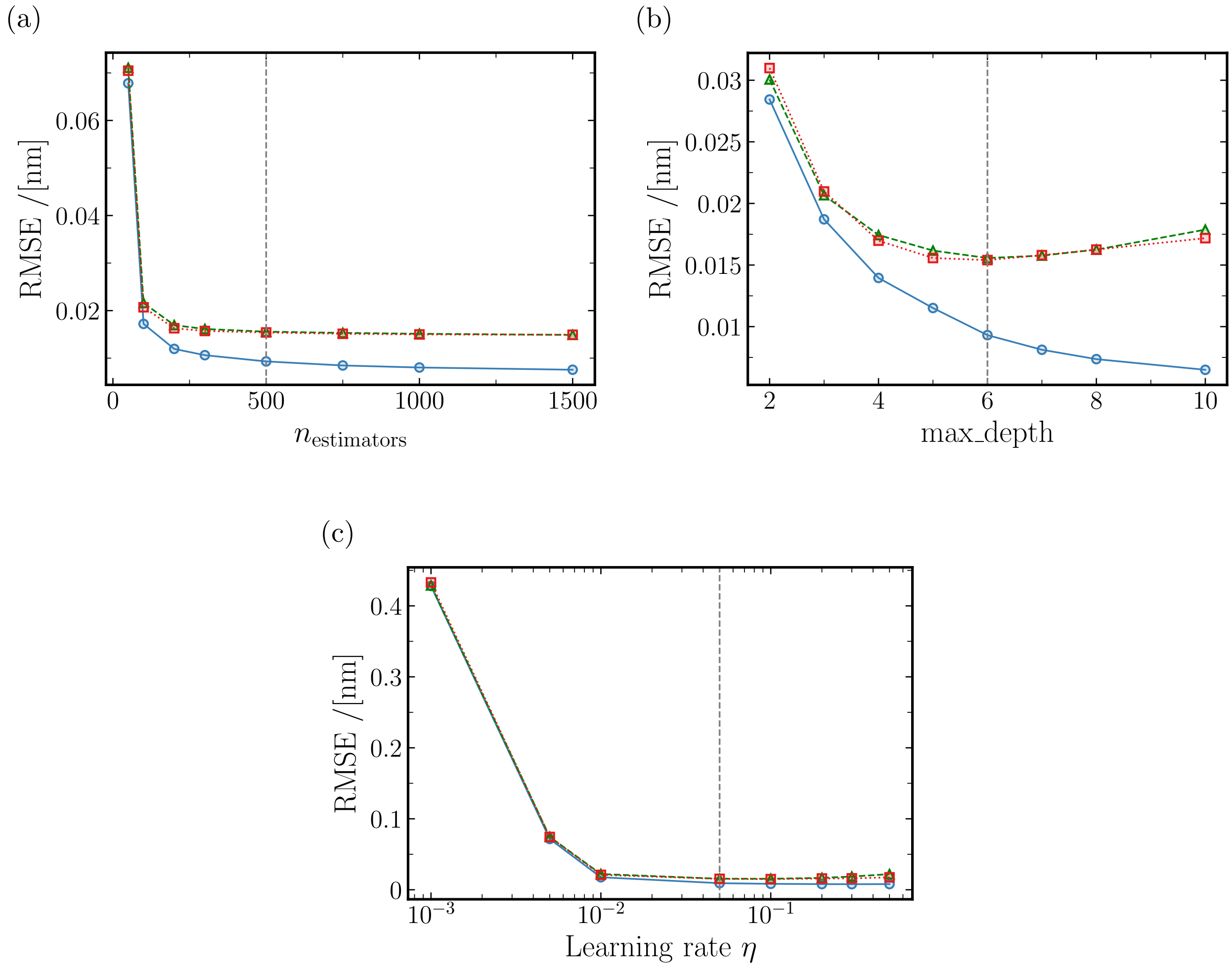

Figure S25: Hyperparameter sensitivity of the eXtreme Gradient Boosting (XGB) model for interfacial thickness. Panel (a) shows the Root Mean Square Error (RMSE) for the training, cross-validation, and test sets as a function of the number of estimators, $n_{\mathrm{estimators}}$, while panel (b) shows the corresponding sensitivity to the maximum tree depth. Panel (c) reports the RMSE as a function of the learning rate, η. In each sweep, the remaining hyperparameters were fixed at their selected values: $n_{\mathrm{trees}} = 500$, max depth $= 6$, and $\eta = 0.05$. In each panel, the training, cross-validation, and test RMSE are shown as blue circles with a solid line, green triangles with a dashed line, and red squares with a dotted line, respectively, while the vertical gray dashed line marks the selected hyperparameter value adopted for the final model.

Table S5: Tabular Prior-data Fitted Network (TabPFN) (v2.5)[50,51,53] hyperparameter search space and Optuna[52]-selected configurations for each target property, using 100 trials per target. The default column reports the package default settings, the search space column lists the candidate values explored during optimization, and each target-property column lists the configuration selected by Optuna for that specific property. The target properties are P_{bubble}, P_{dew}, γ, and interfacial thickness. Abbreviations: SP = safepower transform; Quant = quantile-uniform transform, with Quant-norm denoting the normal-output variant; Squash = squashing-scaler transform; KDI = kernel density integral transform,[55] with KDI-uni denoting the uniform-output variant; SVD = global singular-value-decomposition projection applied after the feature transform; id. = identity transform, i.e., no preprocessing transform; None = no feature transform; and † = original features appended after the transformed features. The `dt_pfn` architecture denotes the decision-tree TabPFN variant provided by the TabPFN-extensions package.[53] The Feature preprocessing row was selected from 180 candidate transform combinations.

**Parameter**	**Default**	**Search space**	$\boldsymbol{P_{\mathrm{bubble}}}$	$\boldsymbol{P_{\mathrm{dew}}}$	$\boldsymbol{\gamma}$	**Interfacial Thickness**
Architecture	single	{single, dt_pfn}	single	dt_pfn	dt_pfn	single
Pre-trained checkpoint	default	default, low-skew, quantiles, real-variant, real, small-samples, variant	low-skew	small-samples	small-samples	small-samples
Ensemble size (n_{pred})	8	{4}	4	4	4	4
Tree depth (dt_pfn)	—	{2, 3, 4, 5}	—	4	2	—
Average logits	No	{Yes, No}	Yes	Yes	No	Yes
Softmax temperature	0.9	{0.75, 0.80, 0.90, 0.95, 1.00, 1.05}	0.80	0.75	0.90	0.90
Fingerprint feature	Yes	{Yes, No}	No	No	No	Yes
Feature preprocessing	Quant+SVD	180 combos	None+SVD†	Quant-norm†	None+SVD†	Squash+SVD†
Polynomial features	No	{No}	No	No	No	No
Outlier removal (σ_{max})	∞	{∞, 7.0, 12.0}	12.0	∞	∞	12.0
Min unique for numerical	1	{1, 5, 10, 30}	10	1	1	10
Target preprocessing	{id., SP}	{id., id.+SP, SP}	id.+SP	id.	id.+SP	SP

S4.3 Prediction intervals

TabPFN[50] predicts a distribution of possible values for the target property rather than a single predicted value, so the same forward pass also provides a Prediction Interval (PI) at no additional computational cost. Figure S26 shows the central 95% PI, bounded by the 2.5% and 97.5% quantiles, of the hyperparameter-optimized TabPFN model for P_{bubble}, evaluated without further calibration at the 61 held-out state points of feed 2 of Table 2 of the main text. The width varies by more than an order of magnitude, from ca. 0.6–1 bar for $T > 240$ K to ca. 20 bar at 200 K, widening where the residuals reported in Figure 4d of the main text are largest. The intervals are conservative. Every reference value lies inside the 95% PI, both at the 61 held-out points of this feed and at all 5808 test and validation points of the dataset. The median interval width over all test and validation points is 0.34 bar. The width therefore flags the low-temperature, H_2-rich states at which a surrogate prediction should be verified using a direct PCP-SAFT EoS + cDFT computation. This readout is available only for P_{bubble} and L_{10}^{90}, because for P_{dew} and γ the optimization selected the decision-tree architecture,[53] whose output has no model-defined quantiles.

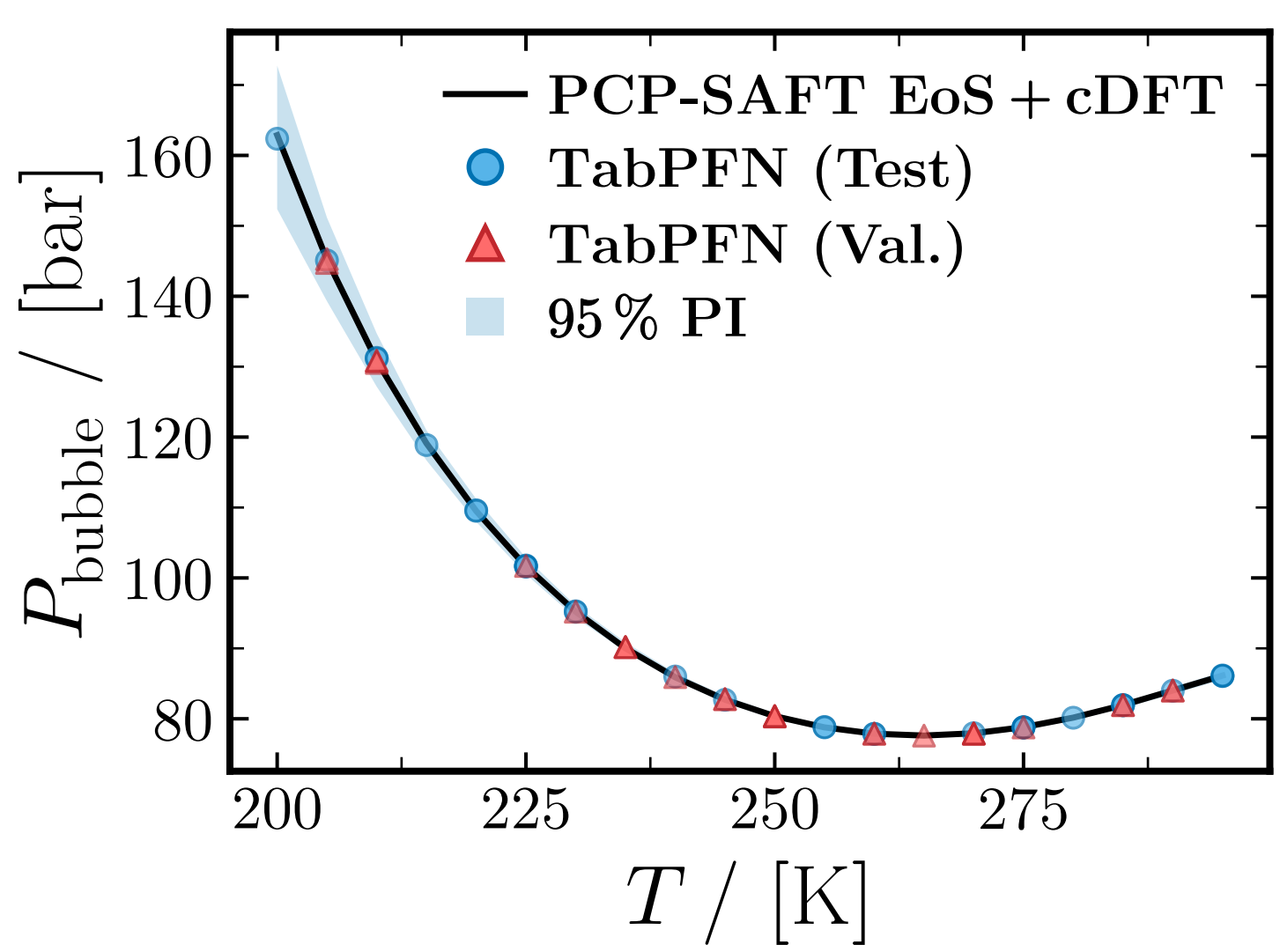

Figure S26: Central 95% Prediction Interval (PI) of the hyperparameter-optimized TabPFN[50] model for P_{bubble}. Predictions are shown at the 61 held-out state points of feed 2 listed in Table 2 of the main text, the feed with the largest H_2 content. The interval is bounded by the 2.5% and 97.5% quantiles of the predicted distribution.

S4.4 Data and model availability

The datasets and trained models used in this work are included in the accompanying supplementary material in the folder `MACHINE_LEARNING/`. The subfolder `DATASET/` contains two comma-separated files. The file `dataset_data_driven.csv` contains the input features and target properties used to train the data-driven models, namely RF, SVR, XGB, and TabPFN, for P_{bubble}, P_{dew}, γ, and the interfacial thickness L_{10}^{90}. The file `dataset_wsd_residual.csv` additionally contains the WSD baseline predictions and the corresponding cDFT–WSD residual columns used to train the hybrid residual-correction models, namely SR, Gaussian Process Regression (GPR), and Stochastic Variational Gaussian Process Regression (SVGP). The training, validation, and test splits are generated directly from these files using a fixed random seed. The subfolder `TabPFN/` provides, for each target property, the tuned TabPFN model saved as a `⟨TARGET_PROPERTY⟩_model.joblib` file and the corresponding train, test, and validation set predictions saved as `⟨TARGET_PROPERTY⟩_predictions.csv`. The optimized hyperparameters are reported in Table S5.

S5 Symbolic Regression (SR) of the interfacial tension (IFT)

We present the Symbolic Regression (SR)-based correlations used to compute the pure-component interfacial tension (IFT) as a function of temperature, $\gamma(T)$ in Section S5.1. We also present the hybrid ML + WSD residual correction obtained for multi-component CO_2-rich mixtures in Section S5.2.

S5.1 Pure-component interfacial tension (IFT) correlations

The IFTs of pure fluids are commonly represented by the semi-empirical Guggenheim-Katayama (GK) equation[56,57]

$$\gamma(T) = \sum_{i=0}^{k-1} s_i \left(1 - \frac{T}{T_c}\right)^{n_i}, \tag{S3}$$

where k is the number of terms, s_i and n_i are fluid-specific fit parameters, and T_c is the critical temperature.[58,59] This is the same functional form used in REFPROP v10[56] to compute interfacial tension (IFT) of pure components. The reference fit GK parameters used for comparison with the SR-based correlations in this work are reported in Table S6. For CO_2, the fit parameters were adopted from Cachadiña et al. (2015),[58] whereas for the impurity components, the fit parameters were adopted from Mulero et al. (2012).[59] Symbolic Regression (SR)[60] was used to obtain IFT correlations for eight pure components considered in this work. For each pure component, the reference IFTs were computed using the PCP-SAFT EoS+cDFT framework[1,7,61] for the corresponding two-phase temperature range and used as training targets. The reduced temperature, $T_r = T/T_c$, was used as the input feature. At low complexity, the SR search retrieves the canonical GK[57] single-term power-law form, $\gamma(T) = s(1 - T_r)^n$. The SR obtained coefficients for this form are reported in Table S7 to enable direct comparison with the parameters in Table S6. Another expres-

sion was selected from the Pareto front by balancing prediction accuracy with algebraic complexity and achieving a low Root Mean Square Error (RMSE). In most cases, the selected expression is a higher-complexity, non-canonical refinement of the same functional form. These selected correlations are provided in Eqs. (S4) to (S11), where γ is expressed in $\mathrm{mN\,m^{-1}}$. Figs. S27 to S30 show the temperature dependence of the pure-component IFT for each component considered in this work. These figures compare the reference fit parameters reported in Table S6 with the PCP-SAFT EoS + cDFT data used as the training target for SR. In addition, Figs. S27 to S30 also include two SR-based correlations: the canonical form, $\gamma(T) = s(1 - T_r)^n$, and a higher-complexity non-canonical expression (Eqs. (S4) to (S11)). Both SR correlations closely reproduce the PCP-SAFT+cDFT [1,5,7] reference data for the two-phase temperature range and are visually almost indistinguishable from the reference IFT curves. In contrast, the semi-empirical GK correlations parameterized by Müller et al. [62] and Cachadiña et al.,[58] which were fitted to experimental data, show small systematic deviations from the PCP-SAFT+cDFT values for CH_4, CO_2, N_2, CO, and H_2S, particularly at temperatures far from the critical point. This comparison supports the use of the PCP-SAFT EoS+cDFT parameter set listed in Table S1 for computing pure-component IFT. In addition, the selected SR equations provide correlations for the pure-component IFT, enabling direct evaluation of $\gamma(T)$ without performing cDFT calculations. Hydrogen is considered separately because, as a light quantum fluid, its interfacial properties are not adequately described by the PCP-SAFT EoS. As shown in Fig. S31a, the PCP-SAFT-based predictions of the H_2 IFTs deviate substantially from the quantum-corrected Statistical Associating Fluid Theory of Variable Range with Feynman–Hibbs quantum corrections for the Mie potential (SAFT-VRQ Mie) + cDFT reference data. Therefore, the SR correlation for H_2 was trained using the SAFT-VRQ Mie + cDFT data instead of the PCP-SAFT + cDFT data. For the SAFT-VRQ Mie description of H_2, the molecular parameters were taken from Aasen et al.,[63] who reported optimized SAFT-VRQ Mie parameters for hydrogen using first- and second-order Feynman–Hibbs quantum corrections.[63] In this work, the parameter set

corresponding to the second-order Feynman–Hibbs correction was used. It should be noted that these Feynman–Hibbs quantum corrections are important mainly near the critical point of hydrogen ($T_c \approx 33$ K), where the low molecular mass of H_2 makes quantum delocalization significant. At the temperatures relevant to the CO_2-rich mixtures studied here (approximately 200–300 K), which lie well above the critical temperature of hydrogen, these quantum corrections become negligible and are therefore not strictly necessary for the mixture IFT computations. Fig. S31c compares the resulting SR correlation with the reference IFT data. The selected SR correlation closely reproduces the SAFT-VRQ Mie + cDFT reference data throughout the subcritical temperature range of H_2. However, in the temperature range relevant to the multi-component CO_2-rich mixtures studied here, approximately 200–300 K, the PCP-SAFT EoS and the SAFT-VRQ Mie model predict nearly identical H_2 densities, as shown in Fig. S31b. Hence, the PCP-SAFT EoS was used for H_2 in the mixture IFT computations, even though the pure-component H_2 SR correlation was trained on SAFT-VRQ Mie+cDFT data. This was necessary because the multi-component cDFT computations must be formulated using a single underlying EoS. Therefore, SAFT-VRQ Mie for H_2 cannot be combined directly with PCP-SAFT for the remaining components within the same mixture framework.

The selected SR correlations for each pure component, obtained by fitting the PCP-SAFT EoS+cDFT reference IFT data (and, for H_2, the SAFT-VRQ Mie+cDFT data), with γ expressed in $\mathrm{mN\,m^{-1}}$ and the reduced temperature $T_r = T/T_c$, are

$$\gamma_{\mathrm{CO_2}} = \left(20.27\,(1-T_r)\right)^{1.489}\left((1-T_r)+0.08212\right)^{-0.2819}, \tag{S4}$$

$$\gamma_{\mathrm{H_2}} = 7.454\left[(1-T_r)^2\left((1-T_r)^{-0.7122}-0.06842\right)-0.0004126\right], \tag{S5}$$

$$\gamma_{\mathrm{Ar}} = 0.08752+\left(-0.1867+(1-T_r)\,(19.37-6.58\,(1-T_r))\right)^{1.342}, \tag{S6}$$

$$\gamma_{\mathrm{N_2}} = \left((1-T_r)-0.01236\right)^{1.332}\left(34.43-12.23\,(1-T_r)\right)^{1.033}+0.09414, \tag{S7}$$

$$\gamma_{\mathrm{CH_4}} = 0.07774 + \left((1-T_r)\left((1-T_r) - 2.886\right) - 7.113\right)^{1.35}, \tag{S8}$$

$$\gamma_{\mathrm{O_2}} = 35.01\,(1-T_r)\left((1-T_r) - 1.512 + 2.414\,(1-T_r)^{0.5035}\right), \tag{S9}$$

$$\gamma_{\mathrm{CO}} = \left(0.1407\,(1-T_r)^{-0.7208} + 0.07408\right)^{-2.127}, \tag{S10}$$

$$\gamma_{\mathrm{H_2S}} = 118.7\,(1-T_r)^{1.416}\left((1-T_r)^{(1-T_r)}\right)^{(1-T_r)}. \tag{S11}$$

All eight expressions retain the GK-type $(1 - T_r)$ dependence with additional low-order non-canonical terms, and their complexities and validation RMSEs are listed in Table S7.

Table S6: The Guggenheim-Katayama (GK)[57] fit parameters s_i and n_i used to compute the pure-component IFTs in REFPROP v10.[56] k is the number of summation terms and T_c is the critical temperature.

Component	k / [-]	T_c / [K]	s_i / [mN m^{-1}]	n_i / [-]	Reference
CO_2	2	304.21	84.588	1.2814	Cachadiña et al. (2015)[58]
			−25.372	3.9648	
N_2	1	126.192	28.980	1.2460	Mulero et al. (2012)[59]
Ar	1	150.687	37.000	1.2500	Mulero et al. (2012)[59]
H_2	3	33.145	−1416.500	0.6388	Mulero et al. (2012)[59]
			746.383	0.6598	
			675.625	0.6191	
CH_4	3	190.564	38.250	1.1910	Mulero et al. (2012)[59]
			−6.024	5.4220	
			−0.7065	0.6161	
O_2	1	154.581	38.430	1.2250	Mulero et al. (2012)[59]
CO	1	132.860	28.430	1.1480	Mulero et al. (2012)[59]
H_2S	1	373.100	78.557	1.2074	Mulero et al. (2012)[59]

Table S7: Symbolic Regression (SR)[60] pure-component interfacial tension (IFT) correlations. The canonical columns yield the single-term GK-form fit $s\,(1-T_r)^n$ recovered by SR (directly comparable to Table S6). The last two columns show the complexity and validation Root Mean Square Error (RMSE) (in $\mathrm{mN\,m^{-1}}$) of the higher-accuracy expression selected from the Pareto front (Eqs. (S4) to (S11)). T_c is the critical temperature of the pure component computed from the PCP-SAFT EoS.[1,5,7]

Component	T_c / [K]	Canonical SR fit $s\,(1-T_r)^n$			Selected SR equation	
		s / $[\mathrm{mN\,m^{-1}}]$	n / [-]	RMSE	complexity	RMSE
CO_2	302.71	93.33	1.304	0.0437	11	0.0002
H_2	33.43	7.06	1.270	0.0025	13	0.0001
Ar	150.29	39.19	1.247	0.0870	13	0.0009
N_2	126.85	29.70	1.253	0.0754	17	0.0004
CH_4	191.45	39.59	1.210	0.1445	13	0.0014
O_2	154.67	40.20	1.249	0.1133	13	0.0060
CO	133.26	31.30	1.289	0.0554	9	0.0006
H_2S	364.65	92.51	1.303	0.1000	11	0.0100

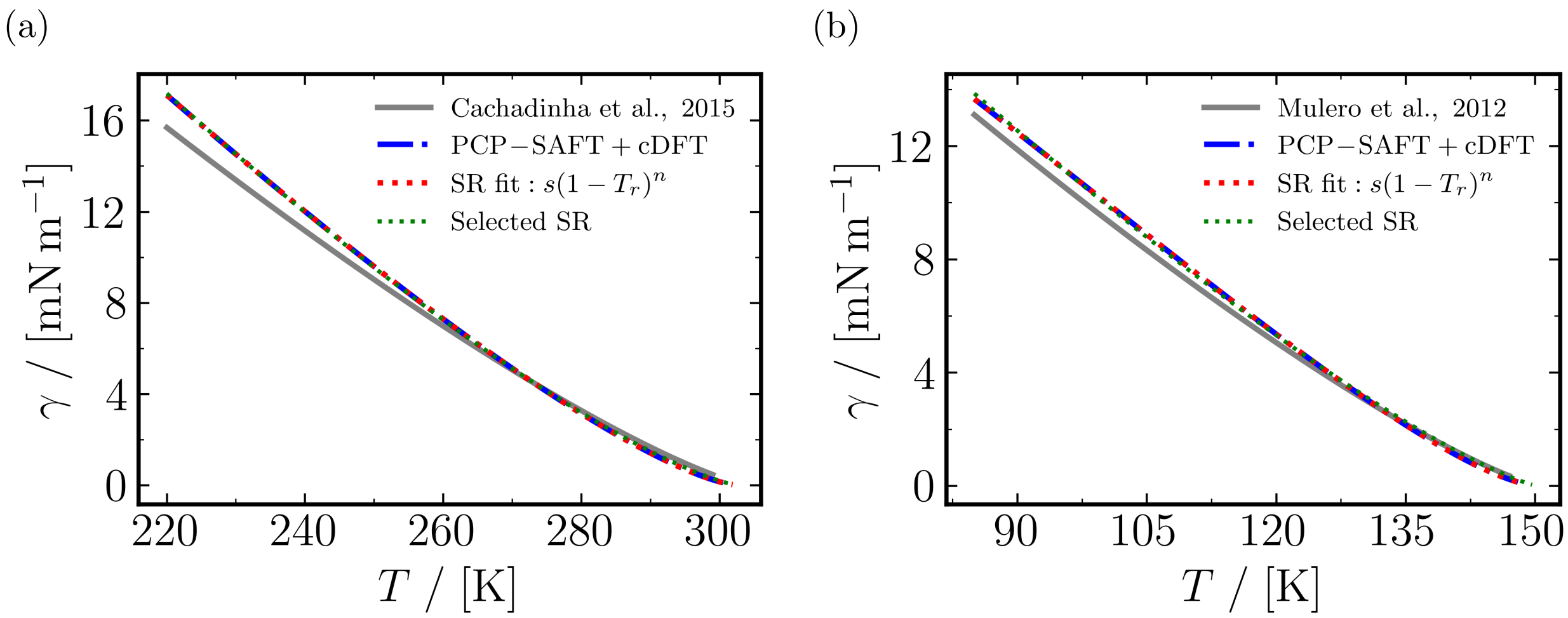

Figure S27: Pure-component interfacial tension (IFT), γ, for (a) CO_2 and (b) Ar. Panel (a) shows the Guggenheim-Katayama (GK) reference correlation,[57] parameterized by Cachadiña et al.,[58] compared with the PCP-SAFT EoS + cDFT[1,7,61] reference data used as the SR training target, the selected Symbolic Regression (SR)[60] correlation, and the lower-complexity canonical SR fit. Panel (b) shows the corresponding comparison for Ar, using the GK parameters reported by Mulero et al..[59] The complete GK parameters are listed in Table S6.

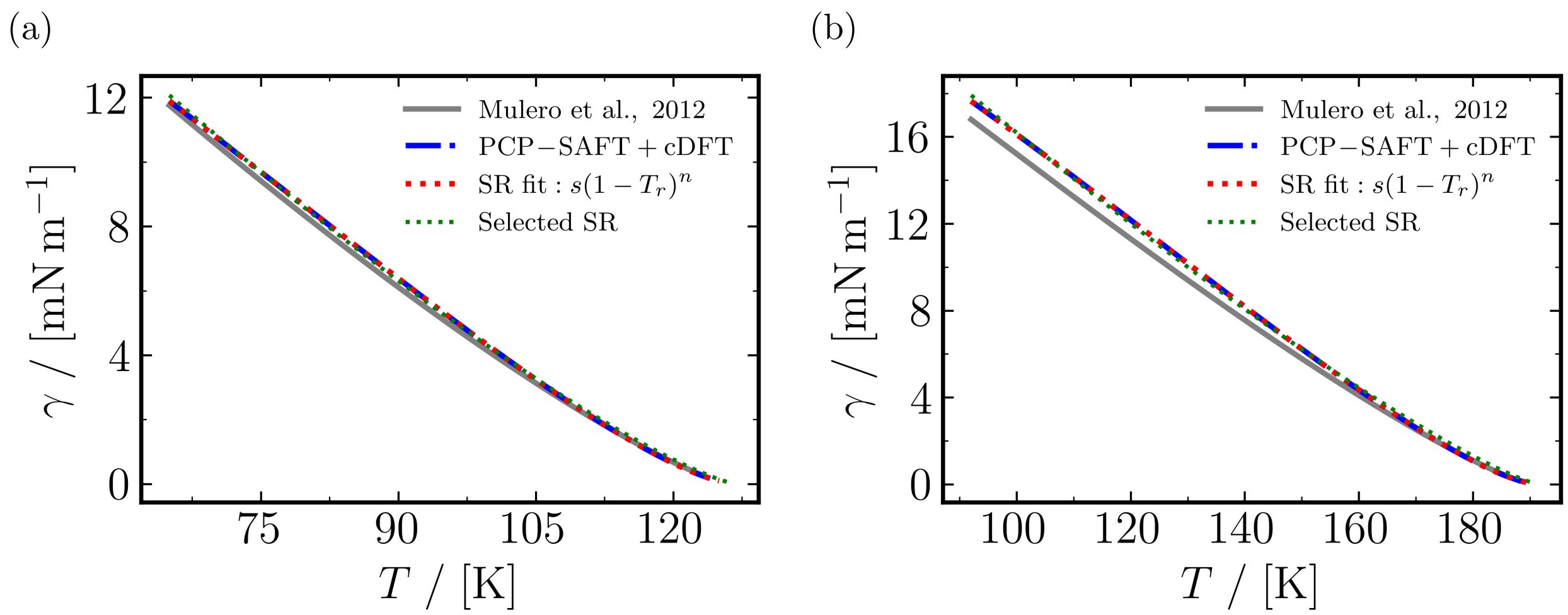

Figure S28: Pure-component interfacial tension (IFT), γ, for (a) N_2 and (b) CH_4. Panel (a) shows the Guggenheim-Katayama (GK) reference correlation,[57] parameterized using the fit parameters reported by Mulero et al.,[59] compared with the PCP-SAFT EoS + cDFT[1,7,61] reference data used as the SR training target, the selected Symbolic Regression (SR)[60] correlation, and the lower-complexity canonical SR fit for N_2. Panel (b) shows the corresponding comparison for CH_4. The complete GK parameters are listed in Table S6.

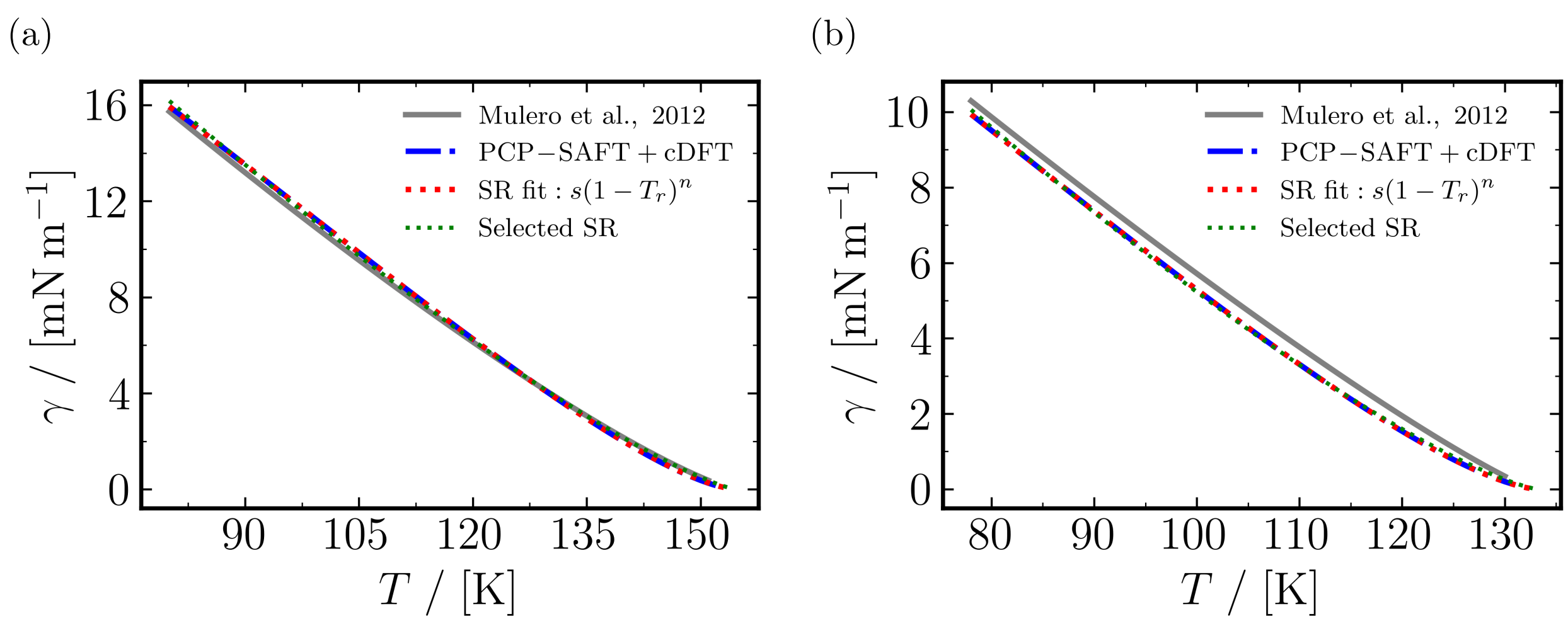

Figure S29: Pure-component interfacial tension (IFT), γ, for (a) O_2 and (b) CO. Panel (a) shows the Guggenheim-Katayama (GK) reference correlation,[57] parameterized using the fit parameters reported by Mulero et al.,[59] compared with the PCP-SAFT EoS + cDFT[1,7,61] reference data used as the SR training target, the selected Symbolic Regression (SR)[60] correlation, and the lower-complexity canonical SR fit for O_2. Panel (b) shows the corresponding comparison for CO. The complete GK parameters are listed in Table S6.

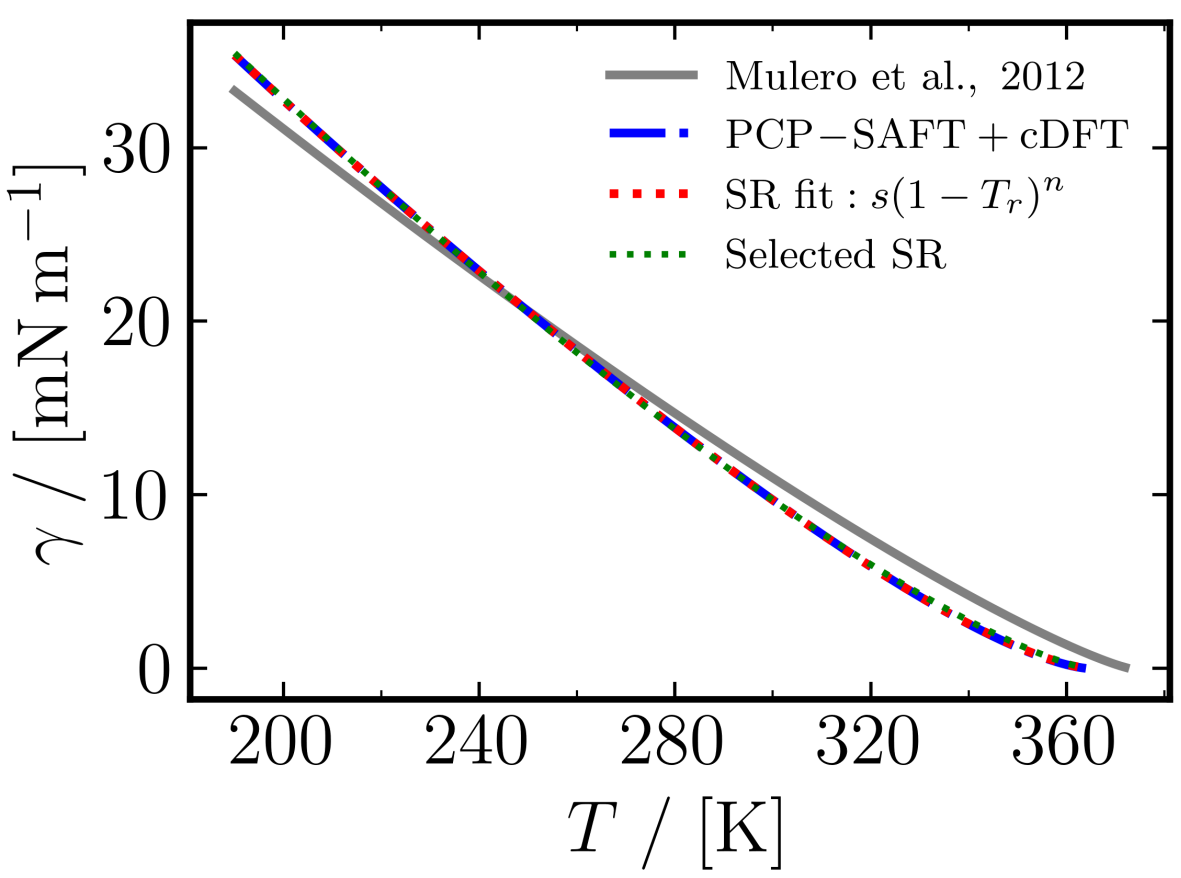

Figure S30: Pure-component interfacial tension (IFT), γ, for H_2S. The Guggenheim-Katayama (GK) reference correlation,[57] parameterized using the fit parameters listed in Table S6, is compared with the PCP-SAFT EoS + cDFT[1,7,61] reference data used as the SR training target, the selected Symbolic Regression (SR)[60] correlation, and the lower-complexity canonical SR fit. The GK parameters for H_2S are listed in Table S6.

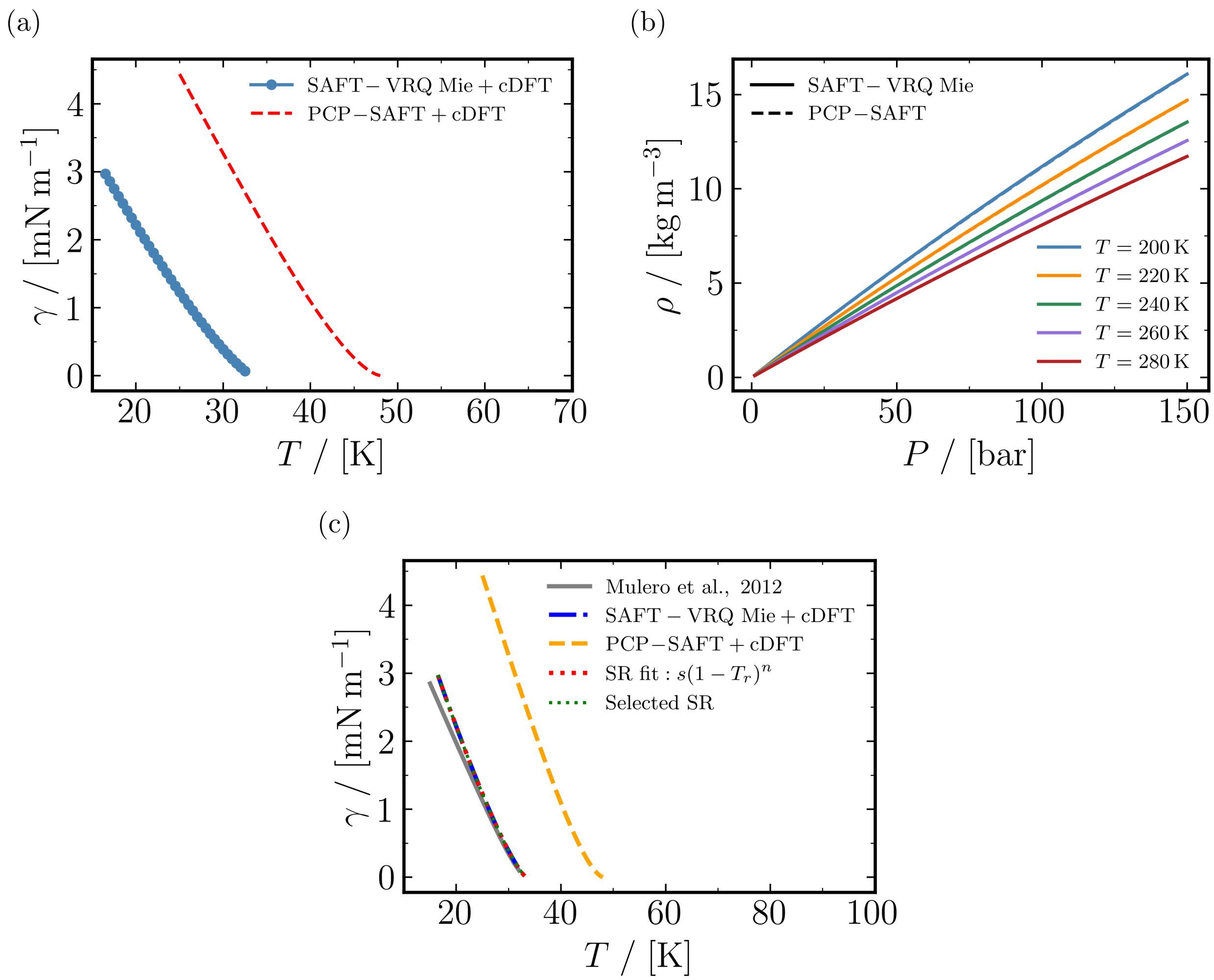

Figure S31: Pure-component interfacial tension (IFT) analysis for H_2. Panel (a) shows the IFTs, $\gamma(T)$, obtained from SAFT-VRQ Mie + cDFT[7,63] and PCP-SAFT + cDFT.[1,5,7] Panel (b) reports the densities for temperatures ranging from 200 to 280 K as a function of pressure, $\rho(P)$, computed using the SAFT-VRQ Mie[63] and PCP-SAFT EoSs. In Panel (c), the GK reference correlation, parameterized using the fit parameters adopted from Mulero et al.,[59] is compared with the SAFT-VRQ Mie + cDFT[7,63] and PCP-SAFT + cDFT data and the selected and canonical SR correlations.

S5.2 Hybrid interfacial tension (IFT) model: cDFT–WSD residual correction for CO_2-rich mixtures

This subsection presents the feature-engineering procedure, search settings, complete Pareto frontiers, and cross-validation performance of the Symbolic Regression (SR)-based hybrid IFT model discussed in the main text. The SR model is trained to correct the uncorrected Winterfeld Scriven Davis (WSD)[64] IFT estimations, enabling it to reproduce the full Perturbed Chain Polar Statistical Associating Fluid Theory (PCP-SAFT) equation of state (EoS) + classical density functional theory (cDFT) results for multi-component CO_2-rich mixtures using a simple expression. Two feature-engineering strategies are evaluated using the same train/validation/test dataset: a physics-informed 12-feature set, denoted as $\mathcal{F}$, and a composition-only 5-feature set, denoted as $\mathcal{F}_{\mathrm{Comp.}}$.

S5.2.1 Target definition

The baseline IFT model is the uncorrected WSD semi-empirical correlation, $\gamma_{\mathrm{base}} \equiv \gamma_{\mathrm{wsd}}^{\mathrm{UC}}$, while the reference value is the PCP-SAFT EoS + cDFT[1,5,7] prediction, γ_{cDFT}. Instead of regressing γ_{cDFT} directly, we regress the dimensionless relative residual

$$\epsilon_\gamma = \frac{\gamma_{\mathrm{cDFT}} - \gamma_{\mathrm{base}}}{\gamma_{\mathrm{base}}}, \tag{S12}$$

so that the baseline model retains the dominant physical contribution, and SR only learns the remaining correction required when one of the components is in the supercritical phase. The predicted residual is then mapped back to the dimensional IFT as

$$\widehat{\gamma} = \gamma_{\mathrm{base}}\big(1 + \epsilon_\gamma\big), \tag{S13}$$

which allows the reconstructed $\widehat{\gamma}$ metrics to be compared directly with the GPR[65] and SVGP[66–68] residual surrogates trained on the same data.

S5.2.2 Feature engineering

Impurities are defined as all components in the mixture other than CO_2. The liquid-phase, vapor-phase, and feed impurity sums are given by $x_{\mathrm{imp}} = \sum_{i \neq \mathrm{CO_2}} x_i$, $y_{\mathrm{imp}} = \sum_{i \neq \mathrm{CO_2}} y_i$, and $z_{\mathrm{imp}} = \sum_{i \neq \mathrm{CO_2}} z_i$, respectively, together with the phase-partitioning difference $\Delta_{\mathrm{imp}} = x_{\mathrm{imp}} - y_{\mathrm{imp}}$ and the impurity partition coefficient $\ell_K = \ln[(y_{\mathrm{imp}} + \epsilon)/(x_{\mathrm{imp}} + \epsilon)]$, where $\epsilon = 10^{-12}$. These five composition input features are used in both feature-engineering strategies. In the physics-informed 12-feature set, denoted as $\mathcal{F}$, the composition input features are supplemented with reduced state variables referenced to pure CO_2, motivated by the strong dependence of IFT residuals on critical-point proximity and the density gap. These additional input features are $T_r^* = T/T_{c,\mathrm{CO_2}}$, $(1 - T_r^*) = 1 - T_r^*$, $P_r^* = P/P_{c,\mathrm{CO_2}}$, $\Pi^* = P/P^0_{\mathrm{sat}}$, $\Delta\rho = \rho_l - \rho_v$, $\Delta\rho^* = (\rho_l - \rho_v)/(\rho_l^0 - \rho_v^0)$, and $\gamma_r^* = \gamma_{\mathrm{wsd}}/\gamma^0_{\mathrm{CO_2}}$. In contrast, the composition-only 5-feature set, denoted as $\mathcal{F}_{\mathrm{Comp.}}$, retains only the five composition aggregates, $\{z_{\mathrm{imp}}, x_{\mathrm{imp}}, y_{\mathrm{imp}}, \Delta_{\mathrm{imp}}, \ell_K\}$, while deliberately excluding all state and density-gap variables to obtain a maximally interpretable, composition-driven correction. The SR search minimizes the mean squared error of ϵ_γ, reported as the loss L. Since L is a squared quantity, we also report RMSE $= \sqrt{L}$, which is of the same unit as ϵ_γ. To rank Pareto-optimal candidates, we use the per-complexity score, defined as the rate at which the loss decreases when one unit of complexity c is added:

$$\mathrm{score}_i = -\frac{\ln L_i - \ln L_{i-1}}{c_i - c_{i-1}}. \tag{S14}$$

A large score indicates that the added complexity is justified by the corresponding gain in accuracy, whereas a score approaching zero suggests that further complexity contributes little and may mainly reflect overfitting. Therefore, the final model is selected as the best compromise between a low Root Mean Square Error (RMSE) and a still-meaningful score, rather than as the lowest-loss, highest-complexity expression. The SR search settings used for the two feature sets are summarized in Table S8. The physics-informed feature set $\mathcal{F}$ was

allowed a larger population size and maximum expression size than the composition-only feature set $\mathcal{F}_{\text{Comp.}}$, while both searches used the same parsimony coefficient and batching size.

Table S8: Symbolic Regression (SR)[60] search configuration used for the physics-informed feature set, $\mathcal{F}$, and the composition-only feature set, $\mathcal{F}_{\text{Comp.}}$, in the hybrid IFT + WSD[64] ML model.

	**$\mathcal{F}$: physics-informed**	**$\mathcal{F}_{\text{Comp.}}$: composition-only**
Binary operators	$+ - \times$, `safepow`, `safe_div`	$+ - \times$, `safepow`, `safe_div`
Unary operators	`abs`, `safe_sqrt`, `safe_log`	`abs`
Populations	120	80
Max. size	24	16
Parsimony	0.003	0.003
Batching	1000	1000

S5.2.3 Pareto frontiers

Tables S9 and S10 list the complete Pareto frontiers obtained for the physics-informed feature set, $\mathcal{F}$, and the composition-only feature set, $\mathcal{F}_{\text{Comp.}}$, respectively. For $\mathcal{F}$, the score defined in Eq. (S14) is large at low complexity, corresponding to the constant expression and the $\ln(\Delta\rho^*)$ family, and then decreases steeply beyond complexity 7. Around complexity 10, the frontier transitions to a rational form in which $\ln(\Delta\rho^*)$ is divided by a pressure/composition-dependent denominator. We select the complexity-12 expression, marked by ★, because it is the simplest equation on this rational branch and lies near the point of diminishing returns on the Pareto frontier. In addition, it is free from empirical constants. This model reaches $R^2 = 0.941$ (as shown in Fig. S32a) with less than half the complexity of the most accurate complexity-24 expression, for which the additional terms provide only a further ca. 1.5 percentage-point gain in R^2. For $\mathcal{F}_{\text{Comp.}}$, the frontier is dominated by a sharp score increase at complexity 2 and a secondary gain at complexity 4. Beyond complexity 4, the RMSE plateaus near 0.027, so the complexity-4 linear law, marked by ★, is selected as the final composition-only model.

Table S9: Pareto frontier obtained for the physics-informed 12-feature set, $\mathcal{F}$, in the Symbolic Regression (SR)-based hybrid IFT model. The complexity c denotes the symbolic expression size, L is the SR loss defined as the mean squared error of the relative residual ϵ_γ, and the reported Root Mean Square Error (RMSE) and Mean Absolute Error (MAE) are evaluated on the training set. The score is computed using Eq. (S14) and measures the reduction in loss per added unit of complexity. The selected expression is highlighted and marked with ★.

c	RMSE	MAE	L	score	Equation
1	0.0849	0.0740	7.203×10^{-3}	–	$\Delta\rho$
2	0.0431	0.0342	1.855×10^{-3}	1.357	-0.0526
3	0.0239	0.0178	5.698×10^{-4}	1.180	$\ln(\Delta\rho^*)$
4	0.0170	0.0130	2.890×10^{-4}	0.679	$\Delta\rho^* - 1.0183$
5	0.0141	0.0085	1.983×10^{-4}	0.377	$\ln(\Delta\rho^*) - x_{\text{imp}}$
6	0.0127	0.0084	1.624×10^{-4}	0.199	$\Delta\rho^* - (x_{\text{imp}} + 0.9979)$
7	0.0124	0.0083	1.526×10^{-4}	0.062	$\big(\ln(\Delta\rho^*) - x_{\text{imp}}\big)\,\Delta\rho^*$
9	0.0123	0.0082	1.510×10^{-4}	0.005	$(\Delta\rho^* - \Delta\rho)\,\ln(\Delta\rho^* - x_{\text{imp}})$
10	0.0111	0.0075	1.235×10^{-4}	0.202	$\ln(\Delta\rho^*)\,/\,\sqrt{P_r^*\,\gamma_r^*}$
12	0.0104	0.0071	1.082×10^{-4}	0.066	$\ln(\Delta\rho^*)\,/\,\big[\sqrt{P_r^*} + (\gamma_r^* - \Delta\rho^*)\big]$ ★
15	0.0097	0.0067	9.497×10^{-5}	0.044	${P_r^*}^{(1-T_r^*)-y_{\text{imp}}}\,T_r^*\,\big(\ln(\Delta\rho^*) - x_{\text{imp}}\big)$
16	0.0097	0.0066	9.410×10^{-5}	0.009	${P_r^*}^{-0.502\,y_{\text{imp}}}\,T_r^*\,\big(\ln(\Delta\rho^*) - x_{\text{imp}}\big)$
17	0.0097	0.0065	9.356×10^{-5}	0.006	$T_r^*\,\ln(\Delta\rho^* - x_{\text{imp}})\,\sqrt{{P_r^*}^{\Delta_{\text{imp}}} - z_{\text{imp}}}$
18	0.0094	0.0065	8.893×10^{-5}	0.051	${P_r^*}^{(1.491-\gamma_r^*)\Delta_{\text{imp}}}\,T_r^*\,\big(\ln(\Delta\rho^*) - x_{\text{imp}}\big)$
19	0.0093	0.0061	8.569×10^{-5}	0.037	$\ln(\Delta\rho^*)\,/\,\big[{P_r^*}^{0.266} + \ell_K\big((\gamma_r^* - \Delta\rho^*) - z_{\text{imp}}\big)\big]$
21	0.0090	0.0060	8.047×10^{-5}	0.031	$\ln(\Delta\rho^*)\,/\,\big[{P_r^*}^{0.279} + \ell_K\big(\gamma_r^*(\gamma_r^* - \Delta\rho^*) - z_{\text{imp}}\big)\big]$
23	0.0089	0.0059	7.878×10^{-5}	0.011	$\ln(\Delta\rho^*)\,/\,\big[(\ell_K - \Delta\rho^*)(\gamma_r^* - \Delta\rho^*) + {P_r^*}^{0.352} - 2z_{\text{imp}}\big]$
24	0.0088	0.0058	7.742×10^{-5}	0.017	$\ln(\Delta\rho^*)\,/\,\big[{P_r^*}^{0.279} + \ell_K\,\gamma_r^*\big((\gamma_r^* - \Delta\rho^*) - z_{\text{imp}}\big)\big] - 0.0021$

Table S10: Pareto frontier obtained for the composition-only 5-feature set, $\mathcal{F}_{\text{Comp.}}$, in the Symbolic Regression (SR)-based hybrid IFT model. The columns follow the same definitions as in Table S9: c is the symbolic complexity, L is the mean squared error of ϵ_γ, Root Mean Square Error (RMSE) and Mean Absolute Error (MAE) are training-set errors, and the score quantifies the loss reduction per added unit of complexity. The selected composition-only expression is highlighted and marked with ★.

c	RMSE	MAE	L	score	Equation
1	0.0909	0.0730	8.271×10^{-3}	–	x_{imp}
2	0.0431	0.0342	1.855×10^{-3}	1.495	-0.0526
4	0.0270	0.0180	7.291×10^{-4}	0.467	$-2.5550\, x_{\text{imp}}$ ★
5	0.0270	0.0180	7.291×10^{-4}	0.000	$x_{\text{imp}}/(-0.3914)$
6	0.0270	0.0179	7.277×10^{-4}	0.002	$x_{\text{imp}}(z_{\text{imp}} - 2.5972)$
7	0.0269	0.0178	7.222×10^{-4}	0.008	$x_{\text{imp}}/(-0.3492 - z_{\text{imp}})$
8	0.0268	0.0177	7.161×10^{-4}	0.008	$x_{\text{imp}}\big(-2.3588 - \Delta_{\text{imp}}^2\big)$
9	0.0267	0.0177	7.151×10^{-4}	0.001	$-2.3785\, x_{\text{imp}} + 0.0158\, \Delta_{\text{imp}}$
10	0.0265	0.0175	7.007×10^{-4}	0.020	$-3.3478\, y_{\text{imp}}^{y_{\text{imp}}}\, x_{\text{imp}}$
11	0.0263	0.0174	6.925×10^{-4}	0.012	$\big(-2.3320 - \lvert -0.3671 - \Delta_{\text{imp}} \rvert\big) x_{\text{imp}}$
12	0.0262	0.0173	6.883×10^{-4}	0.006	$(y_{\text{imp}} - z_{\text{imp}})^{y_{\text{imp}}}\, (-3.5244\, x_{\text{imp}})$
13	0.0255	0.0168	6.505×10^{-4}	0.057	$x_{\text{imp}}\big(-1.7925 - \lvert \ell_K \Delta_{\text{imp}} + 1.0744 \rvert\big)$
15	0.0254	0.0166	6.460×10^{-4}	0.003	$x_{\text{imp}}\big(-2.0208 - \lvert y_{\text{imp}} + \Delta_{\text{imp}} \ell_K + 0.6315 \rvert\big)$
16	0.0253	0.0166	6.417×10^{-4}	0.007	$x_{\text{imp}}\big(-1.9235 - \lvert -0.5519 - \ell_K(\Delta_{\text{imp}} + 0.2337) \rvert\big)$

S5.2.4 Selected models and performance

The selected physics-informed correction from $\mathcal{F}$, with complexity 12, and the selected composition-only correction from $\mathcal{F}_{\text{Comp.}}$, with complexity 4, are

$$\epsilon_{\gamma\mathcal{F}} = \frac{\ln(\Delta\rho^*)}{\sqrt{P_r^*} + (\gamma_r^* - \Delta\rho^*)}, \tag{S15}$$

$$\epsilon_{\gamma\mathcal{F}_{\text{Comp.}}} = -2.56\, x_{\text{imp}}. \tag{S16}$$

The physics-informed correction, $\epsilon_{\gamma\mathcal{F}}$, has a compact rational form. Its numerator contains the logarithm of the normalized density gap, $\Delta\rho^*$, which is directly related to the interfacial density contrast, while the denominator combines the reduced-pressure contribution, $\sqrt{P_r^*}$, with the baseline-IFT/density-gap difference, $(\gamma_r^* - \Delta\rho^*)$. Thus, the selected expression links the residual correction to the pressure state, the density contrast, and the baseline WSD prediction without introducing fitted constants. The composition-only correction, $\epsilon_{\gamma\mathcal{F}_{\text{Comp.}}}$, shows that the dominant composition-driven correction is proportional to the liquid-phase impurity fraction. In this simplified model, each percentage point increase in liquid-phase impurity mole fraction lowers the reconstructed IFT relative to the uncorrected WSD baseline by approximately 2.56%. Table S11 reports the performance of the selected models in the dimensionless residual space, ϵ_γ, and after reconstruction to the dimensional IFT using Eq. (S13). The physics-informed model yields substantially lower residual-space errors than the composition-only model, while both models retain high reconstructed-IFT accuracy because the WSD baseline already captures the dominant contribution. Figs. S32 and S33 show the performance of the two selected expressions, Eqs. (S15) and (S16), in the dimensionless residual space, ϵ_γ. The parity plots in Fig. S32 compare the training, validation, and test predictions with the ideal prediction line and report the corresponding per-split R^2 values, while the residual plots in Fig. S33 show the prediction error against the predicted value with the ±RMSE bands for the test and validation splits. The tighter clustering and approximately three-times narrower residual band obtained with $\mathcal{F}$ are consistent with the

ϵ_γ-space metrics in Table S11, confirming that residual-space performance is the most direct measure of how effectively SR learns the missing physics in the uncorrected WSD baseline, even though both models exceed $R^2 = 0.997$ for the reconstructed $\widehat{\gamma}$.

Table S11: Performance of the selected Symbolic Regression (SR)-based hybrid IFT correction models obtained from the physics-informed feature set, $\mathcal{F}$, and the composition-only feature set, $\mathcal{F}_{\text{Comp.}}$. Residual-space metrics are evaluated for the dimensionless relative residual ϵ_γ, whereas reconstructed-IFT metrics are evaluated for $\widehat{\gamma}$ after applying Eq. (S13). The reconstructed-IFT Root Mean Square Error (RMSE) and Mean Absolute Error (MAE) are reported in $\mathrm{mN\,m^{-1}}$.

	$\mathcal{F}$ (test)	$\mathcal{F}_{\text{Comp.}}$ (test)	$\mathcal{F}$ (val)	$\mathcal{F}_{\text{Comp.}}$ (val)
ϵ_γ R^2	0.9408	0.6181	0.9412	0.6207
ϵ_γ RMSE	0.01023	0.02599	0.01028	0.02612
ϵ_γ Mean Absolute Error	0.00698	0.01735	0.00711	0.01749
$\widehat{\gamma}$ R^2	0.99943	0.99765	0.99945	0.99782
$\widehat{\gamma}$ RMSE	0.1418	0.2891	0.1424	0.2824
$\widehat{\gamma}$ Mean Absolute Error	0.0743	0.1618	0.0757	0.1606
Complexity	12	4	12	4

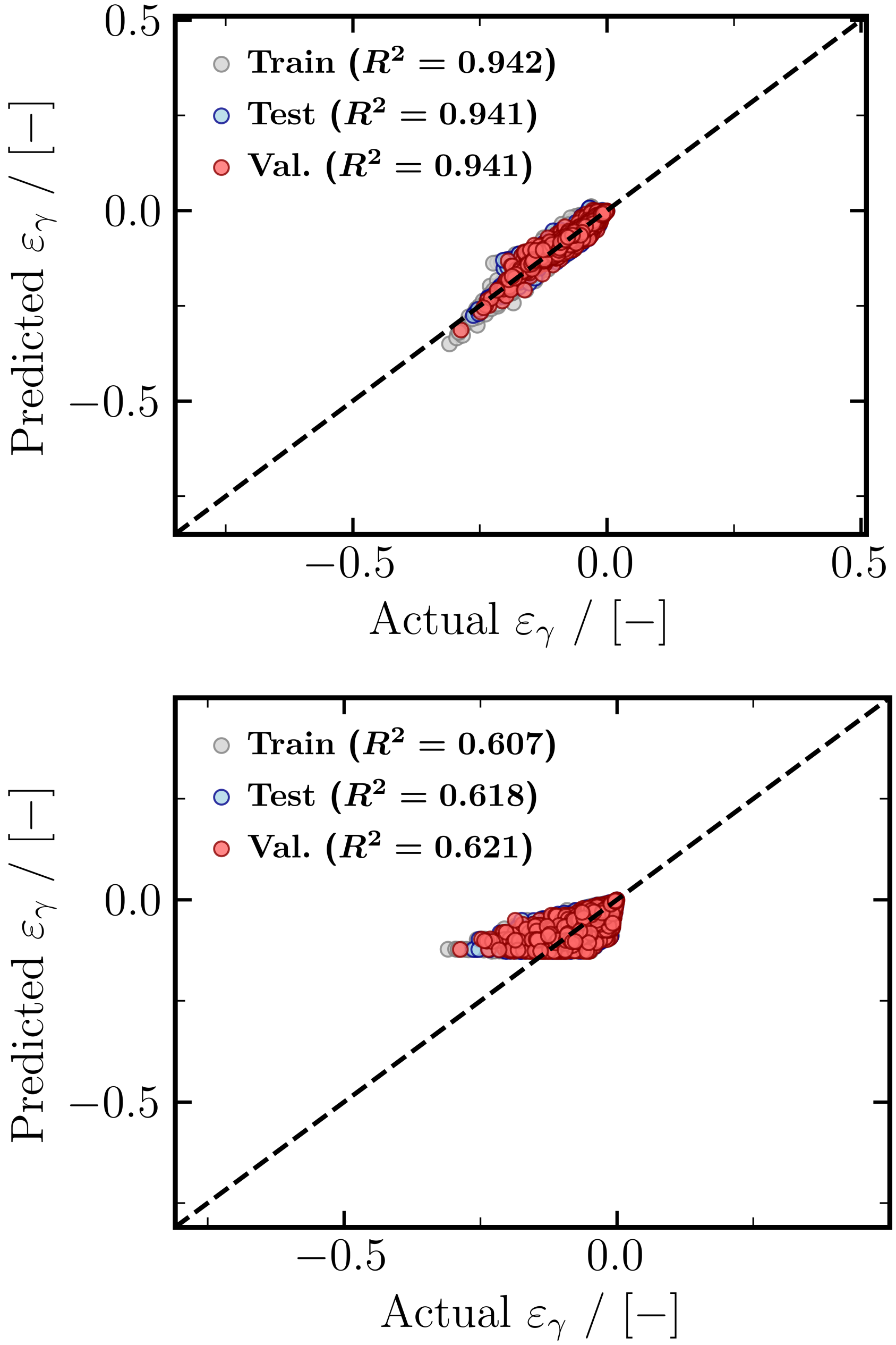

Figure S32: Parity plots of the residual ϵ_γ of the IFT for the selected expressions obtained from (a) the physics-informed feature set, $\epsilon_\gamma = \ln(\Delta\rho^*)/[\sqrt{P_r^*} + (\gamma_r^* - \Delta\rho^*)]$ Eq. (S15), and (b) the composition-only feature set, $\epsilon_\gamma = -2.56\, x_{\mathrm{imp}}$ Eq. (S16). The training, validation, and test splits are distinguished by color, and the per-split R^2 values are reported in each panel.

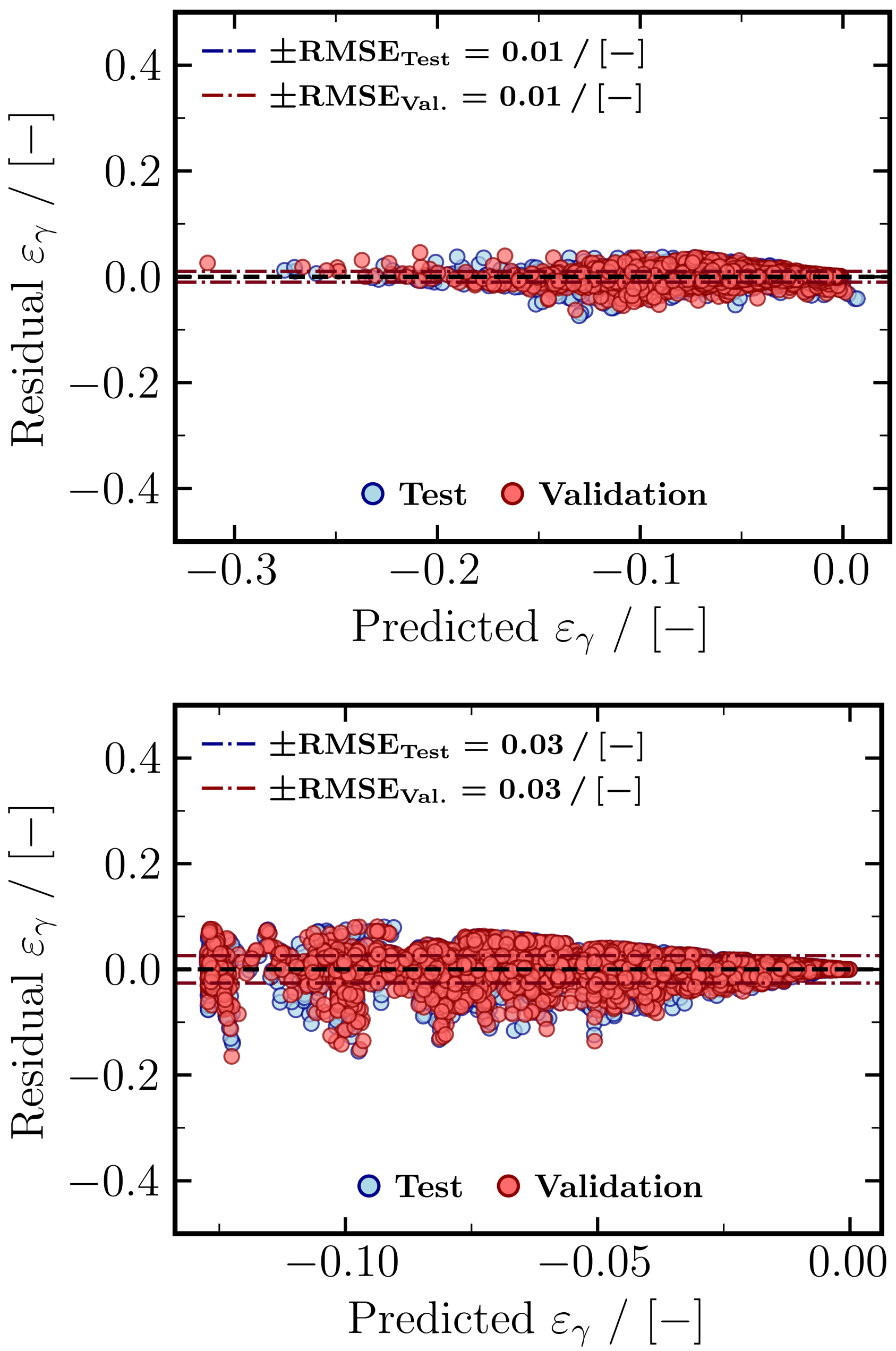

Figure S33: Residual error ϵ_γ of the interfacial tension (IFT) for the selected expressions obtained from (a) the physics-informed feature set, $\epsilon_\gamma = \ln(\Delta\rho^*)/[\sqrt{P_r^*} + (\gamma_r^* - \Delta\rho^*)]$ Eq. (S15), and (b) the composition-only feature set, $\epsilon_\gamma = -2.56\, x_{\mathrm{imp}}$ Eq. (S16). Residuals are plotted against the predicted values, and the dashed lines indicate the ±RMSE ranges for the test and validation splits.

S5.2.5 Cross-validation

To evaluate the robustness of the Symbolic Regression (SR)[60] models beyond a single train/test split, both feature sets discussed in Section S5.2.2 were assessed using five-fold Cross-Validation (CV). In each fold, the complete SR search was repeated. Therefore, the CV results evaluate the generalization ability of the feature set and search procedure, rather than the performance of one fixed symbolic expression. The final expression in each fold was selected using a validation split based on the lowest validation Root Mean Square Error (RMSE). The resulting metrics are reported as mean $\pm 2\sigma$ for the five folds in Table S12.

Table S12: Five-fold Cross-Validation (CV) performance of the Symbolic Regression (SR)-based[60] hybrid IFT models, reported as mean $\pm 2\sigma$ for the folds. The CV was performed using 200 search iterations per fold. Metrics are reported both in the residual space, ϵ_γ, and for the reconstructed IFT, $\widehat{\gamma}$, with reconstructed-IFT RMSE and Mean Absolute Error given in $\mathrm{mN\,m^{-1}}$. The reported metrics include R^2, Root Mean Square Error (RMSE), and Mean Absolute Error. The last row shows the symbolic complexity selected in each fold.

	**$\mathcal{F}$: physics-informed**	**$\mathcal{F}_{\mathrm{Comp.}}$: composition-only**
ϵ_γ R^2	0.961 ± 0.005	0.661 ± 0.014
ϵ_γ RMSE	0.00842 ± 0.00058	0.02494 ± 0.00050
ϵ_γ Mean Absolute Error	0.00568 ± 0.00063	0.01640 ± 0.00051
$\widehat{\gamma}$ R^2	0.99965 ± 0.00009	0.99831 ± 0.00030
$\widehat{\gamma}$ RMSE	0.113 ± 0.015	0.247 ± 0.022
$\widehat{\gamma}$ Mean Absolute Error	0.0603 ± 0.0061	0.1434 ± 0.0146
Complexity/fold	22–24	16

The CV averages are consistent with the single-split results in Table S11, and the narrow 2σ intervals confirm the improved performance of the physics-informed feature set, $\mathcal{F}$, relative to the composition-only feature set, $\mathcal{F}_{\mathrm{Comp.}}$. The CV R^2 for $\mathcal{F}_{\mathrm{Comp.}}$ (0.661) is higher than its single-split test value (0.618) because the fold-wise automatic selection favors more complex expressions with complexity 16. In contrast, the final composition-only expression shown in Eq. (S16) was intentionally selected at complexity 4.

S5.2.6 SVGP-based residual surrogate

The trained Stochastic Variational Gaussian Process Regression (SVGP) surrogate is provided in the supplementary material in the folder `MACHINE_LEARNING/SVGP/`. It is stored as a PyTorch checkpoint, `SVGP_residual_model.pt`, and uses a Matèrn-5/2 kernel with Automatic Relevance Determination (ARD) together with 2000 inducing points.

References

(1) Gross, J. An equation-of-state contribution for polar components: Quadrupolar molecules. *AIChE Journal* **2005**, *51*, 2556–2568.

(2) Alanazi, A.; Bawazeer, S.; Ali, M.; Keshavarz, A.; Hoteit, H. Thermodynamic modeling of hydrogen–water systems with gas impurity at various conditions using cubic and PC-SAFT equations of state. *Energy Conversion and Management: X* **2022**, *15*, 100257.

(3) Esper, T.; Bauer, G.; Rehner, P.; Gross, J. PCP-SAFT Parameters of Pure Substances Using Large Experimental Databases. *Industrial & Engineering Chemistry Research* **2023**, *62*, 15300–15310.

(4) Tang, X.; Gross, J. Modeling the phase equilibria of hydrogen sulfide and carbon dioxide in mixture with hydrocarbons and water using the PCP-SAFT equation of state. *Fluid Phase Equilibria* **2010**, *293*, 11–21.

(5) Gross, J.; Sadowski, G. Perturbed-Chain SAFT: An Equation of State Based on a Perturbation Theory for Chain Molecules. *Industrial & Engineering Chemistry Research* **2001**, *40*, 1244–1260.

(6) Gross, J.; Sadowski, G. Application of the perturbed-chain SAFT equation of state to associating systems. *Industrial & Engineering Chemistry Research* **2002**, *41*, 5510–5515.

(7) Rehner, P.; Bauer, G.; Gross, J. FeO_s: An Open-Source Framework for Equations of State and Classical Density Functional Theory. *Industrial and Engineering Chemistry Research* **2023**, *62*, 5347–5357.

(8) Tsang, C.; Street, W. Phase equilibria in the H_2/CO_2 system at temperatures from 220 to 290 K and pressures to 172 MPa. *Chemical Engineering Science* **1981**, *36*, 993–1000.

(9) Fandiño, O.; Trusler, J. M.; Vega-Maza, D. Phase behavior of (CO_2 + H_2) and (CO_2 + N_2) at temperatures between (218.15 and 303.15) K at pressures up to 15 MPa. *International Journal of Greenhouse Gas Control* **2015**, *36*, 78–92.

(10) Coquelet, C.; Valtz, A.; Dieu, F.; Richon, D.; Arpentinier, P.; Lockwood, F. Isothermal P, x, y data for the argon + carbon dioxide system at six temperatures from 233.32 to 299.21 K and pressures up to 14 MPa. *Fluid Phase Equilibria* **2008**, *273*, 38–43.

(11) Lasala, S.; Chiesa, P.; Privat, R.; Jaubert, J.-N. VLE properties of CO_2–based binary systems containing N_2, O_2 and Ar: experimental measurements and modelling results with advanced cubic equations of state. *Fluid Phase Equilibria* **2016**, *428*, 18–31.

(12) Løvseth, S. W.; Austegard, A.; Westman, S. F.; Stang, H. G. J.; Herrig, S.; Neumann, T.; Span, R. Thermodynamics of the carbon dioxide plus argon (CO_2+Ar) system: A literature review and experimental study of the vapour–liquid and liquid–solid equilibria at temperatures from 253 to 288 K and pressures up to 14 MPa. *Fluid Phase Equilibria* **2018**, *473*, 37–49.

(13) Al-Sahhaf, T. A.; Kidnay, A. J.; Sloan, E. D. Liquid + vapor equilibriums in the N_2 + CO_2 + CH_4 system. *Industrial & Engineering Chemistry Fundamentals* **1983**, *22*, 372–380.

(14) Brown, T.; Niesen, V.; Sloan, E.; Kidnay, A. Vapor-liquid equilibria for the binary systems of nitrogen, carbon dioxide, and n-butane at temperatures from 220 to 344 K. *Fluid Phase Equilibria* **1989**, *53*, 7–14.

(15) Somait, F. A.; Kidnay, A. J. Liquid-vapor equilibria at 270.00 K for systems containing nitrogen, methane, and carbon dioxide. *Journal of Chemical and Engineering Data* **1978**, *23*, 301–305.

(16) Wei, M. S.-W.; Brown, T. S.; Kidnay, A. J.; Sloan, E. D. Vapor + liquid equilibria for the ternary system methane + ethane + carbon dioxide at 230 K and its constituent

binaries at temperatures from 207 to 270 K. *Journal of Chemical and Engineering Data* **1995**, *40*, 726–731.

(17) Petropoulou, E.; Voutsas, E.; Westman, S. F.; Austegard, A.; Stang, H. G. J.; Løvseth, S. W. Vapor–liquid equilibrium of the carbon dioxide/methane mixture at three isotherms. *Fluid Phase Equilibria* **2018**, *462*, 44–58.

(18) Xu, N.; Dong, J.; Wang, Y.; Shi, J. High pressure vapor liquid equilibria at 293 K for systems containing nitrogen, methane and carbon dioxide. *Fluid Phase Equilibria* **1992**, *81*, 175–186.

(19) Fredenslund, A.; Sather, G. A. Gas-liquid equilibrium of the oxygen–carbon dioxide system. *Journal of Chemical & Engineering Data* **1970**, *15*, 17–22.

(20) Christiansen, L. J.; Fredenslund, A.; Gardner, N. In *Advances in Cryogenic Engineering*; Timmerhaus, K. D., Ed.; Springer: Boston, MA, 1974; Vol. 19; pp 309–319, https://doi.org/10.1007/978-1-4613-9847-9_38 (accessed 2026-06-15).

(21) Souza, L. F. S.; Al Ghafri, S. Z. S.; Trusler, J. P. M. Measurement and modelling of the vapor-liquid equilibrium of CO_2 + CO at temperatures between (218.15 and 302.93) K at pressures up to 15 MPa. *Journal of Chemical Thermodynamics* **2018**, *126*, 63–73.

(22) Chapoy, A.; Nazeri, M.; Kapateh, M.; Burgass, R.; Coquelet, C.; Tohidi, B. Effect of impurities on thermophysical properties and phase behaviour of a CO_2-rich system in CCS. *International Journal of Greenhouse Gas Control* **2013**, *19*, 92–100.

(23) Calado, J. C. G.; Streett, W. B. Liquid–vapour equilibrium in the system H_2+Ar at temperatures from 83 to 141 K and pressures to 52 MPa. *Fluid Phase Equilibria* **1979**, *2*, 275–282.

(24) Xiao, J.; Liu, K.; Du, Y.; Jin, Z.; Lu, H. Measurement on and correlation of VLE of H_2-N_2-Ar system. *Huaxue Gongcheng* **1990**, *18*, 8–12.

(25) Streett, W. B.; Calado, J. C. G. Liquid-vapour equilibrium for hydrogen + nitrogen at temperatures from 63 to 110 K and pressures to 57 MPa. *Journal of Chemical Thermodynamics* **1978**, *10*, 1089–1100.

(26) Tsang, C. Y.; Clancy, P.; Calado, J. C. G.; Streett, W. B. Phase equilibria in the H_2/CH_4 system at temperatures from 92.3 to 180.0 K and pressures to 140 MPa. *Chemical Engineering Communications* **1980**, *6*, 365–383.

(27) Baba-Ahmed, A.; Guilbot, P.; Richon, D. New equipment using a static analytic method for the study of vapour–liquid equilibria at temperatures down to 77 K. *Fluid Phase Equilibria* **1999**, *166*, 225–236.

(28) Narinskii, G. B. Liquid-vapour equilibrium in the argon-nitrogen system. I. Experimental data and their verification. *Zhurnal Fizicheskoi Khimii* **1966**, *40*, 2022–2029.

(29) Calado, J. C. G.; Staveley, L. A. K. Excess Gibbs energy of argon + methane liquid mixtures at 115.77 K. *Journal of Chemical Physics* **1972**, *56*, 4718.

(30) Christiansen, L. J.; Fredenslund, A.; Mollerup, J. M. Vapor-liquid equilibrium of the methane-argon, methane-carbon monoxide, and argon-carbon monoxide systems at elevated pressures. *Cryogenics* **1973**, *13*, 405.

(31) Shatskaya, L. V.; Zhirnova, N. A. Liquid-vapour phase equilibria in binary systems at low temperatures: I. Argon + methane system. *Zhurnal Fizicheskoi Khimii* **1976**, *50*, 515.

(32) Sprow, F. B.; Prausnitz, J. M. Vapor-liquid equilibria for five cryogenic mixtures. *AIChE Journal* **1966**, *12*, 780–784.

(33) Narinskii, G. B. Experimental data on liquid-vapor equilibrium in the O_2-Ar system at 90.5 K, 110 K, 120 K. *Kislorodnyi* **1957**, *10*, 9–16.

(34) Clark, A. M.; Din, F.; Robb, J. The liquid-vapour equilibrium of the binary system argon/oxygen. *Proceedings of the Royal Society of London A* **1954**, *221*, 517–534.

(35) Kidnay, A. J.; Miller, R. C.; Parrish, W. R.; Hiza, M. J. Liquid-vapor phase equilibria in the N_2–CH_4 system from 130 to 180 K. *Cryogenics* **1975**, *15*, 531–540.

(36) Torocheshnikov, N. S. Isotherms and isobars of the nitrogen-carbon monoxide system. *Soviet Physics - Technical Physics* **1937**, *4*, 365–369.

(37) Besserer, G. J.; Robinson, D. B. Equilibrium-phase properties of nitrogen-hydrogen sulfide system. *Journal of Chemical and Engineering Data* **1975**, *20*, 157–161.

(38) Yarym-Agaev, N. L.; Sinyavskaya, R. P.; Koliushko, I. I.; Levinton, L. Y. Phase equilibria in the CH_4+H_2S system at temperatures below 273 K. *Ukrainian Chemistry Journal* **1991**, *57*, 701–704.

(39) Coquelet, C.; Stringari, P.; Hajiw, M.; Gonzalez, A.; Montel, F.; Richon, D. Phase equilibrium data for the hydrogen sulphide + methane system at temperatures from 186 to 313 K and pressures up to about 14 MPa. *Fluid Phase Equilibria* **2014**, *383*, 94–99.

(40) Fredenslund, A.; Mollerup, J. M. Gas-liquid equilibrium of hydrogen sulphide + carbon monoxide. *Journal of Chemical Thermodynamics* **1975**, *7*, 677–682.

(41) Hastie, T.; Tibshirani, R.; Friedman, J. *The Elements of Statistical Learning: Data Mining, Inference, and Prediction*, 2nd ed.; Springer: New York, NY, USA, 2009.

(42) Rousseeuw, P. J. Silhouettes: A Graphical Aid to the Interpretation and Validation of Cluster Analysis. *Journal of Computational and Applied Mathematics* **1987**, *20*, 53–65.

(43) Bishop, C. M. *Pattern Recognition and Machine Learning*, 1st ed.; Springer: New York, 2006.

(44) Jolliffe, I. T. *Principal Component Analysis*, 2nd ed.; Springer Series in Statistics; Springer: New York, 2002.

(45) Breiman, L. Random Forests. *Machine Learning* **2001**, *45*, 5–32.

(46) Cortes, C.; Vapnik, V. Support-Vector Networks. *Machine Learning* **1995**, *20*, 273–297.

(47) Drucker, H.; Burges, C. J. C.; Kaufman, L.; Smola, A.; Vapnik, V. Support Vector Regression Machines. *Advances in Neural Information Processing Systems* **1997**, *9*, 155–161.

(48) Chen, T.; Guestrin, C. XGBoost: A Scalable Tree Boosting System. Proceedings of the 22nd ACM SIGKDD International Conference on Knowledge Discovery and Data Mining. 2016; pp 785–794, `https://doi.org/10.1145/2939672.2939785`, Accessed: March 05, 2026.

(49) Chen, T.; He, T.; Benesty, M.; Khotilovich, V.; Tang, Y.; Cho, H.; Chen, K.; Mitchell, R.; Cano, I.; Zhou, T.; Li, M.; Xie, J.; Lin, M.; Geng, Y.; Li, Y.; Yuan, J.; Cortes, D. xgboost: Extreme Gradient Boosting. 2026; Python package version 3.2.1; `https://github.com/dmlc/xgboost`, Accessed: March 05, 2026.

(50) Hollmann, N.; Müller, S.; Purucker, L.; Krishnakumar, A.; Körfer, M.; Hoo, S. B.; Schirrmeister, R. T.; Hutter, F. Accurate predictions on small data with a tabular foundation model. *Nature* **2025**, *637*, 319–326.

(51) Grinsztajn, L.; Flöge, K.; Key, O.; Birkel, F.; Jund, P.; Roof, B.; Jäger, B.; Safaric, D.; Alessi, S.; Hayler, A.; Manium, M.; Yu, R.; Jablonski, F.; Hoo, S. B.; Garg, A.; Robertson, J.; Bühler, M.; Moroshan, V.; Purucker, L.; Cornu, C.; Wehrhahn, L. C.; Bonetto, A.; Schölkopf, B.; Gambhir, S.; Hollmann, N.; Hutter, F. TabPFN-2.5: Advancing the State of the Art in Tabular Foundation Models. 2026; `https://arxiv.org/abs/2511.08667`, Accessed March 05, 2026.

(52) Akiba, T.; Sano, S.; Yanase, T.; Ohta, T.; Koyama, M. Optuna: A Next-Generation Hyperparameter Optimization Framework. Proceedings of the 25th ACM SIGKDD International Conference on Knowledge Discovery and Data Mining. 2019; pp 2623–2631.

(53) Prior Labs TabPFN Extensions: Community extensions for TabPFN. `https://github.com/PriorLabs/tabpfn-extensions`, 2025; Accessed: May 16, 2026.

(54) Pedregosa, F.; Varoquaux, G.; Gramfort, A.; Michel, V.; Thirion, B.; Grisel, O.; Blondel, M.; Prettenhofer, P.; Weiss, R.; Dubourg, V.; Vanderplas, J.; Passos, A.; Cournapeau, D.; Brucher, M.; Perrot, M.; Duchesnay, É. Scikit-learn: Machine Learning in Python. *Journal of Machine Learning Research* **2011**, *12*, 2825–2830.

(55) McCarter, C. The Kernel Density Integral Transformation. *Transactions on Machine Learning Research* **2023**,

(56) Lemmon, E. W.; Bell, I. H.; Huber, M. L.; McLinden, M. O. NIST Standard Reference Database 23: Reference Fluid Thermodynamic and Transport Properties-REFPROP, Version 10.0, National Institute of Standards and Technology. 2018; `https://www.nist.gov/srd/refprop`, Accessed June 9, 2026.

(57) Guggenheim, E. A. The principle of corresponding states. *Journal of Chemical Physics* **1945**, *13*, 253–261.

(58) Cachadiña, I.; Mulero, A.; Tian, J. Surface Tension of Refrigerants—Selection of Data and Recommended Correlations. *Journal of Physical and Chemical Reference Data* **2015**, *44*, 023104.

(59) Mulero, Á.; Cachadiña, I.; Parra, M. I. Recommended Correlations for the Surface Tension of Common Fluids. *Journal of Physical and Chemical Reference Data* **2012**, *41*, 043105.

(60) Cranmer, M. Interpretable Machine Learning for Science with PySR and SymbolicRegression.jl. 2023; https://arxiv.org/abs/2305.01582, Accessed May 9, 2026.

(61) Gross, J.; Vrabec, J. An Equation-of-State Contribution for Polar Components: Dipolar Molecules. *AIChE Journal* **2006**, *52*, 1194–1204.

(62) Müller, E. A.; Ervik, Å.; Mejía, A. A Guide to Computing Interfacial Properties of Fluids from Molecular Simulations [Article v1.0]. *Living Journal of Computational Molecular Science* **2020**, *2*, 21385.

(63) Aasen, A.; Hammer, M.; Ervik, Å.; Müller, E. A.; Wilhelmsen, Ø. Equation of State and Force Fields for Feynman–Hibbs-Corrected Mie Fluids. I. Application to Pure Helium, Neon, Hydrogen, and Deuterium. *Journal of Chemical Physics* **2019**, *151*, 064508.

(64) Winterfeld, P.; Scriven, L.; Davis, H. An approximate theory of interfacial tensions of multicomponent systems: Applications to binary liquid-vapor tensions. *AIChE Journal* **1978**, *24*, 1010–1014.

(65) Rasmussen, C. E.; Williams, C. K. I. *Gaussian Processes for Machine Learning*, 1st ed.; MIT Press: Cambridge, MA, USA, 2006.

(66) Titsias, M. K. Variational Learning of Inducing Variables in Sparse Gaussian Processes. Proceedings of the 12th International Conference on Artificial Intelligence and Statistics (AISTATS). 2009; pp 567–574, http://proceedings.mlr.press/v5/titsias09a.html, Accessed March 12th, 2026.

(67) Hensman, J.; Fusi, N.; Lawrence, N. D. Gaussian Processes for Big Data. Proceedings of the 29th Conference on Uncertainty in Artificial Intelligence (UAI). 2013; pp 282–290, https://arxiv.org/abs/1309.6835, Accessed: March 12, 2026.

(68) Hensman, J.; Matthews, A. G. d. G.; Ghahramani, Z. Scalable Variational Gaussian Process Classification. Proceedings of the 18th International Conference on Artificial

Intelligence and Statistics (AISTATS). 2015; pp 351–360, http://proceedings.mlr.press/v38/hensman15.html, Accessed: March 12, 2026.
